# Well-defined alkali metal catalysts for selective reduction of functionalized esters and carbonyl compounds *via* hydrosilylation

**DOI:** 10.1039/d6ra06630e

**Published:** 2026-10-05

**Authors:** Guoqi Zhang, Paulina Hiyaev, Haiyu Mei, Marilyn Chocho

**Affiliations:** a Department of Sciences, John Jay College and PhD Program in Chemistry, The Graduate Center, The City University of New York New York NY 10019 USA guzhang@jjay.cuny.edu

## Abstract

Well-defined 2,2′:6′,2″-terpyridine-ligated alkali metal complexes catalysed the chemo- and regioselective reduction of esters and carbonyl compounds through hydrosilylation under mild conditions. Unusual regioselectivity on the alkene group was observed for the reduction of α,β-unsaturated esters.

## Abstract 2

Well-defined 2,2′:6′,2″-terpyridine-ligated alkali metal complexes catalysed the chemo- and regioselective reduction of esters and carbonyl compounds through hydrosilylation under mild conditions.

## Graphical abstract

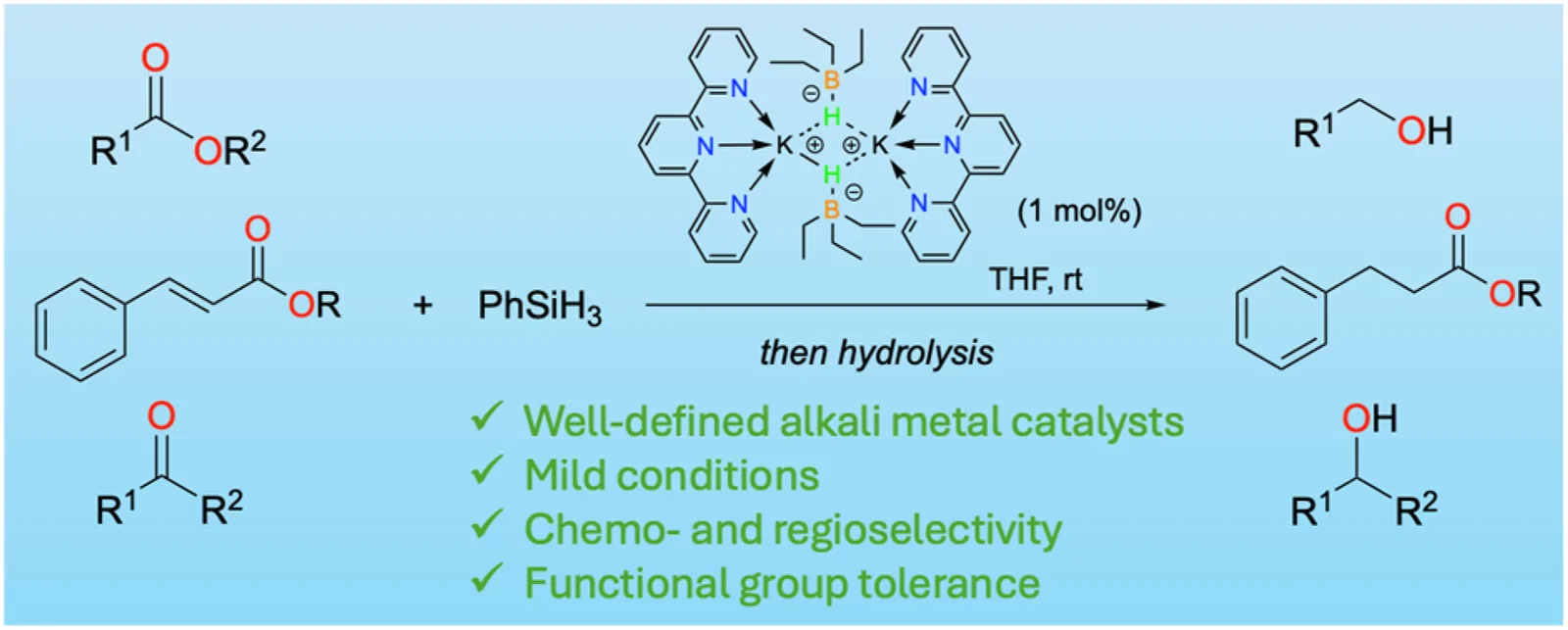

For decades, homogeneous catalysis has been one of the most attractive research areas in organic synthesis and fine chemical industries as it carries out complicated and often challenging chemical transformations in an atom-economical manner.^1,2^ Catalytic hydrogenation and hydrofunctionalization of carbonyl derivatives which provide convenient access to alcohols and other reduced products represent essential catalytic reactions utilizing bulk and inexpensive materials for the synthesis of valuable fine chemicals.^3–5^ While a plethora of practical methods have been developed based on the employment of noble metal catalysts in industry, there were increasing interests in discovering low-cost, earth-abundant metal catalysts enabling high-efficiency hydrogenation and hydrofunctionalization reactions in recent years.^6–8^ In this regard, first-row transition metals such as Fe, Co, Ni, Cu and Mn, *etc.* have been most popularly explored as many have displayed remarkable catalytic efficiency through reasonable ligand design.^9–13^ In contrast, as highly abundant elements, alkali metals (AMs) such as Na and K (ranked 6th and 7th most abundant in the earth's crust, respectively) have been largely underexplored towards these essential catalytic processes.^14–16^

Although ketones and aldehydes are readily reduced by using various hydrogen sources and catalysts, reduction of esters is challenging.^17,18^ Catalytic conversion of esters to alcohols is particularly useful in multistep synthesis of complex molecules of biological importance as well as in chemical industries. Hydrosilylation of esters provides a one-step, mild reduction to approach protected silyl alcohols which could be readily hydrolysed to the corresponding alcohols.^19^ In this process, the safe and easy-to-handle silanes are utilized in replacement of pressurized hydrogen gas used in hydrogenation reaction. However, available catalysts for ester hydrosilylation are still limited thus far. Except for the early contributions mainly focusing on precious metals such as Ru,^20,21^ Rh^22^ and Pd,^23^ recent years have witnessed the development of catalysts based on non-precious transition metals including Ti,^24^ Zr,^25^ Mn,^26–30^ Fe,^31–34^ Co,^35,36^ Ni,^37^ Cu^38^ and Zn^39^ (Scheme 1), with Mn and Fe being most extensively investigated. However, these catalysts require challenging ligand design and often involve sensitive phosphine-containing ligands.

**Scheme 1:**
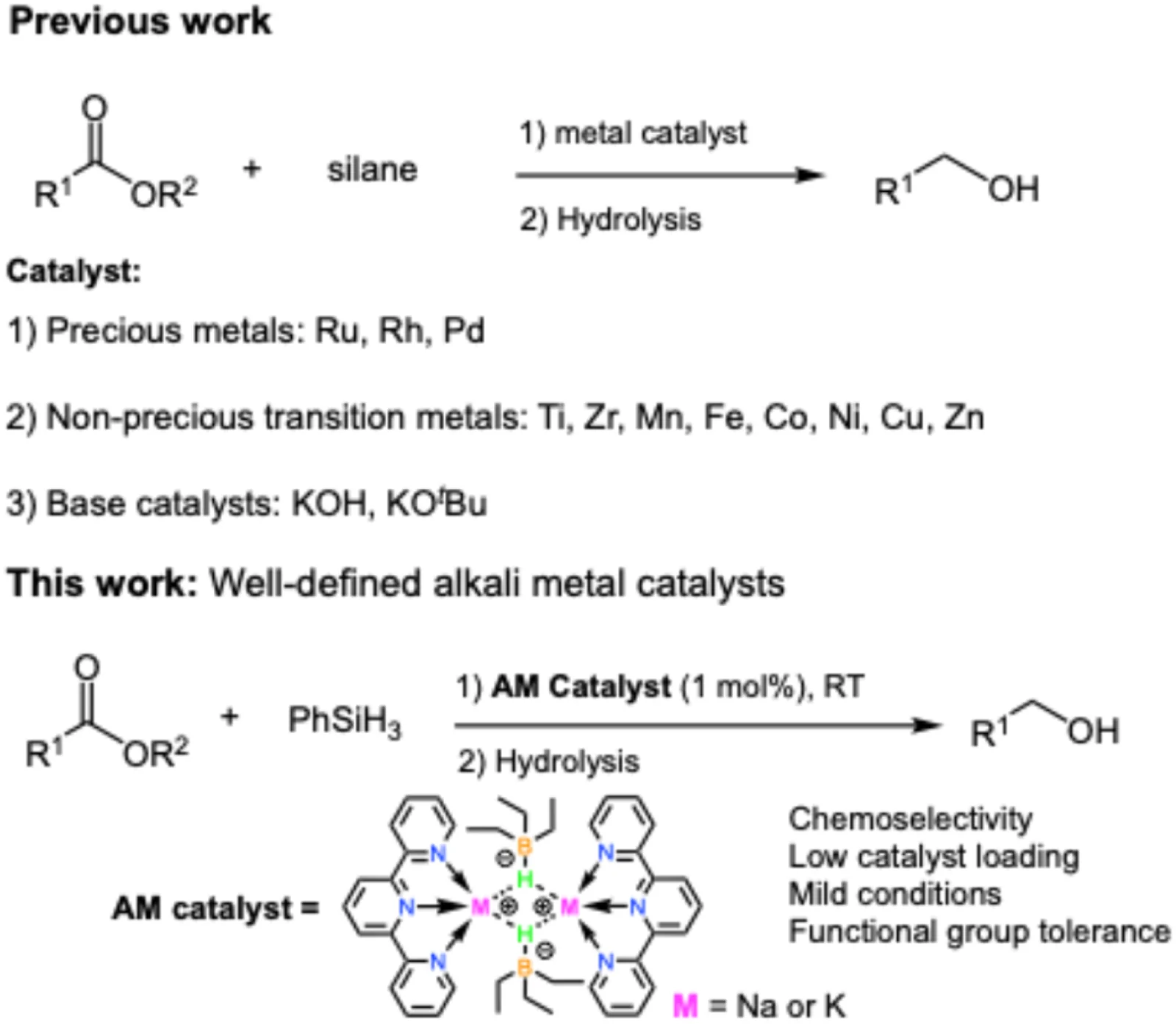
The current state-of-the-art of metal-catalysed reduction of esters by hydrosilylation.

It is worth mentioning that simple base-catalysed hydrosilylation of esters have also been approached by the Nolan and Nikonov groups, respectively. It was reported that both KOH and KO^*t*^Bu (4–10 mol%) could promote the hydrosilylative reduction of esters to alcohols, despite with a limited substrate scope.^40–42^ While the strong basic conditions were determining in activating the silanes during the catalytic cycles, these observations inspired us to further explore whether an AM center could play a role in catalysing the hydrosilylation of esters. In continuation with our recent discovery of novel well-defined AM catalysts (compounds 1–4, Scheme 2) for the high-efficiency and regioselective hydroboration of epoxides using ligated sodium and potassium triethylborohydrides,^43^ where the AM (Na) was found to be critical for the activation of substrates, we report here that such AM catalysts also behave as active catalysts for the selective hydrosilylative reduction of esters and other carbonyl compounds under mild conditions. This represents the first example of well-defined AM catalysts that enable highly efficient and chemo- and regioselective reduction of esters.

**Scheme 2:**
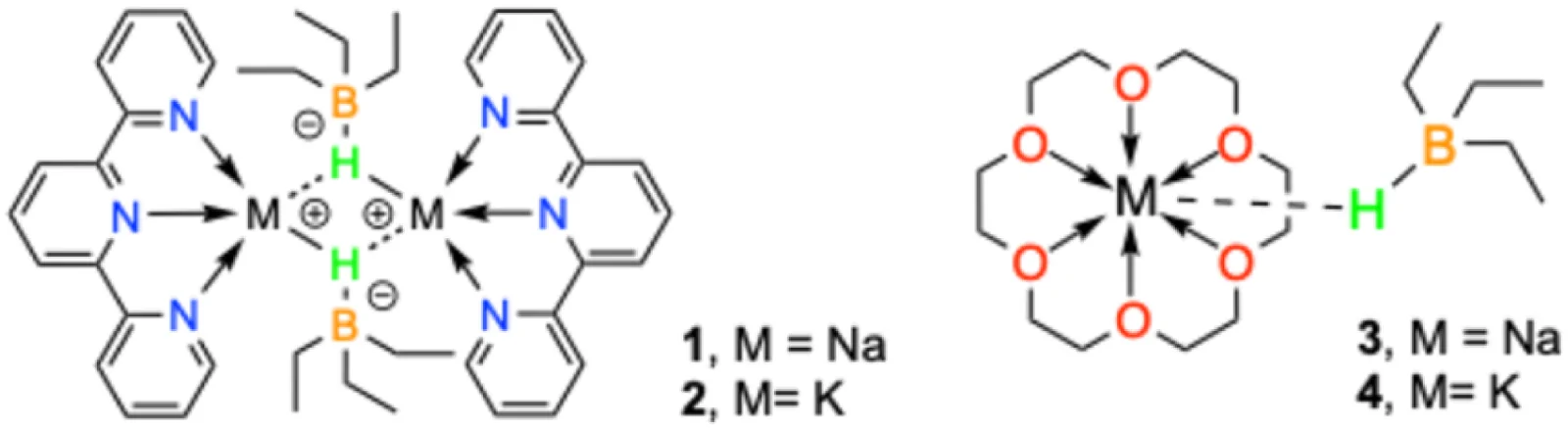
The structures of well-defined AM catalysts 1–4 studied in this work.

AM catalysts 1–4 that were readily prepared^43^ were first examined for the hydrosilylation of a model substrate, methyl benzoate by phenylsilane (PhSiH_3_). Pleasingly, all four catalysts (1 mol%) showed remarkable catalytic activity toward the hydrosilylation of methyl benzoate at room temperature using THF as a solvent, with 2 being the best-performing catalyst (entries 1–4, Table 1), promoting almost quantitative conversion of the ester to benzylic silyl ether which was subsequently hydrolysed to give benzylic alcohol (*vide infra*). Next, the solvent effect was studied when 2 (1 mol%) was used as the catalyst under the same conditions above. Switching solvents from THF to nonpolar solvents such as toluene, benzene and hexane generally led to a significant drop in conversion of the ester (entries 5–7, Table 1), while using polar solvent such as chloroform and diethyl ether, or simply no solvent only slightly affected the conversion (entries 8–10, Table 1). Additionally, using 2 as the catalyst we tested various silanes as reductants such as Ph_2_SiH_2_, Ph_3_SiH, Et_3_SiH, (EtO)_3_SiH and polymethylhydrosiloxane (PMHS), yet the results reveal that all of them showed lower to trace conversions of the ester (entries 11–15, Table 1). This highlights the importance of using a strong silane reductant, PhSiH_3_. Finally, several control experiments have been conducted. When the reaction was carried out without a catalyst, no ester conversion was observed (entry 16, Table 1); while using NaHBEt_3_ or KHBEt_3_ (5 mol%) alone as a catalyst without the presence of a ligand, only moderate conversion of the ester was detected (entries 17 and 18, Table 1). It was noted that similar reactions using NaHBEt_3_ or LiHBEt_3_ as a catalyst have been reported at elevated temperature by the Crabtree group in 2014.^44^ This indicates the role a ligated AM catalyst played in the catalytic cycle. Notably, lowering the catalyst loading amount of 2 to 0.1 mol% also furnished the ester hydrosilylation reaction with 82% conversion (entry 19, Table 1).

**Table 1:** Reactivity test for AM-catalysed hydrosilylation of methyl benzoate^a^

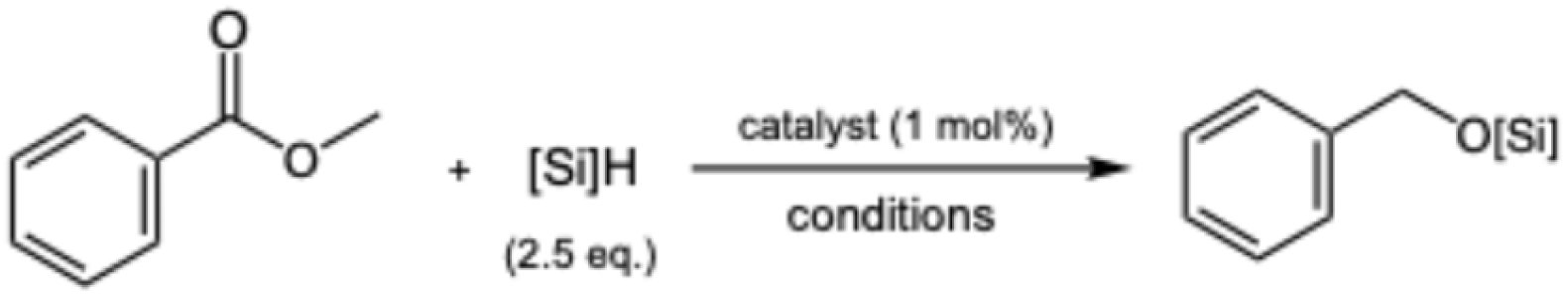				
Entry	Catalyst	[SiH]	Solvent	Conversion^b^/%
1	1	PhSiH_3_	THF	96
2	2	PhSiH_3_	THF	98
3	3	PhSiH_3_	THF	93
4	4	PhSiH_3_	THF	89
5	2	PhSiH_3_	Toluene	49
6	2	PhSiH_3_	Benzene	42
7	2	PhSiH_3_	Hexane	30
8	2	PhSiH_3_	Chloroform	88
9	2	PhSiH_3_	Et_2_O	92
10	2	PhSiH_3_	Neat	90
11	2	Ph_2_SiH_2_	THF	52
12	2	Ph_3_SiH	THF	34
13	2	Et_3_SiH	THF	38
14	2	(EtO)_3_SiH	THF	<5
15	2	PMHS	THF	<5
16	—	PhSiH_3_	THF	0
17^c^	NaHBEt_3_	PhSiH_3_	THF	62
18^c^	KHBEt_3_	PhSiH_3_	THF	67
19^d^	2	PhSiH_3_	THF	82

a Conditions: methyl benzoate (0.5 mmol), silane (1.25 mmol, 2.5 eq.), catalyst (1 mol%) and solvent (1 mL), rt, N_2_, 8 h.

b Conversion of methyl benzoate determined by GC analysis using hexamethylbenzene as an internal standard.

c 5 mol% catalyst was used.

d 0.1 mol% of 1 was used.

Having demonstrated the best catalytic efficiency of potassium catalyst 2 for the hydrosilylation of methyl benzoate, we explored the generality of this catalyst for a range of esters, even though catalyst 1 displayed comparable catalytic performance from Table 1. The results are summarized in Table 2. First, the silylated product from the conversion of methyl benzoate (5a) was subject to hydrolysis under basic conditions (see details, SI) and the final alcohol product (90% yield) was isolated by column chromatography. Both electron-withdrawing 2- or 4-chloro substituted methyl benzoate (5b and 5c) and electron-donating 4-methoxy substituted substrate (5d) were found to be excellent substrates for the catalytic hydrosilylation, affording the primary alcohols (6b–d) in 84–91% yields after hydrolysis. Next, methyl benzoates containing reducible functional groups (cyano and nitro) were examined for the chemoselective hydrosilylation and the results proved that these functional groups were well tolerant from the ester reduction and the corresponding alcohols (6e and 6f) were isolated in good yields. In addition, naphthyl-substituted aliphatic ester (5g) and a full alkyl ester (5h) turned out to be suitable substrates with complete conversion under standard conditions, even though relatively lower yield of alcohol (6h) was obtained for the latter. Relevant carbonate derivatives (5j and 5k) were explored for hydrosilylation under the same conditions, phenol and benzylic alcohol have been observed as one of the products by GC analysis, respectively. Surprisingly, when α,β-unsaturated esters were employed as substrates, regioselective reduction occurred only on the alkene unit to provide saturated esters through transfer hydrogenation, with the ester remaining intact. Such regioselectivity through hydrosilylation is rare and has been previously carried out using Cu(i) catalyst with phosphine ligands in 2004.^45^ Accordingly, several ß-aryl unsaturated esters were converted to the corresponding esters (6k–n) which have been isolated in good yields without base hydrolysis. It is worth noting that methyl, ethyl or benzyl ester here showed almost the same reactivity towards selective alkene reduction. However, the regioselective reduction for a β-disubstituted substrate, ethyl (*E*)-3-phenylbut-2-enoate was found to be sluggish and the product 6o was detected in only 18% yield by GC analysis.

**Table 2:** Substrate scope for 2-catalysed selective reduction of esters with PhSiH_3_ or HBpin^a^

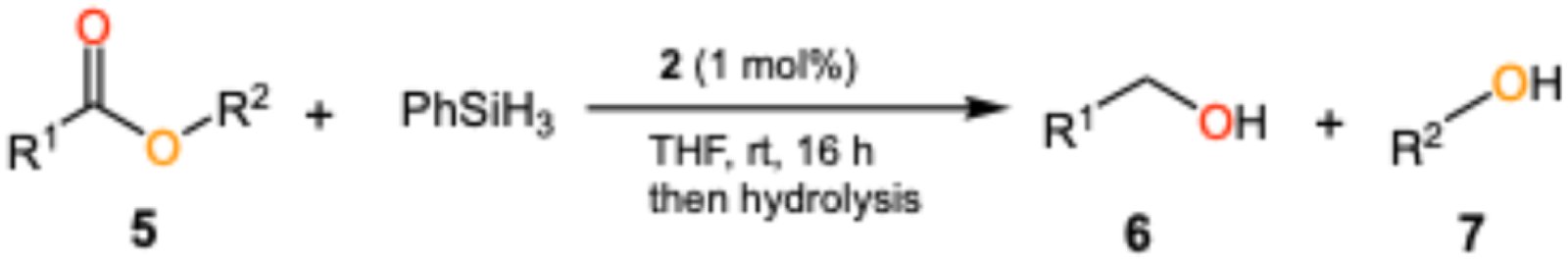			
Entry	Substrate (5)	Product (6 or 7)	Yield^b^/%
1	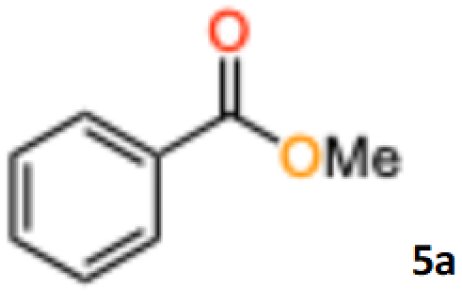	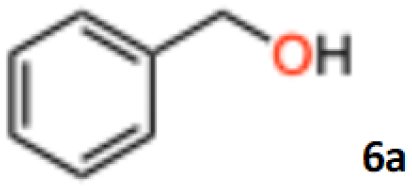	90
2	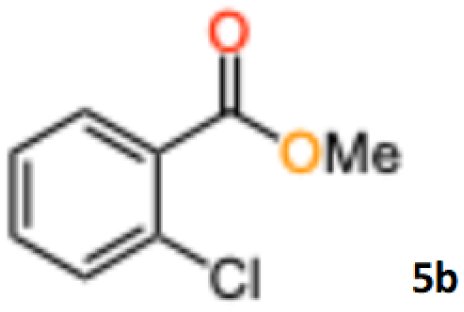	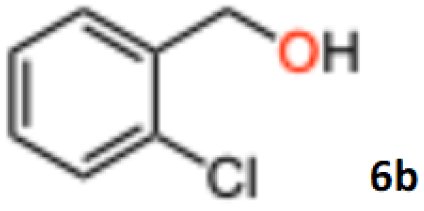	84
3	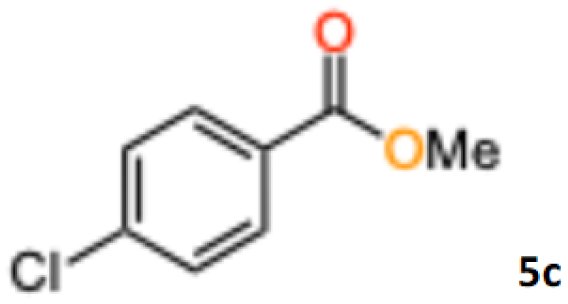	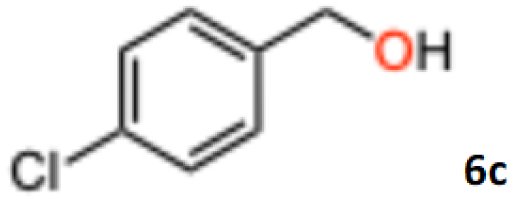	91
4	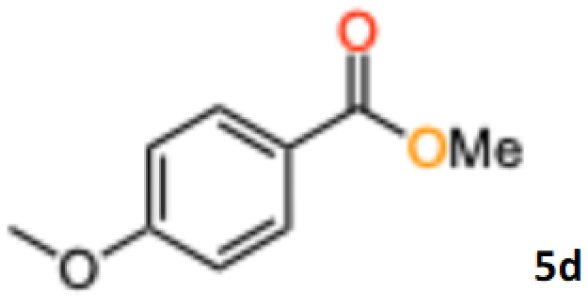	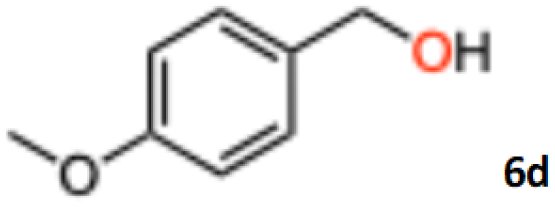	86
5	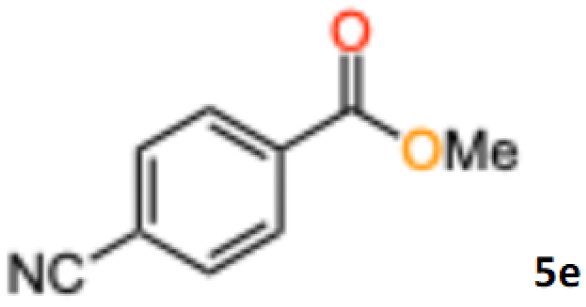	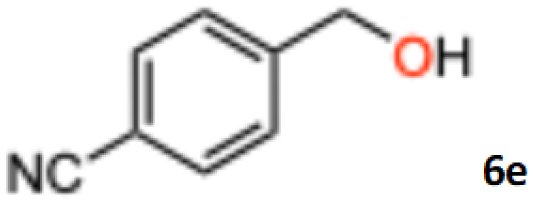	82
6	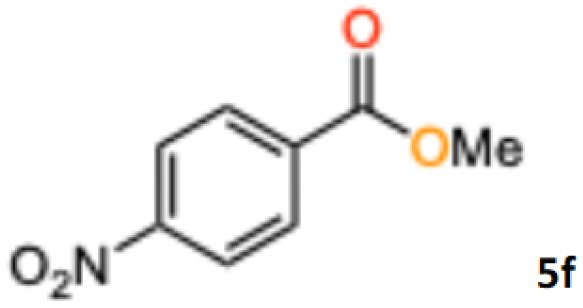	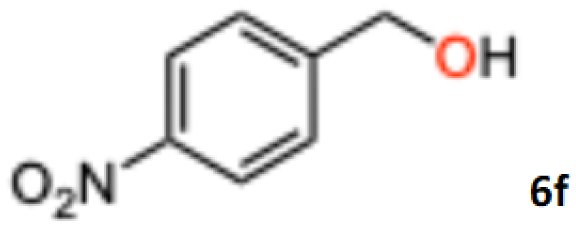	85
7	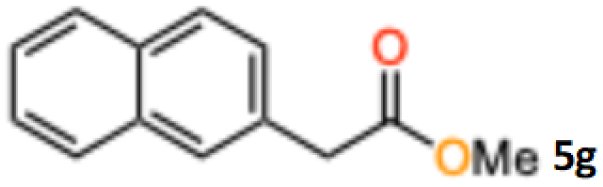	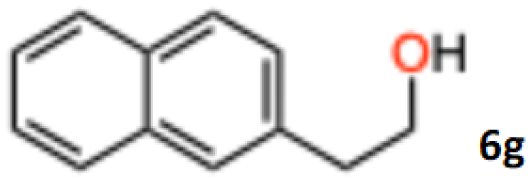	80
8	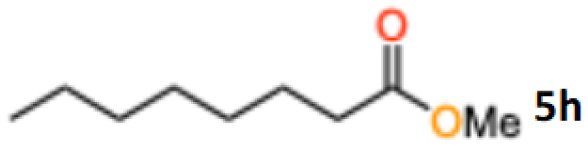	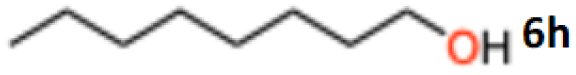	67
9^c^	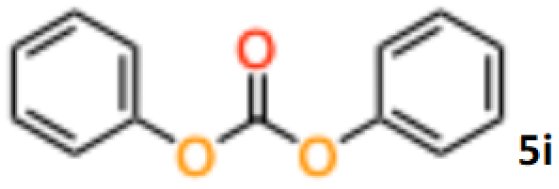	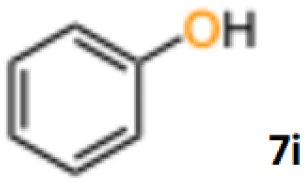	99^c^
10^c^	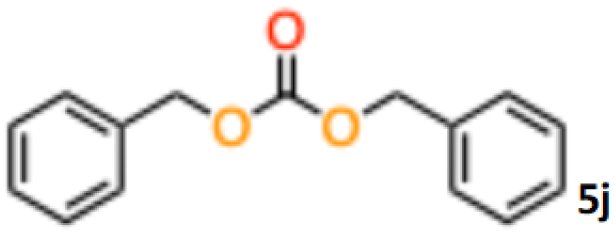	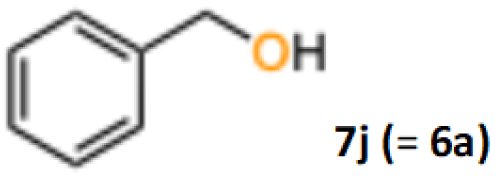	88
11	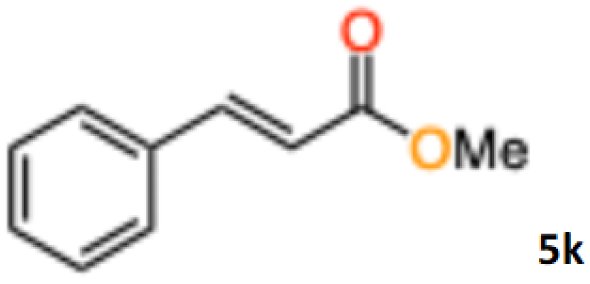	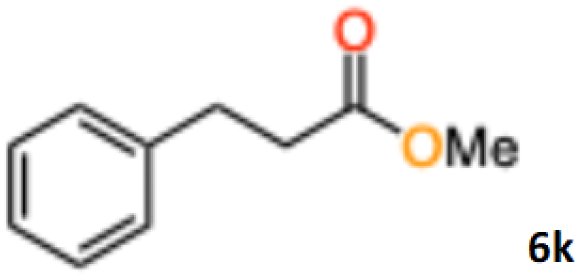	82
12	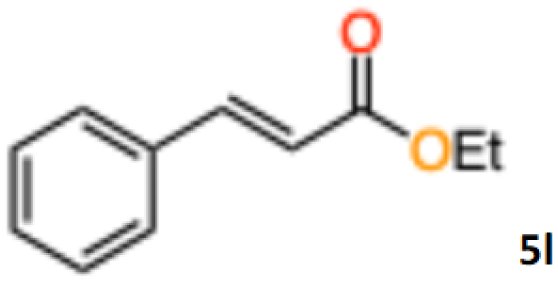	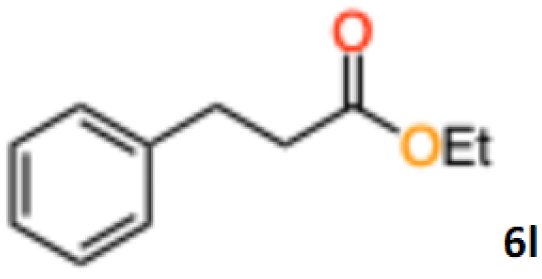	85
13	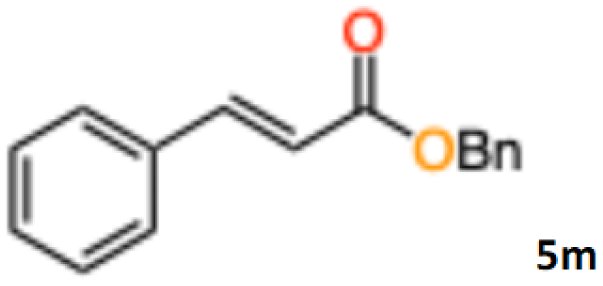	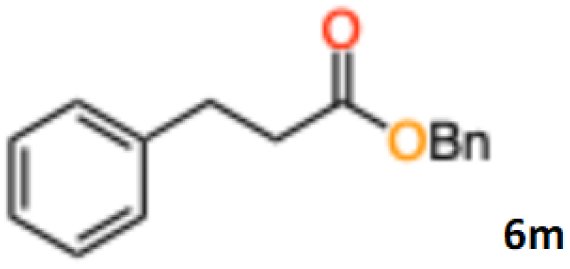	82
14	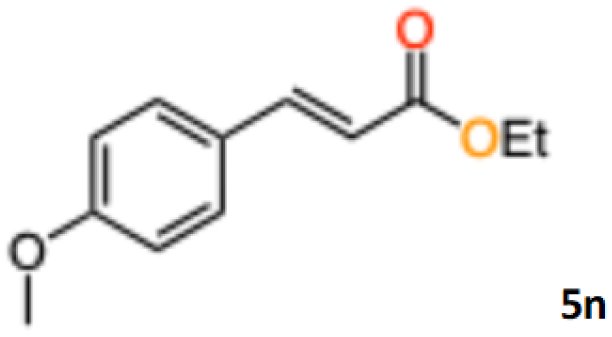	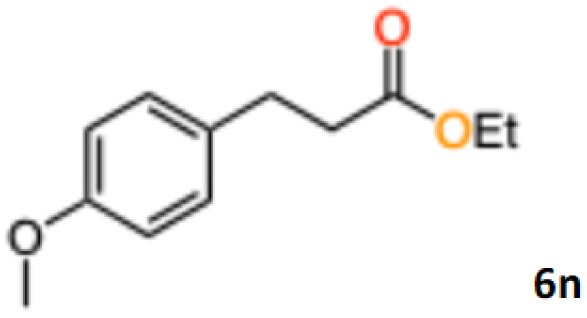	85
15^c^	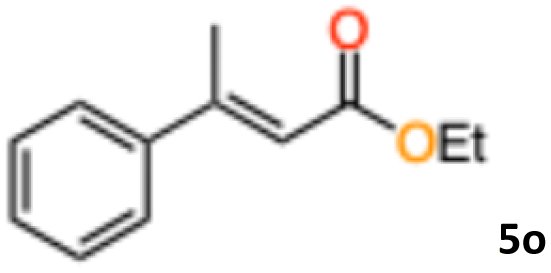	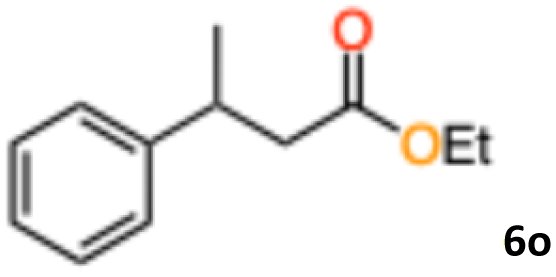	18

a Conditions: ester (1.0 mmol), PhSiH_3_ (2.5 mmol), 2 (1 mol%) and THF (1 mL), rt, N_2_, 16 h.

b Yields of isolated products after hydrolysis.

c Yield determined by GC analysis.

The interesting chemo- and regio-selective hydrosilylation of esters catalyzed by 2 encouraged us to further investigate the selective reduction of some functionalized carbonyl compounds such as aldehydes and ketones which could be more readily reduced. Thus, we examined several carbonyl substrates using 2 (1 mol%) as a catalyst at room temperature and the results are shown in Table 3. Nitro-substituted benzaldehyde was fully converted to benzyl silyl ether in 1 hour, which was subsequently hydrolysed to give alcohol 9a in 92% isolated yield. Aryl ketones 8b and 8c also proceeded well to afford secondary alcohols 9b and 9c in high yields after hydrolysis. Interestingly, unlike the examples of esters (5l–p), α,β-unsaturated ketone 8d was regioselectively reduced on the ketone group instead of alkene part under the same catalytic conditions, which has been frequently observed in reduction processes through hydrofunctionalization. Again, nitro-substituted ketones (8e and 8f) and even the ketone with additional halo substituent at the α-position (8g) were well tolerated under the catalytic conditions to offer alcohols 9e–g with 81–85% yields.

**Table 3:** Substrate scope for 2-catalysed reduction of functionalized carbonyl compounds with PhSiH_3_^a^

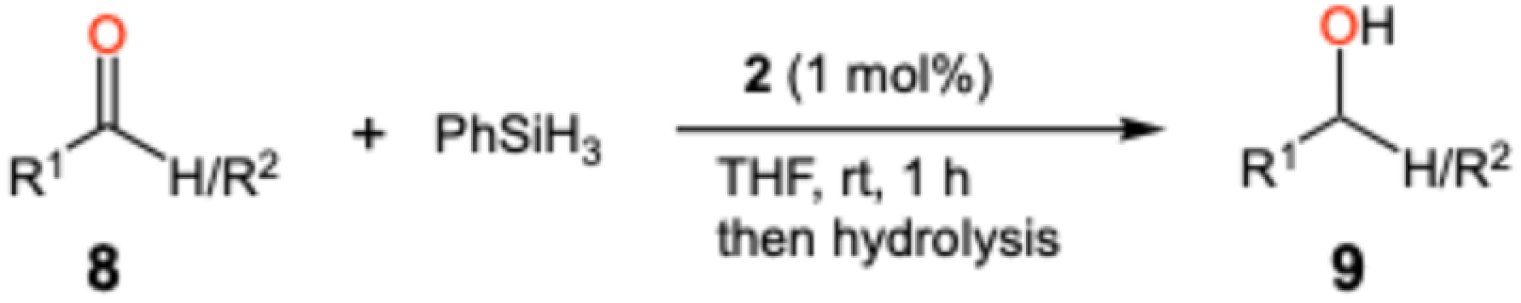			
Entry	Substrate	Product	Yield^b^/%
1	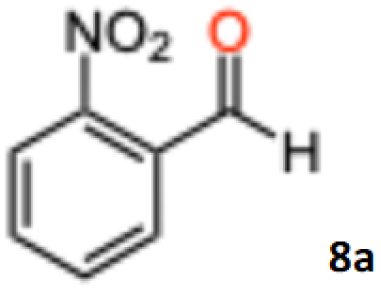	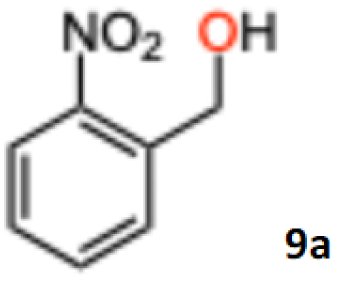	92
2	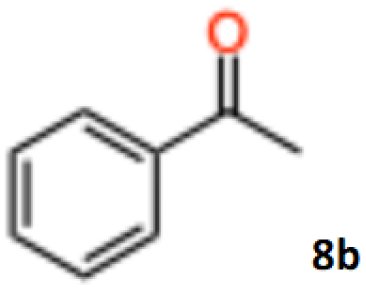	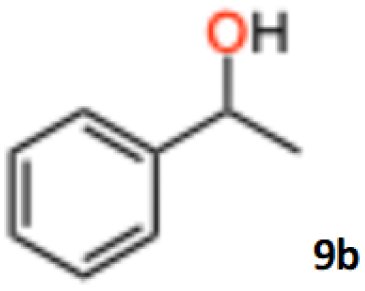	87
3	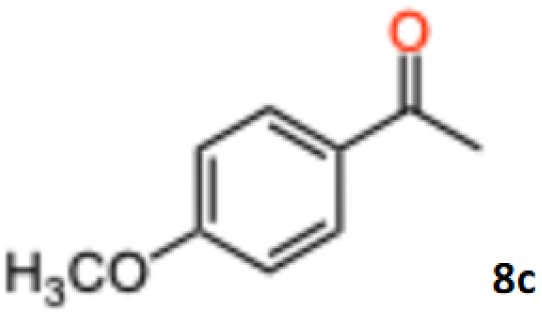	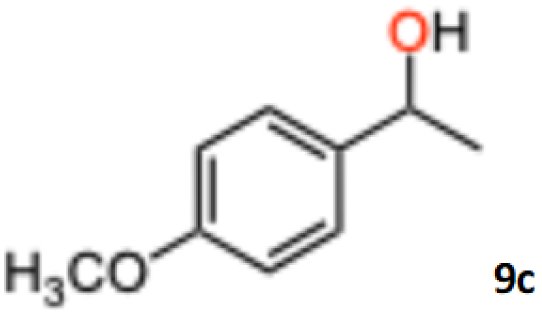	90
4	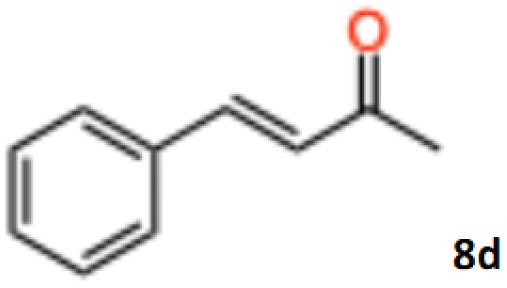	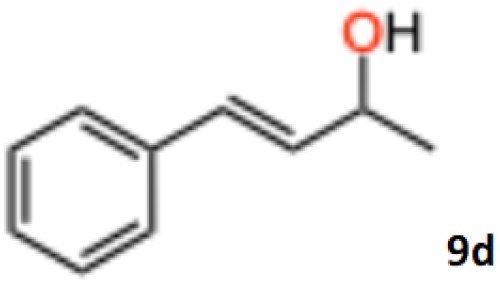	93
5	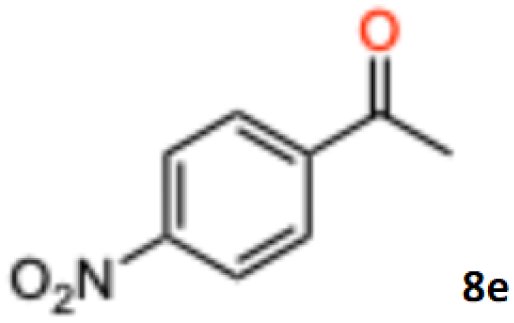	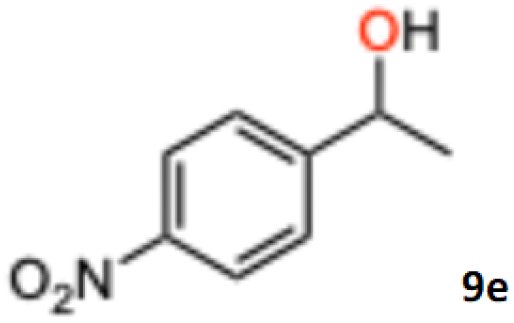	85
6	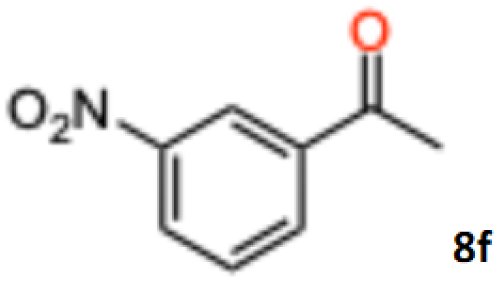	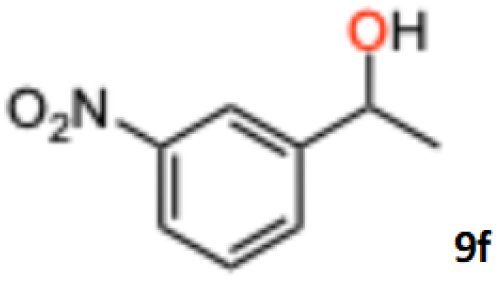	82
7	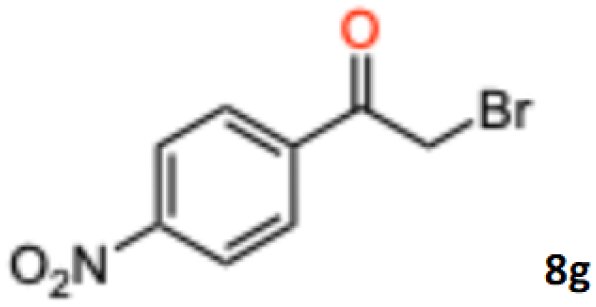	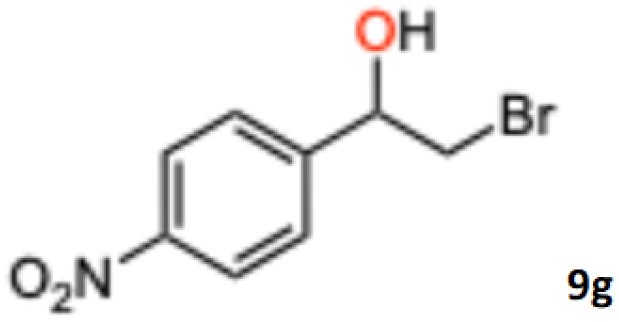	81

a Conditions: aldehyde or ketone (1.0 mmol), PhSiH_3_ (1.1 mmol), 2 (1 mol%), THF (1 mL), rt, N_2_, 1 h.

b Yields of isolated products.

Several additional experiments were conducted to better understand the chemoselective reduction of more challenging nitro and alkene groups by catalyst 2 as well as the general catalytic activity of AM catalysts under some unrestricted conditions (such as in the air or aqueous media). Schemes 3 and 4 illustrated the results from those experiments. While the reduction of nitrobenzene through 2-catalysed hydrosilylation at room temperature was very sluggish (<10% conversion), elevating the temperature to 60 °C led to the direct conversion from nitrobenzene to aniline in 88% yield as detected by GC analysis (Scheme 3a). Furthermore, the hydrosilylation of a bifunctional substrate, 1-nitro-3-vinylbenzene containing both nitro and alkene groups was carried out at 60 °C, and the chemoselective reduction on the nitro group over alkene part was observed, with 3-vinylaniline (10) being isolated in 85% yield (Scheme 3b).

**Scheme 3:**
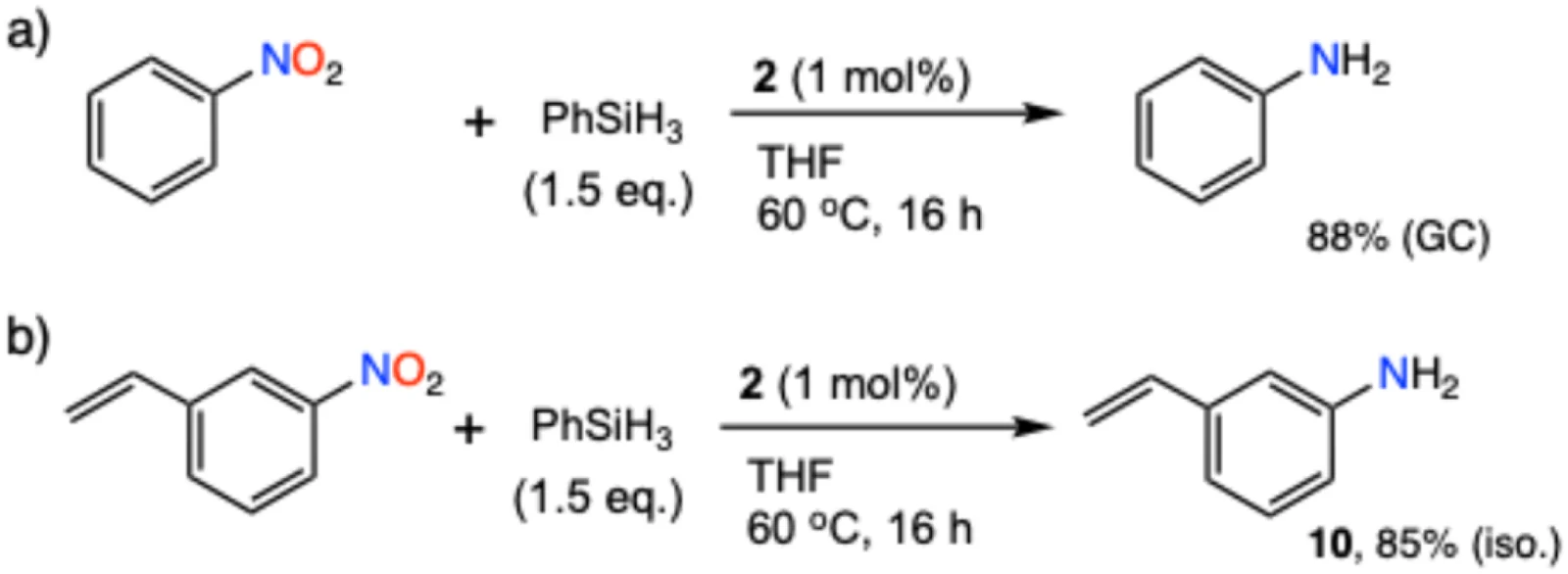
Additional experiments (a and b) for chemoselective nitro group reduction.

**Scheme 4:**
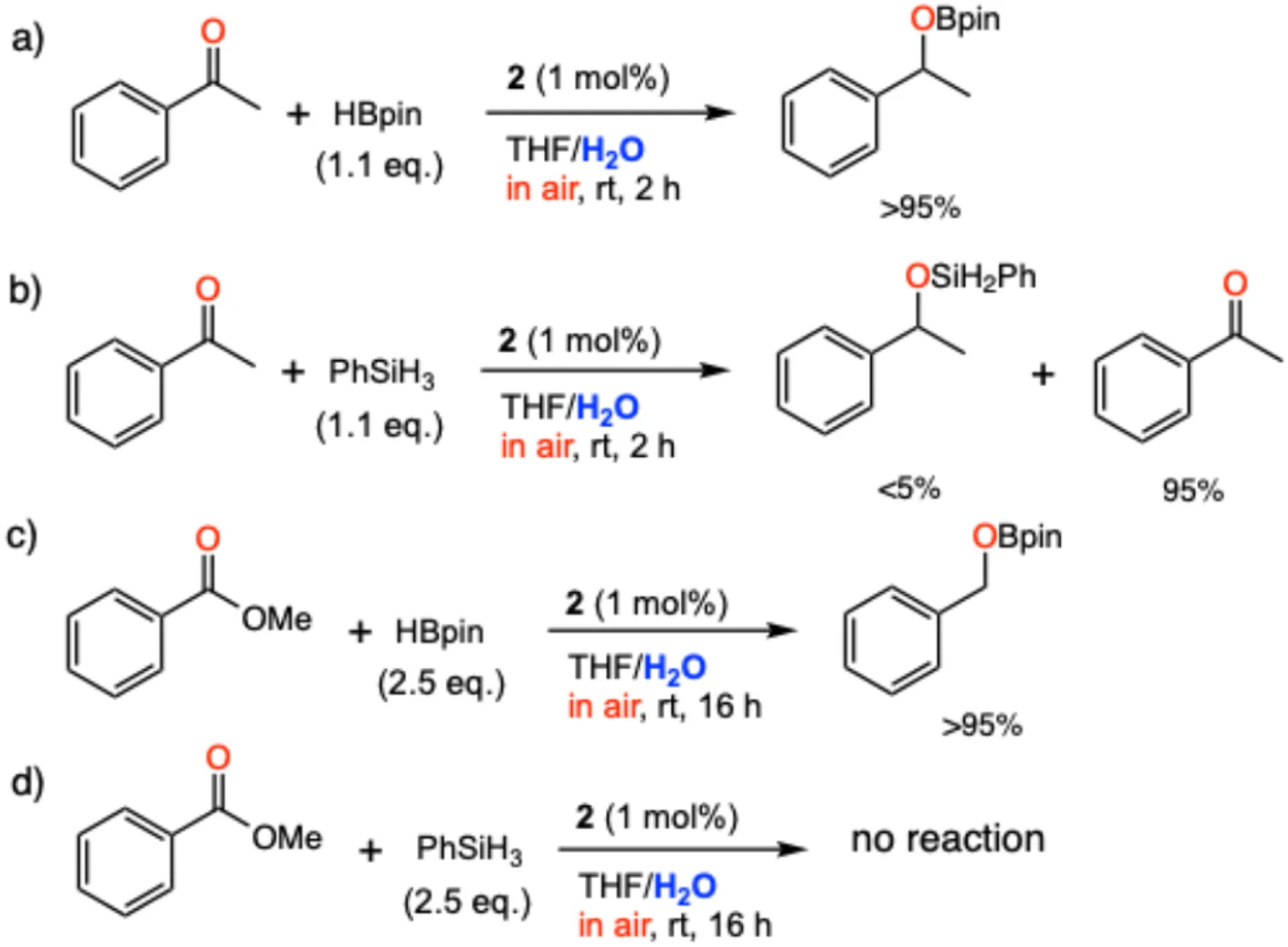
Additional experiments (a–d) for reactions in aqueous media in the air. Conditions: substrate (1.0 mmol), HBpin or PhSiH_3_ (indicated amount), 2 (1 mol%), THF/H_2_O (1 mL, 9 : 1, v/v), rt, air, 2 or 16 h. Yields determined by GC analysis.

Interestingly, catalyst 2 was found to be an excellent catalyst for the hydroboration of both acetophenone and methyl benzoate by pinacolborane (HBpin). The reaction could be completed in an aqueous solvent (THF : H_2_O = 9/1) and in the air to afford boronate esters (Scheme 4a and c). In contrast, the hydrosilylation of both substrates by PhSiH_3_ under the same conditions did not proceed well (Scheme 4b and d). As catalytic hydroboration of such substrates has also been well documented in the literature,^10,11,16,19^ the convenient hydroborative reduction of esters and carbonyl compounds under unrestricted conditions here implies practical applicability of such AM catalysts in synthetic chemistry.

## Conclusions

In summary, we present here for the first time a well-defined potassium catalyst for the selective reduction of esters and carbonyl compounds under mild conditions through hydrosilylation. The catalyst could be readily assembled from 2,2′:6′,2″-terpyridine and potassium triethylborohydride and promoted the hydrosilylative reduction of a range of esters, ketones and aldehydes with good tolerance on reducible functional groups, although other substrates such cyclic esters and ketones are to be extended. Interestingly, a range of β-aryl substituted α,β-unsaturated esters could be selectively reduced on the alkene unit instead of ester group through hydrosilylation. This represents a rare example in alkali metal catalysis. In addition, the AM catalyst proved to be also effective for the reduction of ketones and esters by hydroboration in aqueous media and in the air.

## Author contributions

G. Z. conceived the project. G. Z., P. H., H. M. and M. C. performed experimental studies and analysed the data. G. Z. wrote the manuscript. G. Z. and P. H. revised the manuscript.

## Conflicts of interest

There are no conflicts of interest to declare.

## Supplementary Material

RA-016-D6RA06630E-s001

## Data Availability

The data supporting this article have been included as part of the supplementary information (SI). Supplementary information: general procedures for catalytic reactions, characterisation data and copies of NMR spectra for isolated compounds. Supplementary information is available. See DOI: https://doi.org/10.1039/d6ra06630e.
