# Supplementary material for: Well-defined alkali metal catalysts for selective reduction of functionalized esters and carbonyl compounds via hydrosilylation
Source: RSC Adv. 2026 Oct 5;16(46):60160–5. doi: 10.1039/d6ra06630e (PMC13650931; doi:10.1039/d6ra06630e)
Supplement: Supplementary file 1 [file RA-016-D6RA06630E-s001.pdf]

Supplementary Information (SI) for RSC Advances.
This journal is © The Royal Society of Chemistry

Electronic Supplementary Information:

Well-defined alkali metal catalysts for selective reduction of functionalized esters and carbonyl compounds via hydrosilylation

Guoqi Zhang*, Paulina Hiyaev, Haiyu Mei, Marilyn Chocho

Department of Sciences, John Jay College and PhD in Chemistry Program, The Graduate Center of City University of New York, New York, 10019, New York, USA.

General Considerations. Unless specified otherwise, all reactions were carried out under a dry nitrogen atmosphere using standard glovebox. Anhydrous grade solvents (stored over 4 Å molecular sieves), alkali metal reagents, hydrosilanes, esters and carbonyl compounds were purchased from Sigma-Aldrich, Fisher Scientific or TCI America. ^{1}H NMR and ^{13}C NMR spectra were obtained at room temperature on a Bruker AV 500 or 600 MHz NMR spectrometer, with chemical shifts (δ) referenced to the residual solvent signal. GC-MS analysis was obtained using a Shimadzu GCMS-QP2010S gas chromatograph mass spectrometer.

General Procedure A. In a glovebox under N_2 atmosphere, catalyst **2** (7.4 mg, 10.0 μmol, 1 mol%) was placed in a 20 mL scintillation vial equipped with a stir bar. Ester (1.0 mmol) and THF (1 mL) were added to the vial upon stirring. Phenylsilane (270 mg, 2.5 mmol, 2.5 eq.) was then added dropwise. The reaction mixture was allowed to stir at room temperature for 16 h and then the vial was brought out from the glovebox. MeOH (6 mL) and an aqueous NaOH solution (10% w/w, 4 mL) were added dropwise. Then, the resulting reaction mixture was stirred overnight. The mixture was then extracted with DCM (3 × 10 mL). The combined organic layers were dried over $MgSO_4$, filtered, and the solvents removed under reduced pressure. The organic phase was purified through a column chromatography (silica gel) using ethyl acetate/hexane (1:20, v/v) as an eluent. The pure products of alcohols were obtained and characterized by ^{1}H and ^{13}C NMR spectroscopies.

General Procedure B. In a glovebox under N_2 atmosphere, catalyst **2** (7.4 mg, 10.0 μmol, 1 mol%) was placed in a 20 mL scintillation vial equipped with a stir bar. α, β-Unsaturated esters (**5l-p**, 1.0 mmol) and THF (1 mL) were added to the vial upon stirring. Phenylsilane (270 mg, 2.5 mmol, 2.5 eq.) was then added dropwise. The reaction mixture was allowed to stir at room temperature for 16 h and then the vial was brought out from the glovebox. The reaction was quenched with MeOH (6 mL). After the product was analyzed by GC-MS, the reaction mixture was purified through a flash column chromatography (silica gel) using ethyl acetate/hexane (1:20, v/v) as an eluent. The pure products were characterized by ^{1}H and ^{13}C NMR spectroscopies.

General Procedure C. In a glovebox under N_2 atmosphere, catalyst **2** (7.4 mg, 10.0 μmol, 1 mol%) was placed in a 20 mL scintillation vial equipped with a stir bar. Carbonyl compound (1.0 mmol) and THF (1 mL) were added to the vial upon stirring. Phenylsilane (119 mg, 1.1 mmol, 1.1 eq.) was then added dropwise. The reaction mixture was allowed to stir at room temperature for 1 h and then the vial was brought out from the glovebox. MeOH (6 mL) and an aqueous NaOH solution (10% w/w, 4 mL) were added dropwise. Then, the resulting reaction mixture was stirred overnight. The mixture was then extracted with DCM (3 × 10 mL). The combined organic layers were dried over $MgSO_4$, filtered, and

the solvents removed under reduced pressure. The organic phase was purified through a column chromatography (silica gel) using ethyl acetate/hexane (1:20, v/v) as an eluent. The pure products of alcohols were obtained and characterized by ^{1}H and ^{13}C NMR spectroscopies.

General Procedure for Ketone Reduction in Aqueous Media. In a glovebox under N_2 atmosphere, catalyst **2** (10.0 μmol, 1 mol%) was placed in a 20 mL scintillation vial equipped with a stir bar. Acetophenone (1.0 mmol) and THF (0.9 mL) were added to the vial upon stirring. The vial was then brought out of the glovebox and exposed to the air. Distilled water (0.1 mL) was added, followed by pinacolborane (1.1 mmol, 1.1 eq.) or phenylsilane (1.1 mmol, 1.1 eq.) that was added dropwise. The reaction mixture was allowed to stir at room temperature for 2 h in the air. The reaction was then quenched with MeOH and then analyzed by GC-MS.

General Procedure for Ester Reduction in Aqueous Media. In a glovebox under N_2 atmosphere, catalyst **2** (10.0 μmol, 1 mol%) was placed in a 20 mL scintillation vial equipped with a stir bar. Methyl benzoate (1.0 mmol) and THF (0.9 mL) were added to the vial upon stirring. The vial was then brought out of the glovebox and exposed to the air. Distilled water (0.1 mL) was added, followed by pinacolborane (2.5 mmol, 2.5 eq.) or phenylsilane (2.5 mmol, 2.5 eq.) that was added dropwise. The reaction mixture was allowed to stir at room temperature for 16 h in the air. The reaction was then quenched with MeOH and then analyzed by GC-MS.

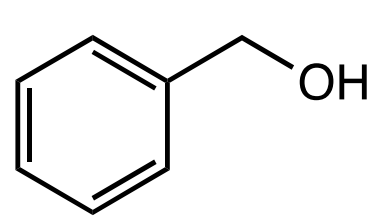

Benzylic alcohol (6a):[1] The synthesis of **6a** followed the general procedure A. Eluents for column chromatography on silica gel: ethyl acetate/hexane (1:20, v/v) Colorless oil. Yield: 96 mg (89%). ^{1}H NMR (400 MHz, $CDCl_3$) δ 7.27 – 7.15 (m, 5H), 4.50 (s, 2H), 2.47 (s, 1H) ppm; ^{13}C NMR (101 MHz, $CDCl_3$) δ 141.0, 128.8(2C), 127.9, 127.3(2C), 65.4 ppm.

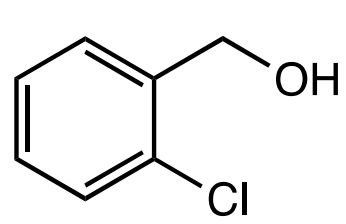

2-Chlorobenzylic alcohol (6b):[2] The synthesis of **6b** followed the general procedure A. Eluents for column chromatography on silica gel: ethyl acetate/hexane (1:20, v/v) Colorless oil. Yield: 128 mg (90%). ^{1}H NMR (500 MHz, $CDCl_3$) δ 7.48 (dd, *J* = 7.4, 1.9 Hz, 1H), 7.36 (dd, *J* = 7.7, 1.5 Hz, 1H), 7.31 – 7.20 (m, 2H), 4.78 (s, 2H), 2.12 (s, 1H) ppm; ^{13}C NMR (126 MHz, $CDCl_3$) δ 138.1, 132.7, 129.3, 128.8, 128.7, 127.0, 62.9 ppm.

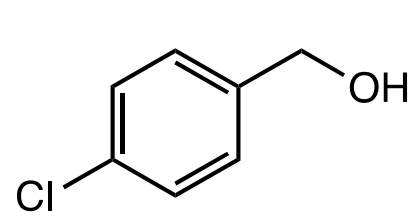

4-Chlorobenzylic alcohol (6c):[2] The synthesis of **6c** followed the general procedure A. Eluents for column chromatography on silica gel: ethyl acetate/hexane (1:20, v/v). Colorless oil. Yield: 132 mg (93%). ^{1}H NMR (600 MHz, $CDCl_3$) δ 7.20 (d, *J* = 8.5 Hz, 2H), 7.14 (d, *J* = 8.5 Hz, 2H), 4.48 (s, 2H), 2.58 (s, 1H) ppm; ^{13}C NMR (151 MHz, $CDCl_3$) δ 139.2, 133.3, 128.6(2C), 128.3(2C), 64.3 ppm.

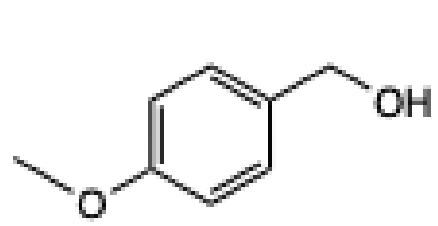

4-Methoxybenzylic alcohol (6d):[2] The synthesis of **6d** followed the general procedure A. Eluents for column chromatography on silica gel: ethyl

acetate/hexane (1:20, v/v). Colorless oil. Yield: 119 mg (86%). ^{1}H NMR (500 MHz, CDCl$_3$) δ 7.25 (d, *J* = 8.6 Hz, 2H), 6.88 (d, *J* = 8.6 Hz, 2H), 4.54 (s, 2H), 3.79 (s, 3H), 3.10 (s, 1H) ppm; ^{13}C NMR (151 MHz, CDCl$_3$) δ 159.2, 133.4, 128.8(2C), 114.0(2C), 64.8, 55.4 ppm.

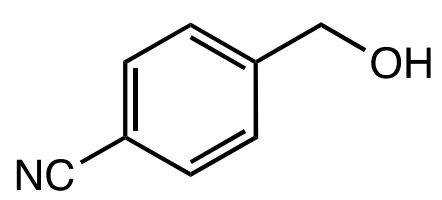

4-Cyanobenzylic alcohol (6e):[2] The synthesis of **6e** followed the general procedure A. Eluents for column chromatography on silica gel: ethyl acetate/hexane (1:20, v/v). Colorless oil. Yield: 109 mg (82%). ^{1}H NMR (500 MHz, CDCl$_3$) δ 7.57 (d, *J* = 7.9 Hz, 2H), 7.40 (d, *J* = 7.9 Hz, 2H), 4.71 (s, 2H), 2.13 (s, 1H) ppm; ^{13}C NMR (126 MHz, CDCl$_3$) δ 146.3, 132.3(2C), 127.0(2C), 118.9, 111.1, 64.2 ppm.

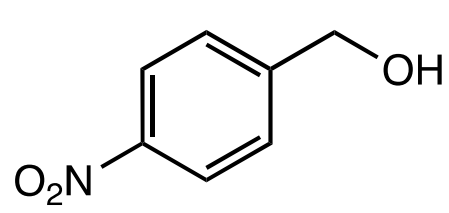

4-Nitrobenzylic alcohol (6f):[1] The synthesis of **6f** followed the general procedure A. Eluents for column chromatography on silica gel: ethyl acetate/hexane (1:15, v/v). Pale yellow oil. Yield: 121 mg (79%). ^{1}H NMR (600 MHz, CDCl$_3$) δ 8.17 (d, *J* = 8.8 Hz, 2H), 7.51 (d, *J* = 9.0 Hz, 2H), 4.81 (s, 2H), 2.31 (s, 1H) ppm; ^{13}C NMR (151 MHz, CDCl$_3$) δ 148.4, 147.4, 127.1(2C), 123.8(2C), 64.0 ppm.

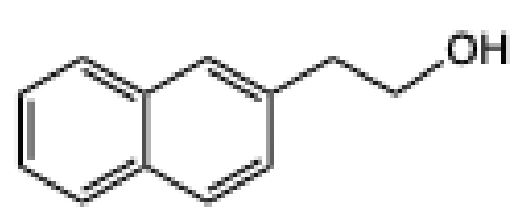

2-(Naphthalen-2-yl)ethan-1-ol (6g):[3] The synthesis of **6g** followed the general procedure A. Eluents for column chromatography on silica gel: ethyl acetate/hexane (1:20, v/v). Colorless oil. Yield: 155 mg (90%). ^{1}H NMR (500 MHz, CDCl$_3$) δ 8.10 – 8.03 (m, 1H), 7.87 (d, *J* = 7.8 Hz, 1H), 7.76 (d, *J* = 8.1 Hz, 1H), 7.57 – 7.46 (m, 2H), 7.44 – 7.40 (m, 1H), 7.38 (d, *J* = 6.8 Hz, 1H), 3.99 (t, *J* = 6.7 Hz, 2H), 3.35 (t, *J* = 6.6 Hz, 2H), 1.48 (s, 1H) ppm; ^{13}C NMR (126 MHz, CDCl$_3$) δ 134.31, 133.95, 132.01, 128.85, 127.33, 127.18, 125.66, 125.49, 123.63, 63.02, 36.20 ppm.

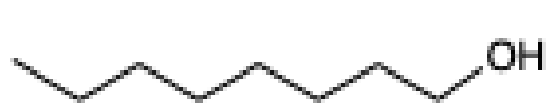

1-Octanol (6h):[2] The synthesis of **6h** followed the general procedure A. Eluents for column chromatography on silica gel: ethyl acetate/hexane (1:40, v/v). Colorless oil. Yield: 104 mg (80%). ^{1}H NMR (600 MHz, CDCl$_3$) δ 3.68 – 3.49 (m, 2H), 2.01 (s, 1H), 1.53 (dt, *J* = 8.4, 6.6 Hz, 2H), 1.46 – 1.03 (m, 10H), 0.93 – 0.76 (m, 3H) ppm; ^{13}C NMR (151 MHz, CDCl$_3$) δ 63.2, 33.0, 32.1, 29.7, 29.6, 26.1, 22.9, 14.4 ppm.

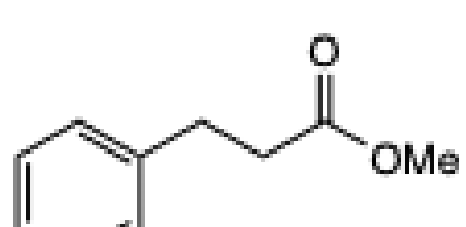

Methyl 3-phenylpropanoate (6k):[4] The synthesis of **6k** followed the general procedure B. Eluents for column chromatography on silica gel: ethyl acetate/hexane (1:40, v/v). Colorless oil. Yield: 104 mg (80%). ^{1}H NMR (500 MHz, CDCl$_3$) δ 7.57 (d, *J* = 7.9 Hz, 2H), 7.40 (d, *J* = 7.9 Hz, 2H), 4.71 (s, 2H), 2.13 (s, 1H) ppm; ^{13}C NMR (101 MHz, CDCl$_3$) δ 173.7, 140.7, 128.8(2C), 128.6(2C), 126.5, 52.0, 36.0, 31.1 ppm. GC-MS: 164.

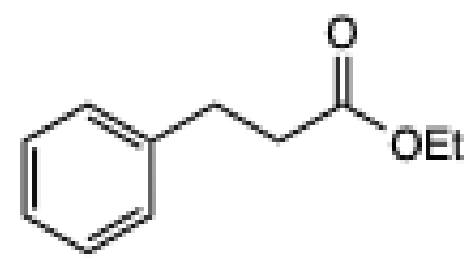

Ethyl 3-phenylpropanoate (6l):[4] The synthesis of **6l** followed the general procedure B. Eluents for column chromatography on silica gel: ethyl acetate/hexane (1:40, v/v). Colorless oil. Yield: 104 mg (80%). ^{1}H NMR (400 MHz, $CDCl_3$) δ 7.32 – 7.23 (m, 2H), 7.23 – 7.13 (m, 3H), 4.12 (q, *J* = 7.1 Hz, 2H), 2.99 – 2.88 (m, 2H), 2.66 – 2.57 (m, 2H), 1.23 (t, *J* = 7.2 Hz, 3H). GC-MS: 178.

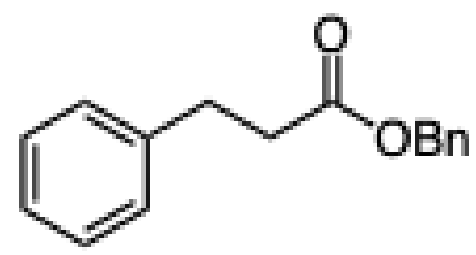

Benzyl 3-phenylpropanoate (6m):[4] The synthesis of **6m** followed the general procedure B. Eluents for column chromatography on silica gel: ethyl acetate/hexane (1:40, v/v). Colorless oil. Yield: 104 mg (80%). ^{1}H NMR (600 MHz, $CDCl_3$) δ 7.37 – 7.28 (m, 5H), 7.26 (d, *J* = 1.7 Hz, 2H), 7.22 – 7.16 (m, 3H), 5.11 (s, 2H), 2.97 (t, *J* = 7.8 Hz, 2H), 2.69 (t, *J* = 7.8 Hz, 2H) ppm; ^{13}C NMR (151 MHz, $CDCl_3$) δ 172.7, 140.5, 136.0, 128.57, 128.53, 128.3, 128.2, 126.3, 66.3, 35.9, 31.0 ppm. GC-MS: 240.

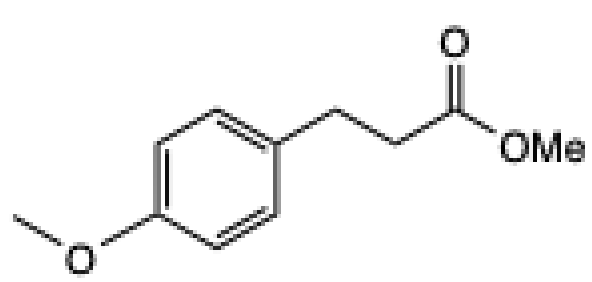

Methyl 3-(4-methoxyphenyl)propanoate (6n):[5] The synthesis of **6n** followed the general procedure B. Eluents for column chromatography on silica gel: ethyl acetate/hexane (1:40, v/v). Colorless oil. Yield: 104 mg (80%). ^{1}H NMR (400 MHz, $CDCl_3$) δ 7.17 – 7.08 (m, 2H), 6.83 (d, *J* = 8.6 Hz, 2H), 4.12 (q, *J* = 7.1 Hz, 2H), 3.78 (s, 3H), 2.89 (t, *J* = 7.8 Hz, 2H), 2.58 (dd, *J* = 8.4, 7.2 Hz, 2H), 1.23 (t, *J* = 7.1 Hz, 3H) ppm; ^{13}C NMR (101 MHz, $CDCl_3$) δ 173.3, 158.4, 133.0, 129.6(2C), 114.2(2C), 60.7, 55.6, 36.6, 30.5, 14.6 ppm. GC-MS: 194.

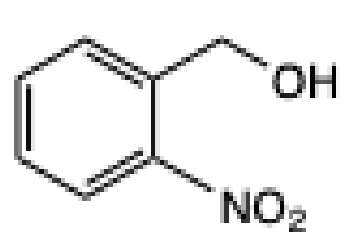

2-Nitrobenzylic alcohol (9a):[6] The synthesis of **9a** followed the general procedure C. Eluents for column chromatography on silica gel: ethyl acetate/hexane (1:30, v/v). Pale yellow oil. Yield: 133 mg (95%). ^{1}H NMR (500 MHz, $CDCl_3$) δ 8.04 (dd, *J* = 8.1, 1.3 Hz, 1H), 7.71 (dd, *J* = 7.8, 1.4 Hz, 1H), 7.62 (td, *J* = 7.5, 1.3 Hz, 1H), 7.45 – 7.40 (m, 1H), 4.92 (s, 2H), 3.10 (br., 1H) ppm; ^{13}C NMR (126 MHz, $CDCl_3$) δ 147.4, 136.9, 134.1, 129.6, 128.4, 124.9, 62.3 ppm.

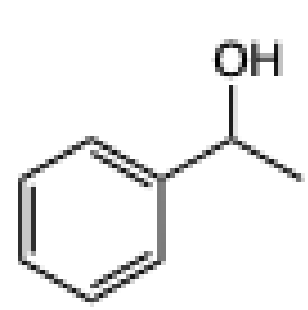

1-Phenylethanol (9b):[2] The synthesis of **9b** followed the general procedure C. Eluents for column chromatography on silica gel: ethyl acetate/hexane (1:40, v/v). Colorless oil. Yield: 112 mg (92%). ^{1}H NMR (400 MHz, $CDCl_3$) δ 7.46 – 7.35 (m, 4H), 7.31 (ddd, *J* = 6.0, 4.7, 2.6 Hz, 1H), 4.90 (q, *J* = 6.5 Hz, 1H), 1.52 (d, *J* = 6.5 Hz, 3H) ppm; ^{13}C NMR (101 MHz, $CDCl_3$) δ 146.1, 128.8(2C), 127.7, 125.7(2C), 70.6, 25.4 ppm.

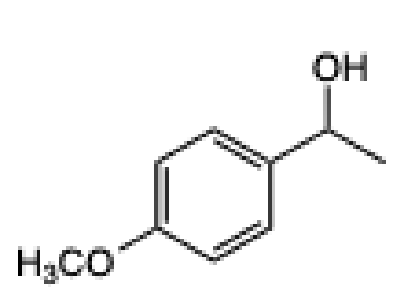

1-(4-Methoxyphenyl)ethanol (9c):[2] The synthesis of **9c** followed the general procedure C. Eluents for column chromatography on silica gel: ethyl acetate/hexane (1:40, v/v). Colorless oil. Yield: 137 mg (90%). ^{1}H NMR (600 MHz, $CDCl_3$) δ 7.19 (d, *J* = 8.8 Hz, 2H), 6.78 (d, *J* = 8.8 Hz, 2H), 4.74 (q, *J* = 6.5 Hz, 1H), 3.70 (s, 3H), 2.08 (s, 1H), 1.37 (d,

J = 6.6 Hz, 3H) ppm; ^{13}C NMR (151 MHz, $CDCl_3$) δ 159.0, 138.1, 126.7(2C), 113.8(2C), 69.9, 55.3, 25.0 ppm.

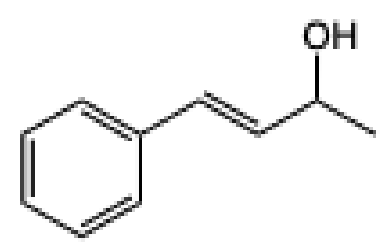

(*E*)-4-phenylbut-3-en-2-ol (9d):[1] The synthesis of **9d** followed the general procedure C. Eluents for column chromatography on silica gel: ethyl acetate/hexane (1:40, v/v). Colorless oil. Yield: 121 mg (93%). ^{1}H NMR (500 MHz, $CDCl_3$) δ 7.42 – 7.34 (m, 2H), 7.34 – 7.28 (m, 2H), 7.27 – 7.20 (m, 1H), 6.55 (dd, J = 15.9, 1.2 Hz, 1H), 6.25 (dd, J = 15.9, 6.4 Hz, 1H), 4.52 – 4.43 (m, 1H), 1.90 (s, 1H), 1.36 (d, J = 6.4 Hz, 3H) ppm; ^{13}C NMR (151 MHz, $CDCl_3$) δ 137.1, 133.9, 129.7, 128.9(2C), 127.9, 126.8(2C), 69.2, 23.7 ppm.

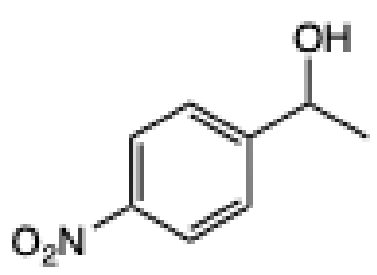

1-(4-Nitrophenyl)ethanol (9e):[1] The synthesis of **9e** followed the general procedure C. Eluents for column chromatography on silica gel: ethyl acetate/hexane (1:30, v/v). Pale yellow oil. Yield: 142 mg (85%). ^{1}H NMR (600 MHz, $CDCl_3$) δ 8.12 (d, J = 8.8 Hz, 2H), 7.50 (dd, J = 9.0, 0.8 Hz, 2H), 4.97 (q, J = 6.5 Hz, 1H), 2.82 (s, 1H) ppm; ^{13}C NMR (126 MHz, $CDCl_3$) δ 153.3, 147.0, 126.2(2C), 123.7(2C), 69.4, 25.4 ppm.

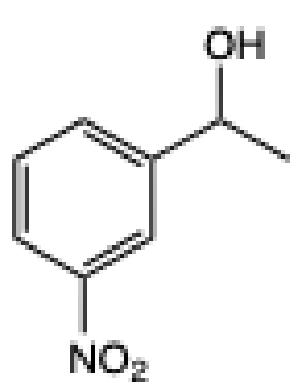

1-(3-Nitrophenyl)ethanol (9f):[7] The synthesis of **9f** followed the general procedure C. Eluents for column chromatography on silica gel: ethyl acetate/hexane (1:30, v/v). Pale yellow oil. Yield: 121 mg (82%). ^{1}H NMR (600 MHz, $CDCl_3$) δ 7.11 (t, J = 7.7 Hz, 1H), 6.76 – 6.70 (m, 1H), 6.68 (t, J = 2.0 Hz, 1H), 6.57 (ddd, J = 7.9, 2.4, 1.0 Hz, 1H), 4.77 (q, J = 6.5 Hz, 1H), 3.34 (s, 1H), 1.44 (d, J = 6.5 Hz, 3H) ppm.

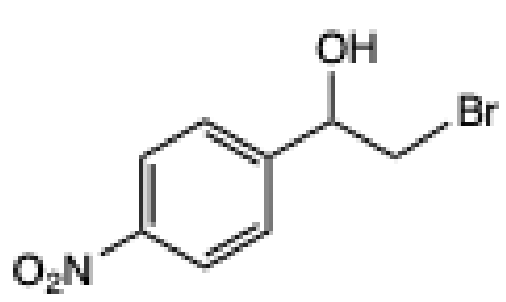

2-Bromo-1-(4-nitrophenyl)ethan-1-ol (9g):[8] The synthesis of **9g** followed the general procedure C. Eluents for column chromatography on silica gel: ethyl acetate/hexane (1:20, v/v). Pale yellow oil. Yield: 121 mg (81%). ^{1}H NMR (500 MHz, $CDCl_3$) δ 8.23 (d, J = 8.8 Hz, 2H), 7.58 (d, J = 8.7 Hz, 2H), 5.04 (dd, J = 8.4, 3.5 Hz, 1H), 3.67 (dd, J = 10.7, 3.5 Hz, 1H), 3.52 (dd, J = 10.6, 8.4 Hz, 1H), 2.78 (s, 1H) ppm; ^{13}C NMR (126 MHz, $CDCl_3$) δ 147.9, 147.2, 127.0(2C), 123.9(2C), 72.7, 39.4 ppm.

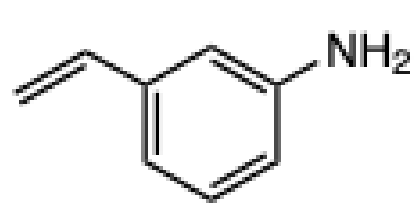

3-Vinylaniline (10):[9] In a glovebox under N_2 atmosphere, catalyst **2** (7.4 mg, 10.0 μmol, 1 mol%) was placed in a 20 mL scintillation vial equipped with a stir bar. 3-Nitrostyrene (149 mg, 1.0 mmol) and THF (1 mL) were added to the vial upon stirring. Phenylsilane (162 mg, 1.5 mmol, 1.5 eq.) was then added dropwise. The reaction mixture was allowed to stir at 60 °C for 16 h and then the vial was brought out from the glovebox. MeOH (6 mL) and an aqueous NaOH solution (10% w/w, 4 mL) were added dropwise. Then, the resulting reaction mixture was stirred overnight. The mixture was then extracted with DCM (3 × 10 mL). The combined organic layers were dried over $MgSO_4$, filtered, and the solvents removed under reduced pressure. The organic phase was purified through a column chromatography (silica gel) using ethyl acetate/hexane (1:20, v/v) as an eluent to give yellowish oil. Yield: 101 mg (85%). ^{1}H NMR (600 MHz, $CDCl_3$) δ

7.13 (t, *J* = 7.8 Hz, 1H), 6.84 (d, *J* = 7.6 Hz, 1H), 6.80 – 6.71 (m, 1H), 6.65 (dd, *J* = 17.6, 10.9 Hz, 1H), 6.60 (dd, *J* = 8.0, 2.5 Hz, 1H), 5.71 (d, *J* = 17.6 Hz, 1H), 5.22 (d, *J* = 10.9 Hz, 1H), 3.65 (s, 2H) ppm.
^{13}C NMR (126 MHz, $CDCl_3$) δ 146.6, 138.7, 137.0, 129.5, 117.0, 114.8, 113.7, 112.7 ppm.

^{1}H and ^{13}C NMR spectra for isolated products.

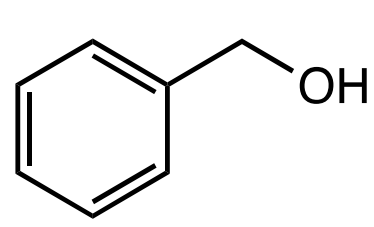

6a

^{1}H NMR spectrum (400 MHz, $CDCl_3$; solvent residue found at 1.1-1.2 ppm):

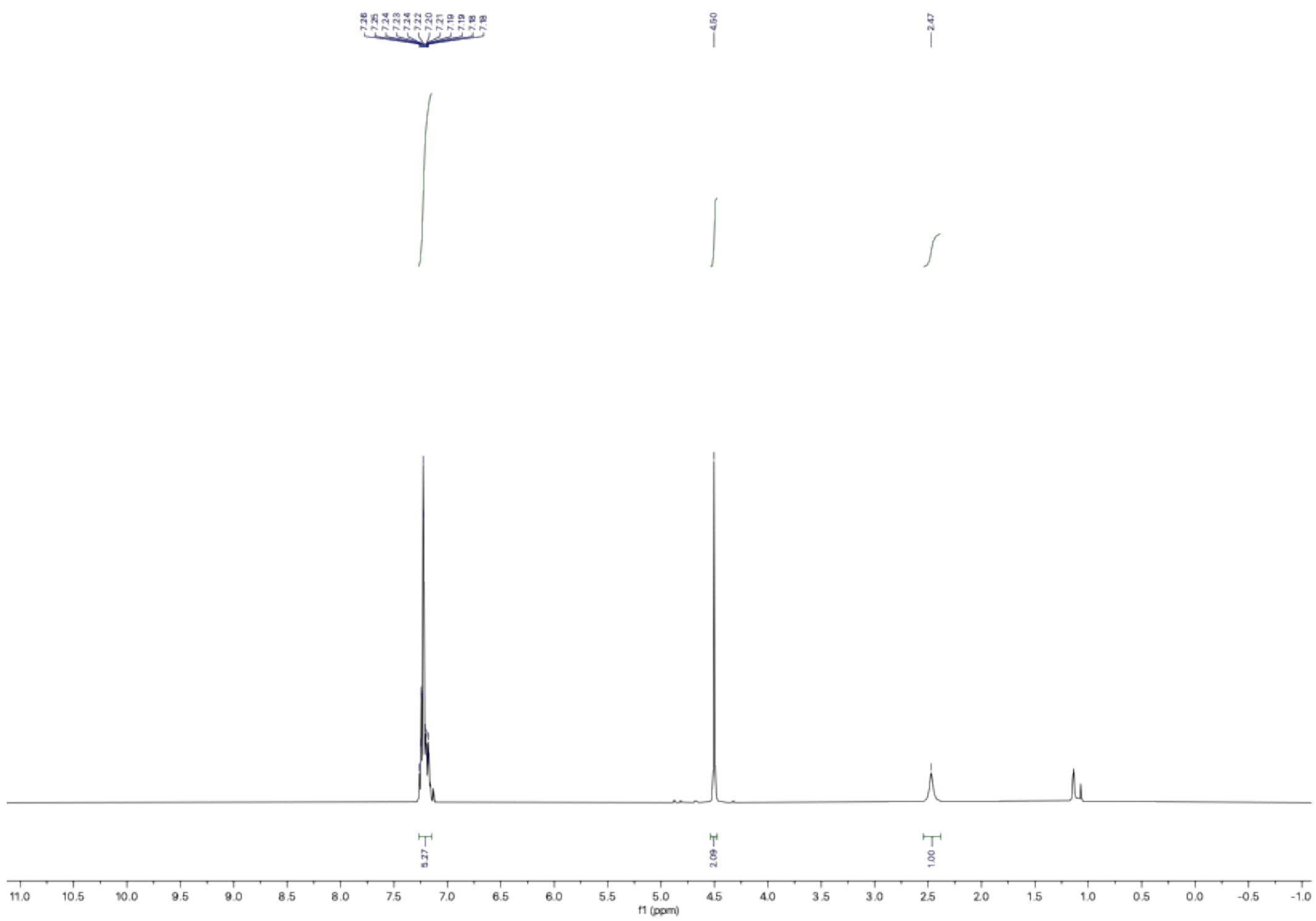

^{13}C NMR spectrum (101 MHz, $CDCl_3$):

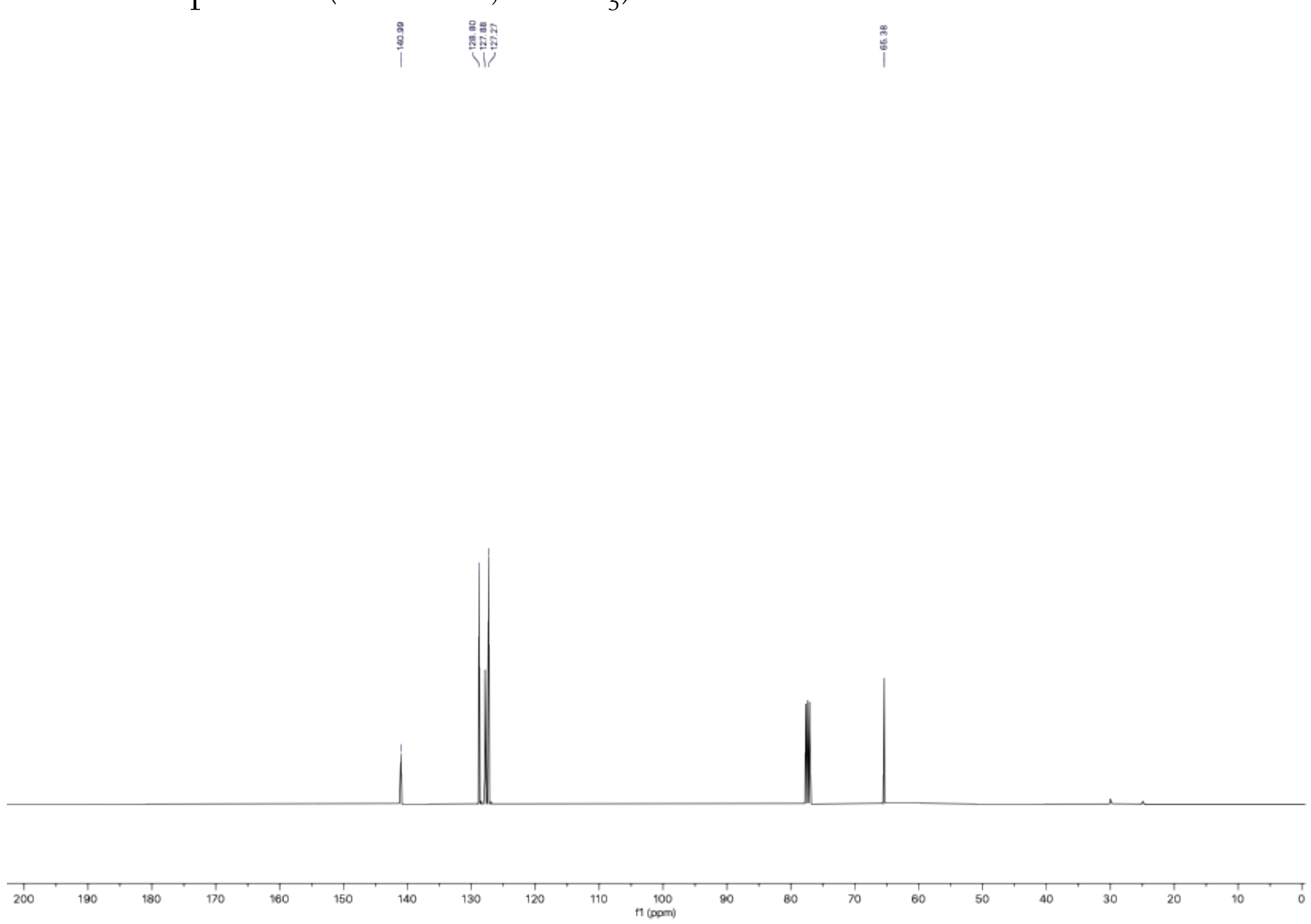

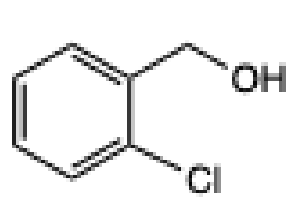

6b

^{1}H NMR spectrum (500 MHz, $CDCl_3$):

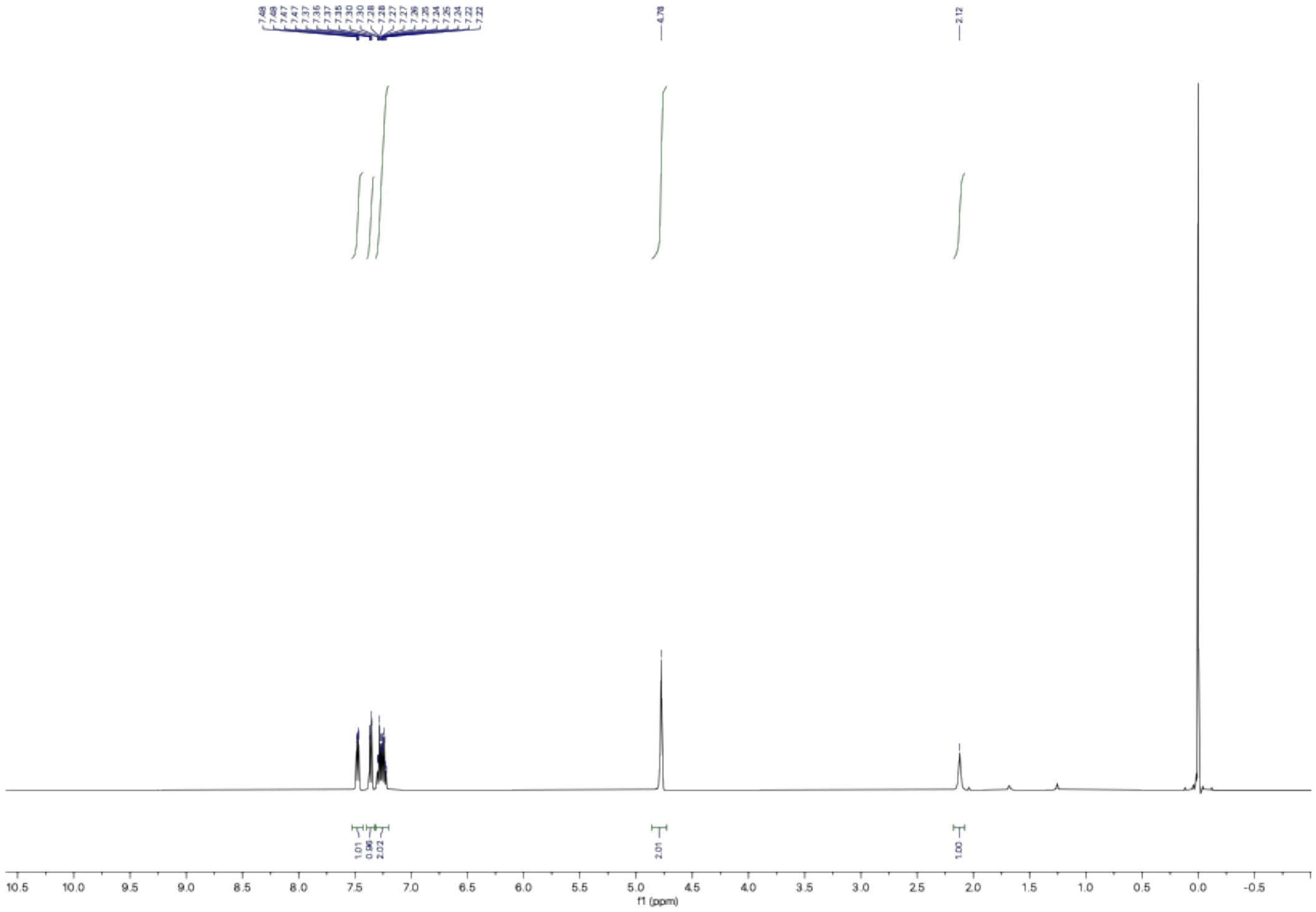

^{13}C NMR spectrum (126 MHz, $CDCl_3$):

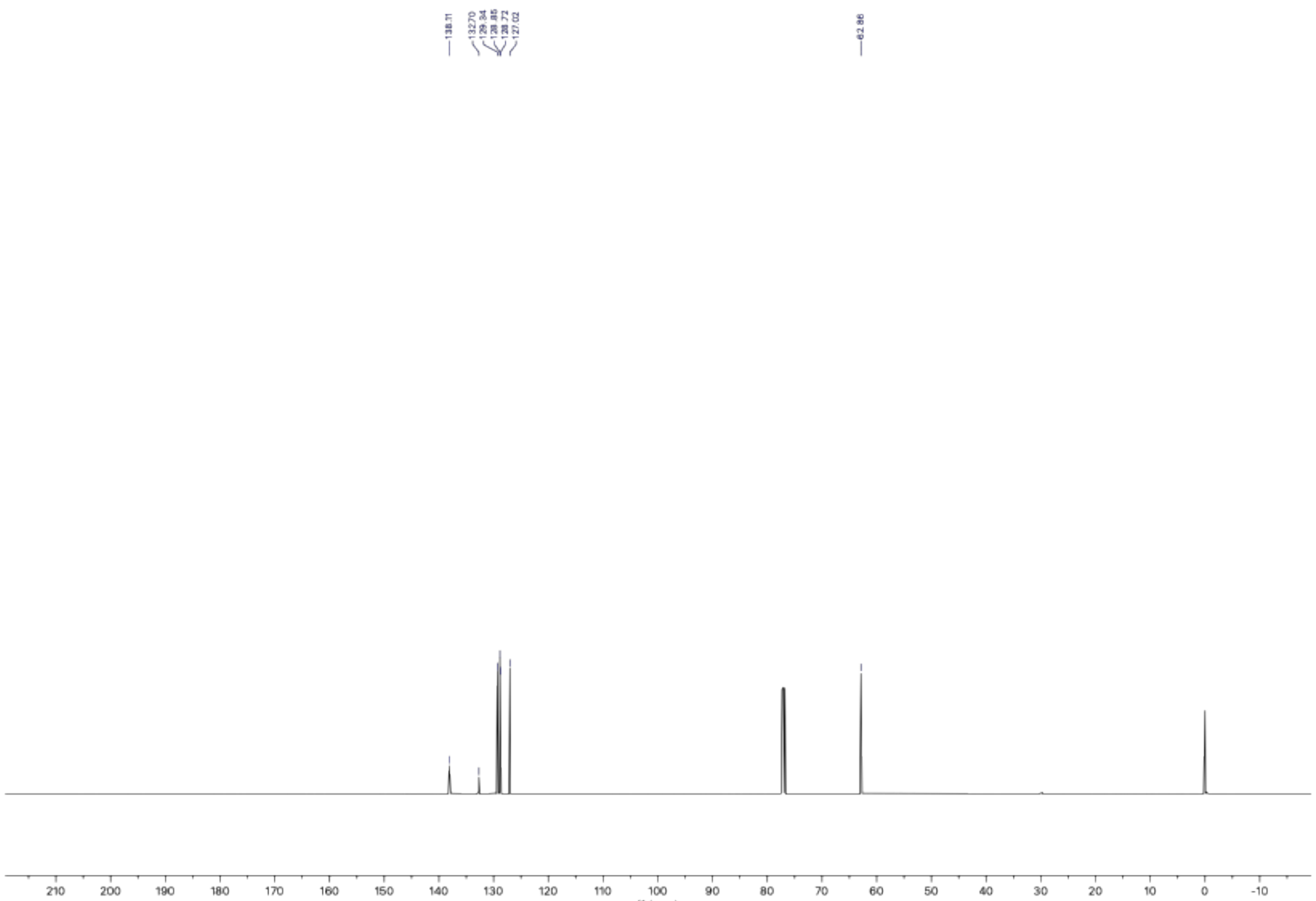

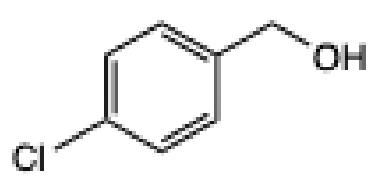

6c

^{1}H NMR spectrum (600 MHz, $CDCl_3$):

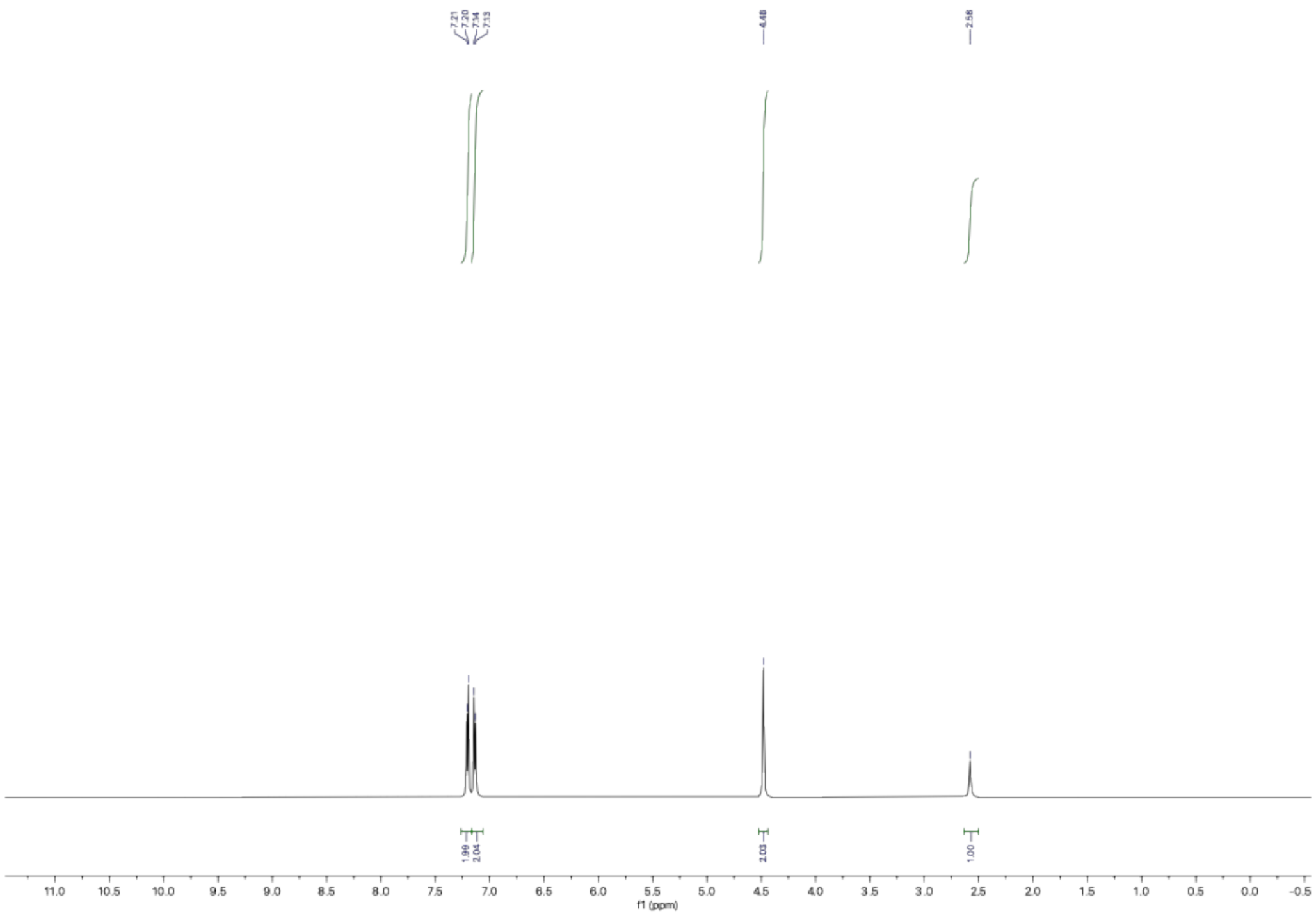

^{13}C NMR spectrum (151 MHz, $CDCl_3$):

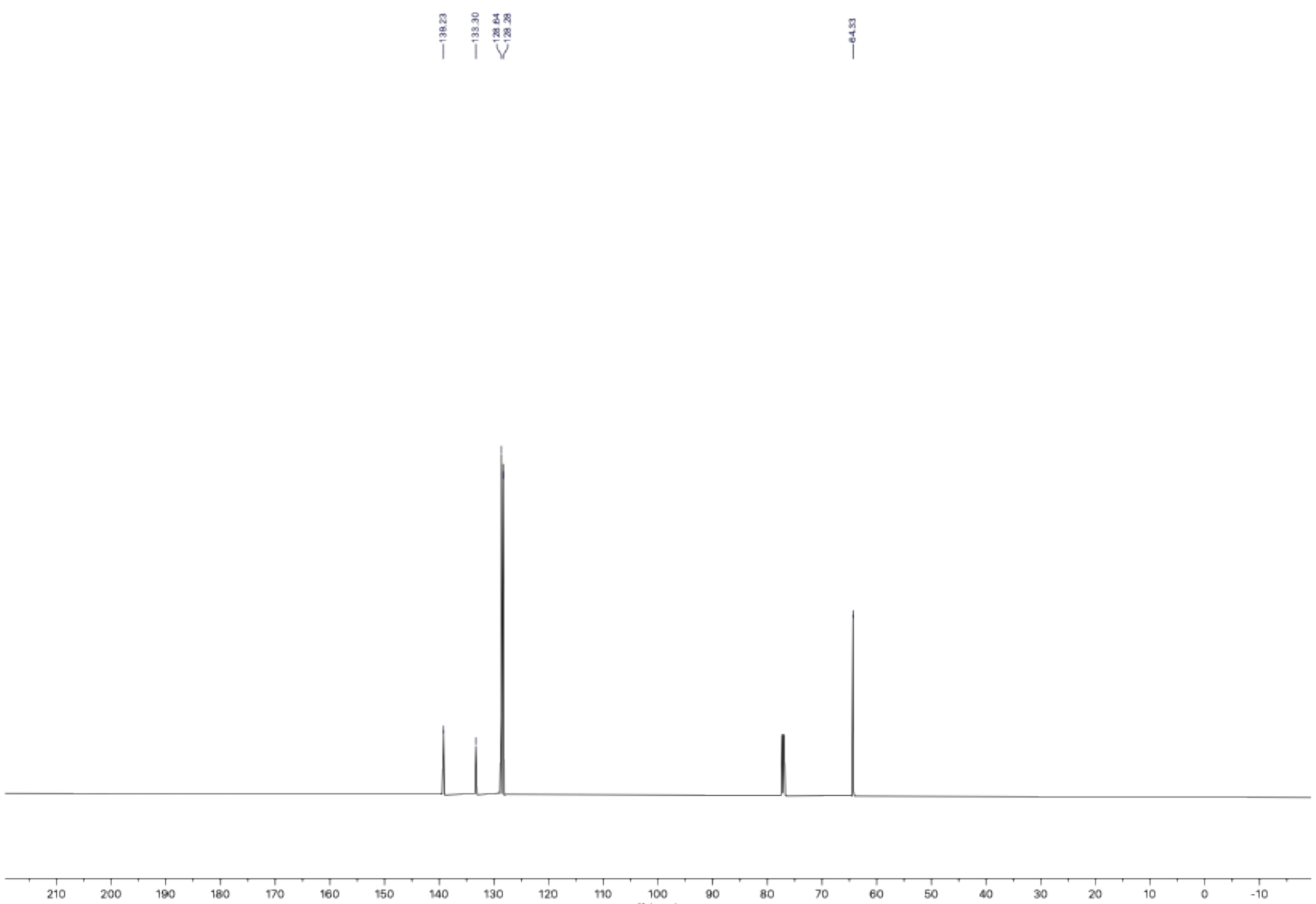

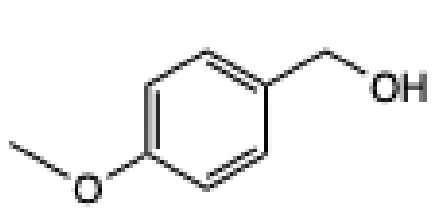

6d

^1H NMR spectrum (500 MHz, $CDCl_3$; solvent residue found at 1.1-1.3 ppm):

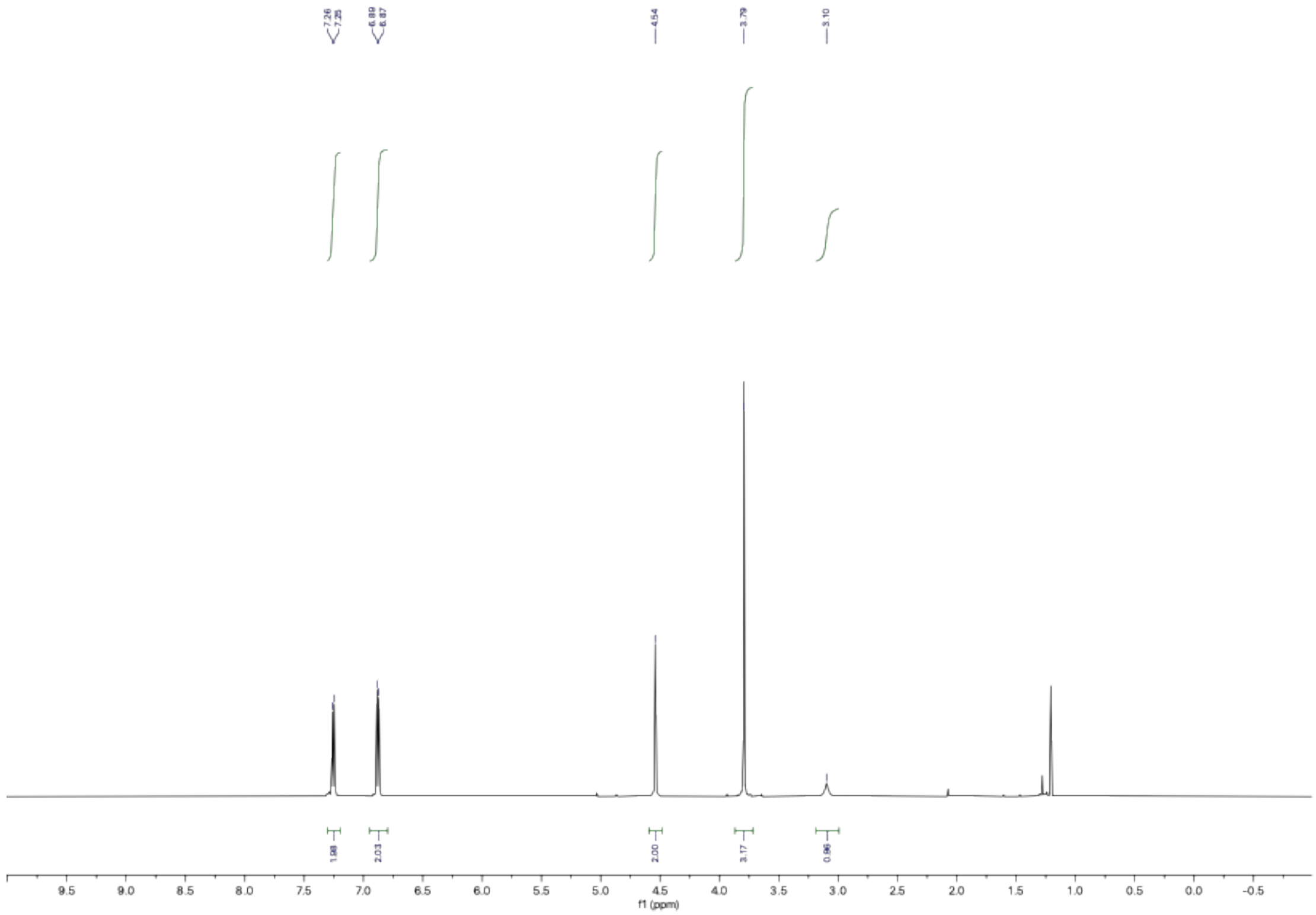

^{13}C NMR spectrum (151 MHz, $CDCl_3$):

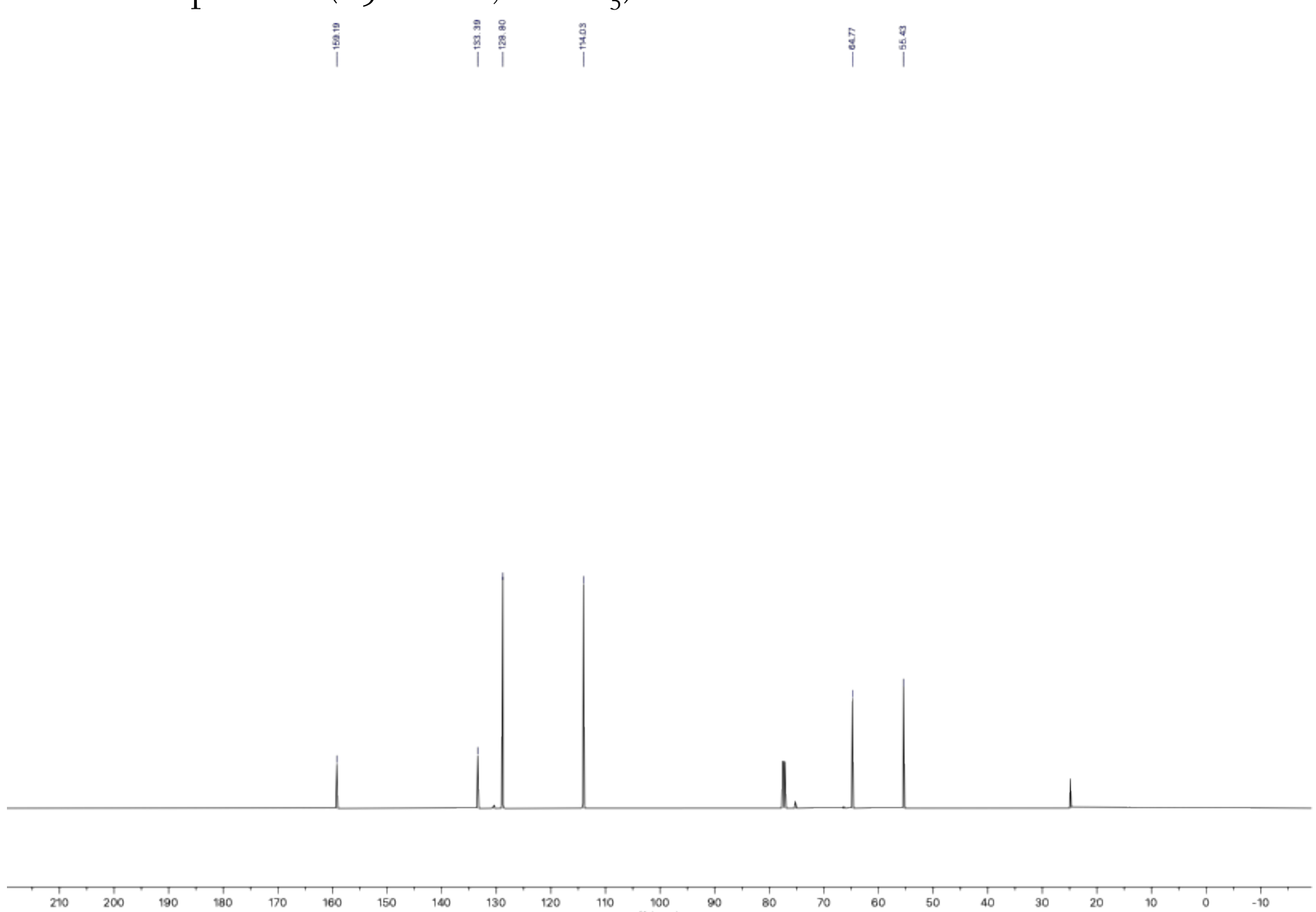

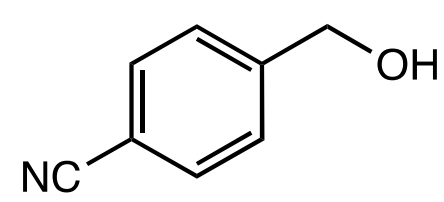

6e

^{1}H NMR spectrum (500 MHz, $CDCl_3$):

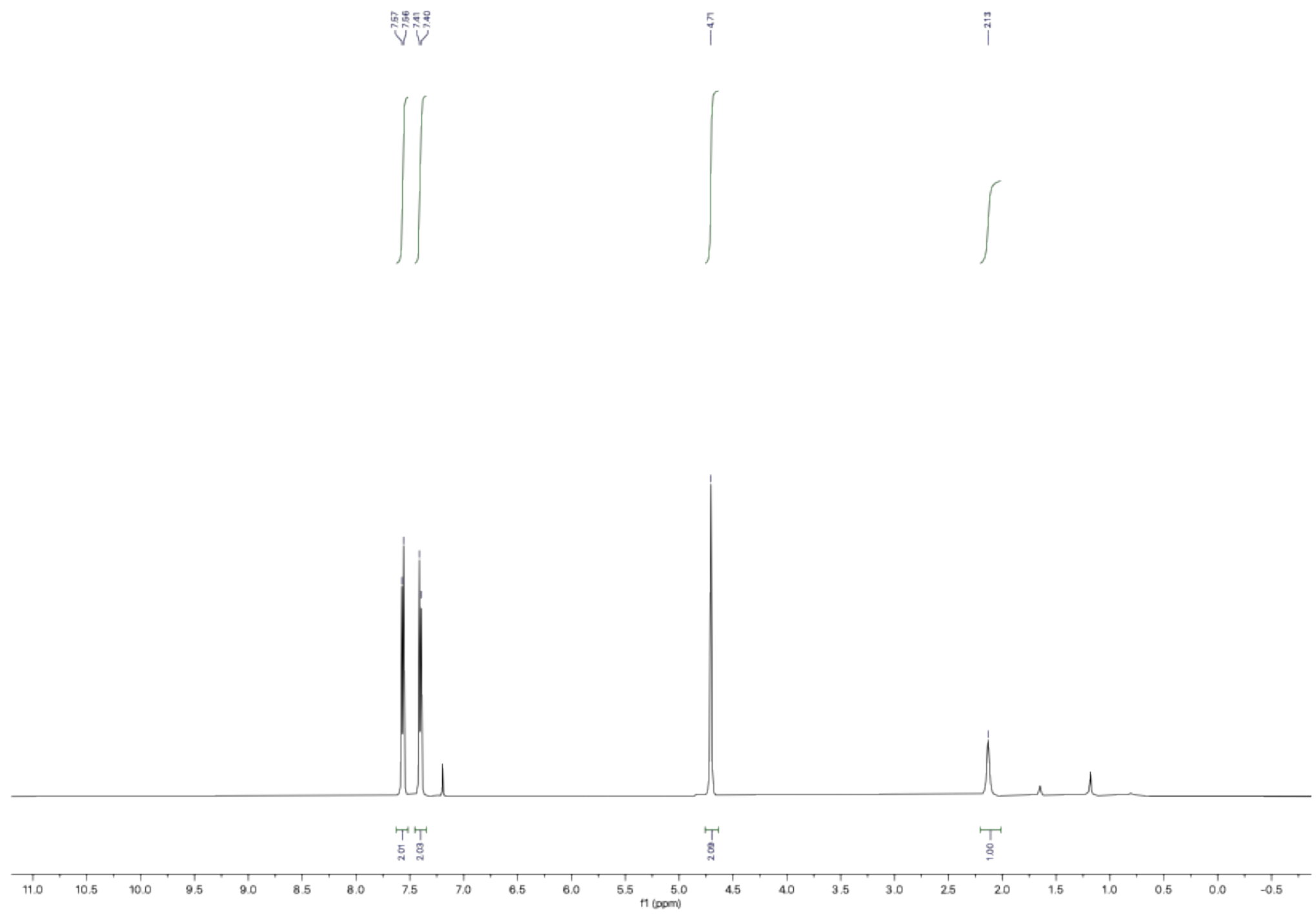

^{13}C NMR spectrum (126 MHz, $CDCl_3$):

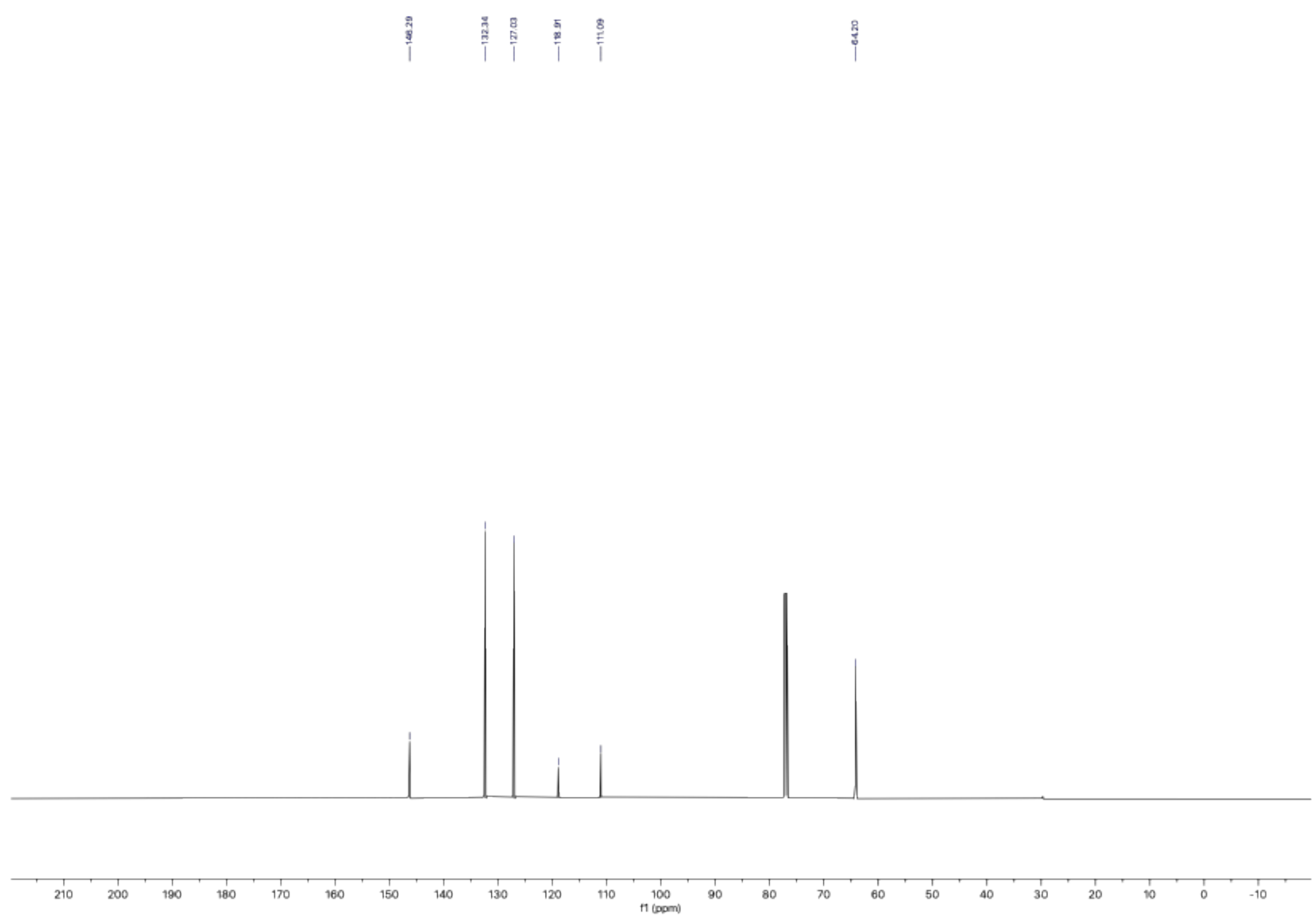

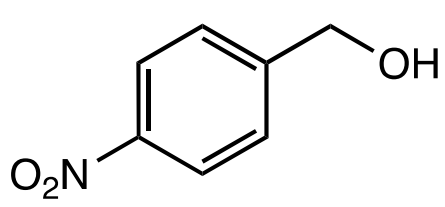

6f

^{1}H NMR spectrum (600 MHz, $CDCl_3$):

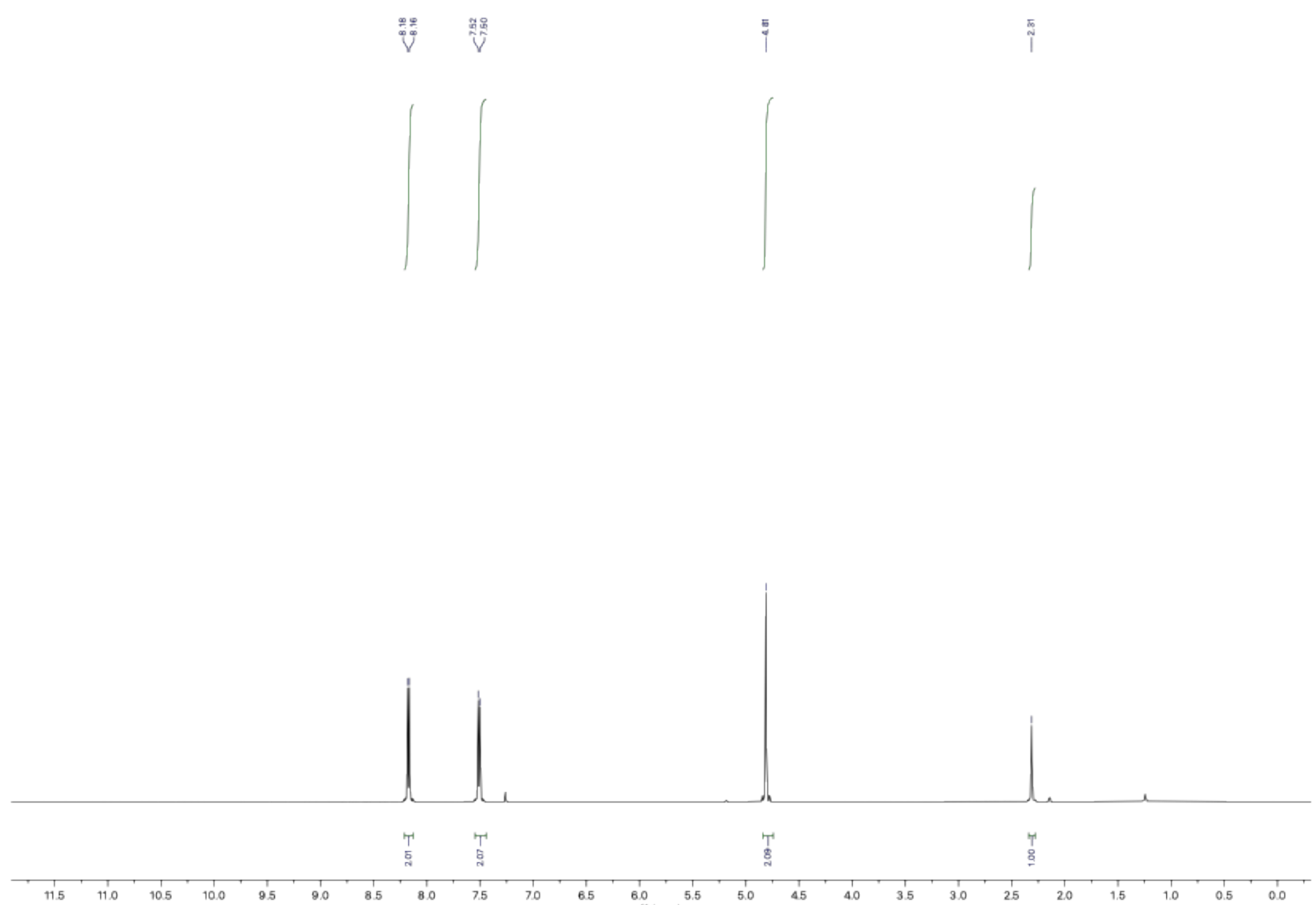

^{13}C NMR spectrum (151 MHz, $CDCl_3$):

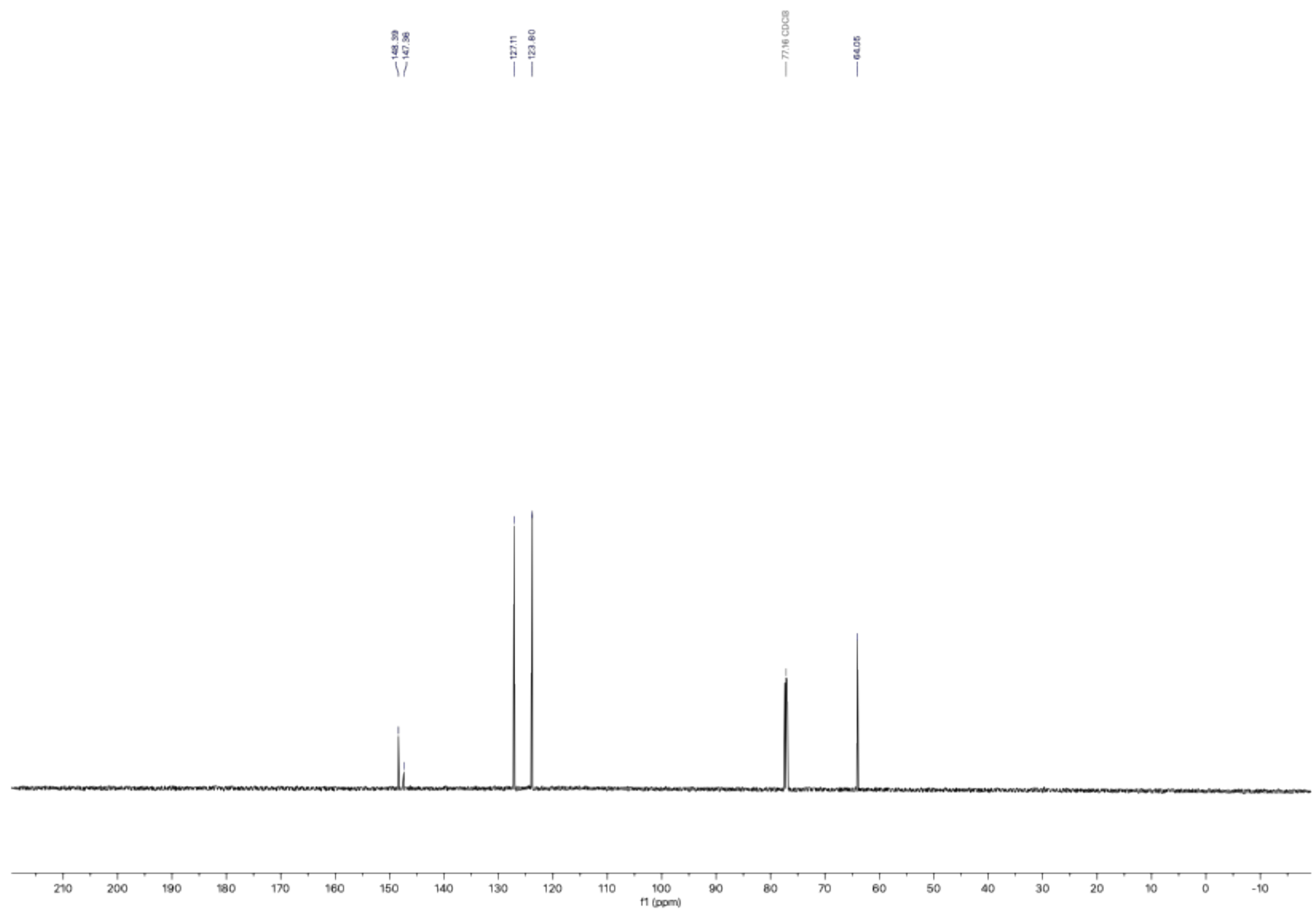

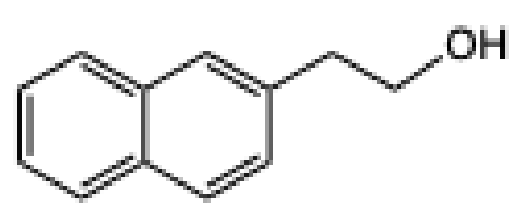

6g

^{1}H NMR spectrum (500 MHz, $CDCl_3$):

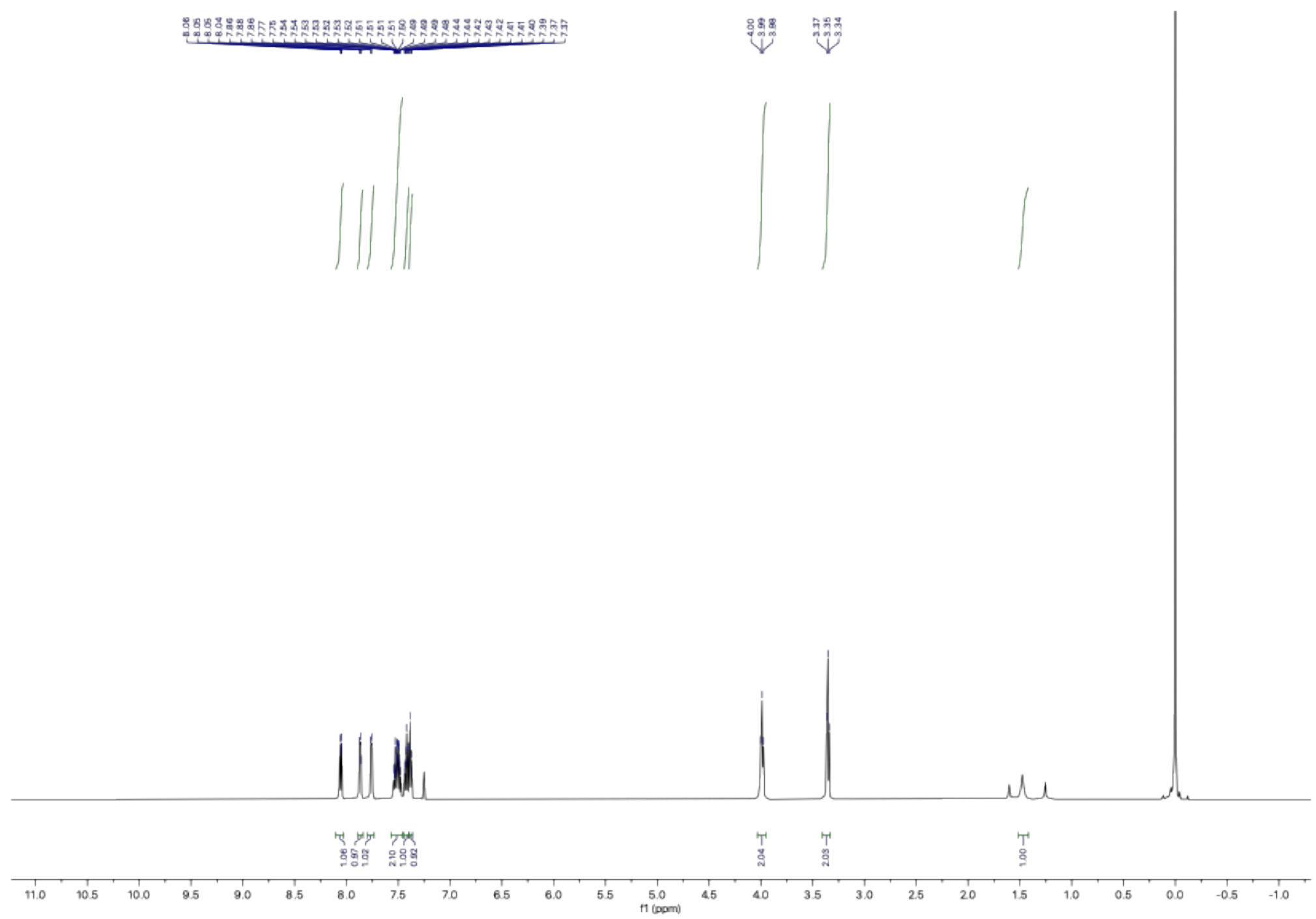

^{13}C NMR spectrum (126 MHz, $CDCl_3$):

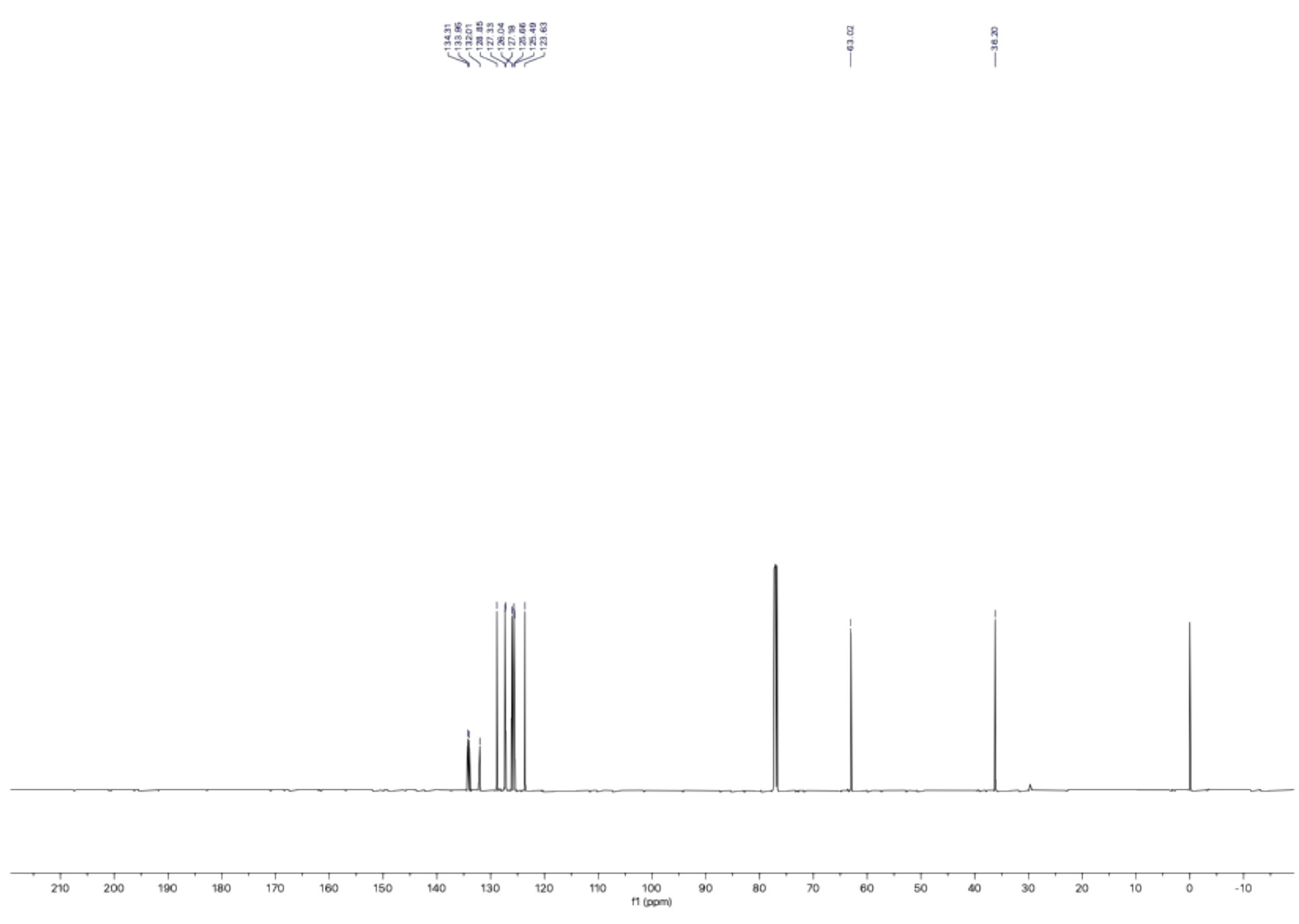

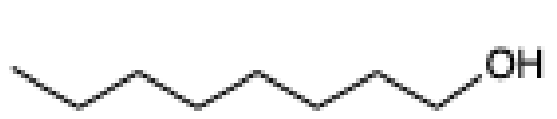

6h

^{1}H NMR spectrum (600 MHz, $CDCl_3$):

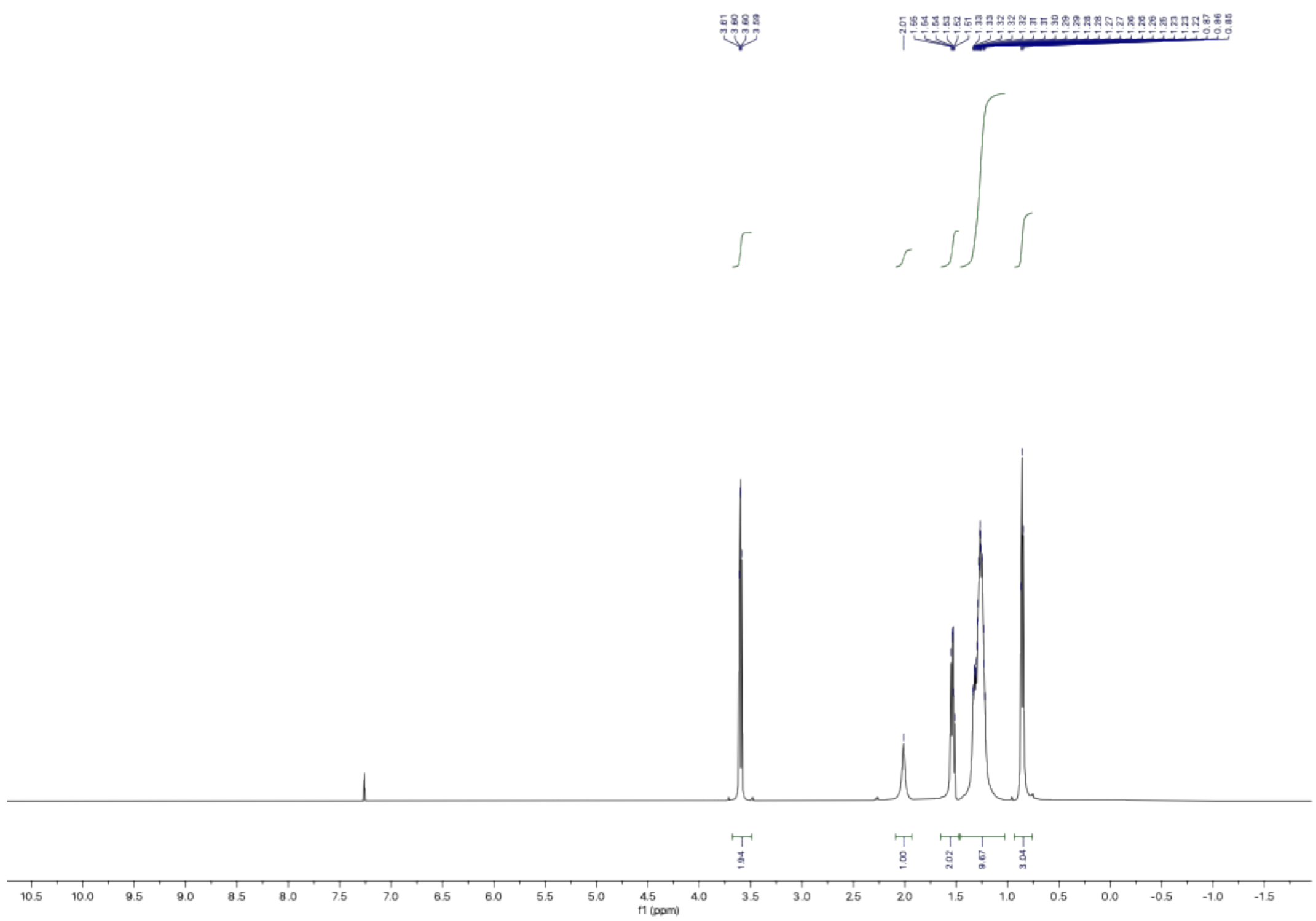

^{13}C NMR spectrum (151 MHz, $CDCl_3$):

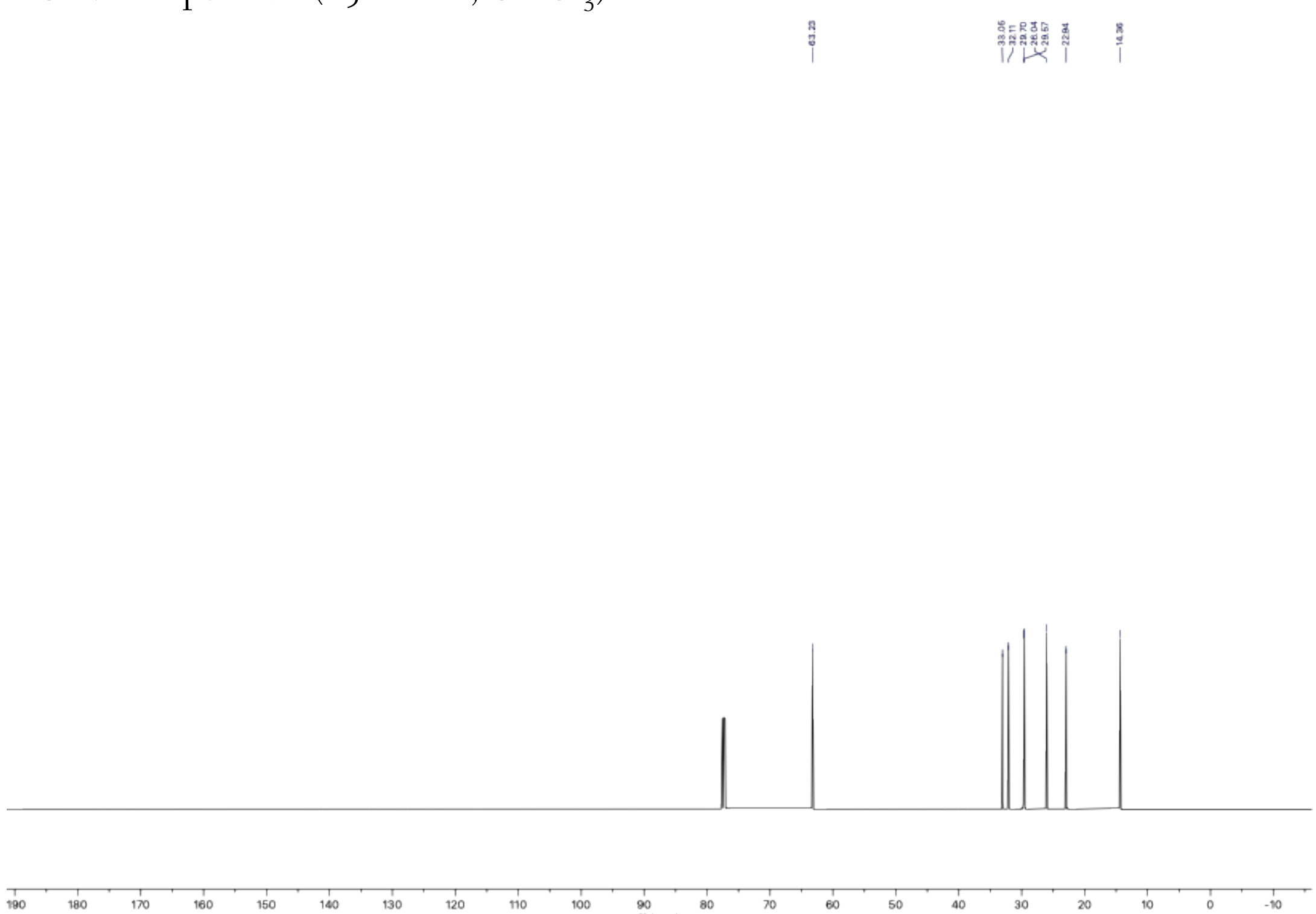

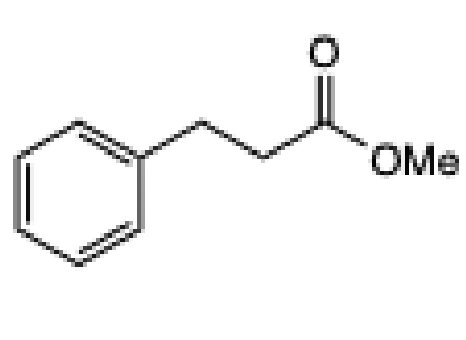

6k

^{1}H NMR spectrum (500 MHz, $CDCl_3$; solvent residue found at 1.1-1.2 ppm):

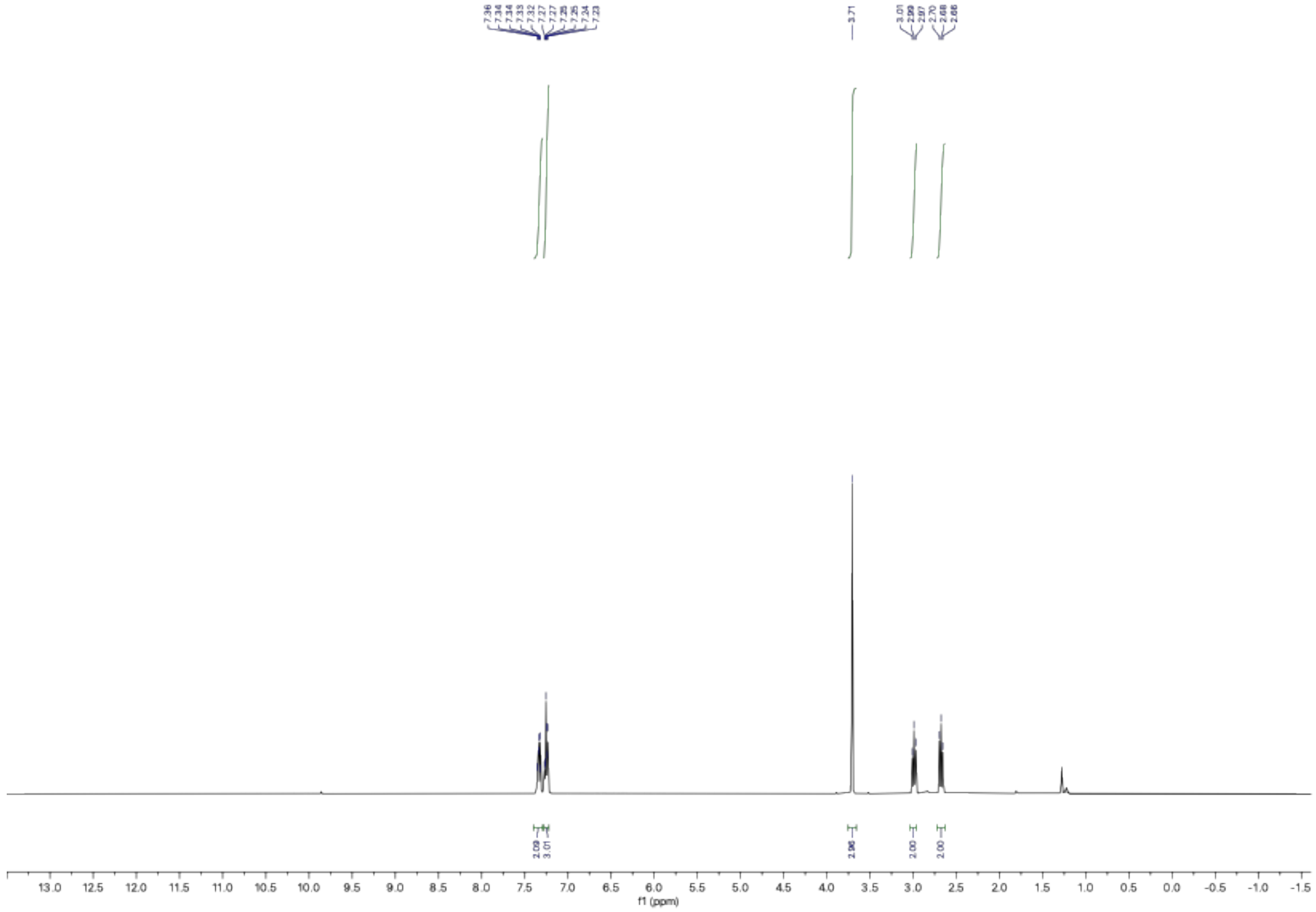

^{13}C NMR spectrum (101 MHz, $CDCl_3$):

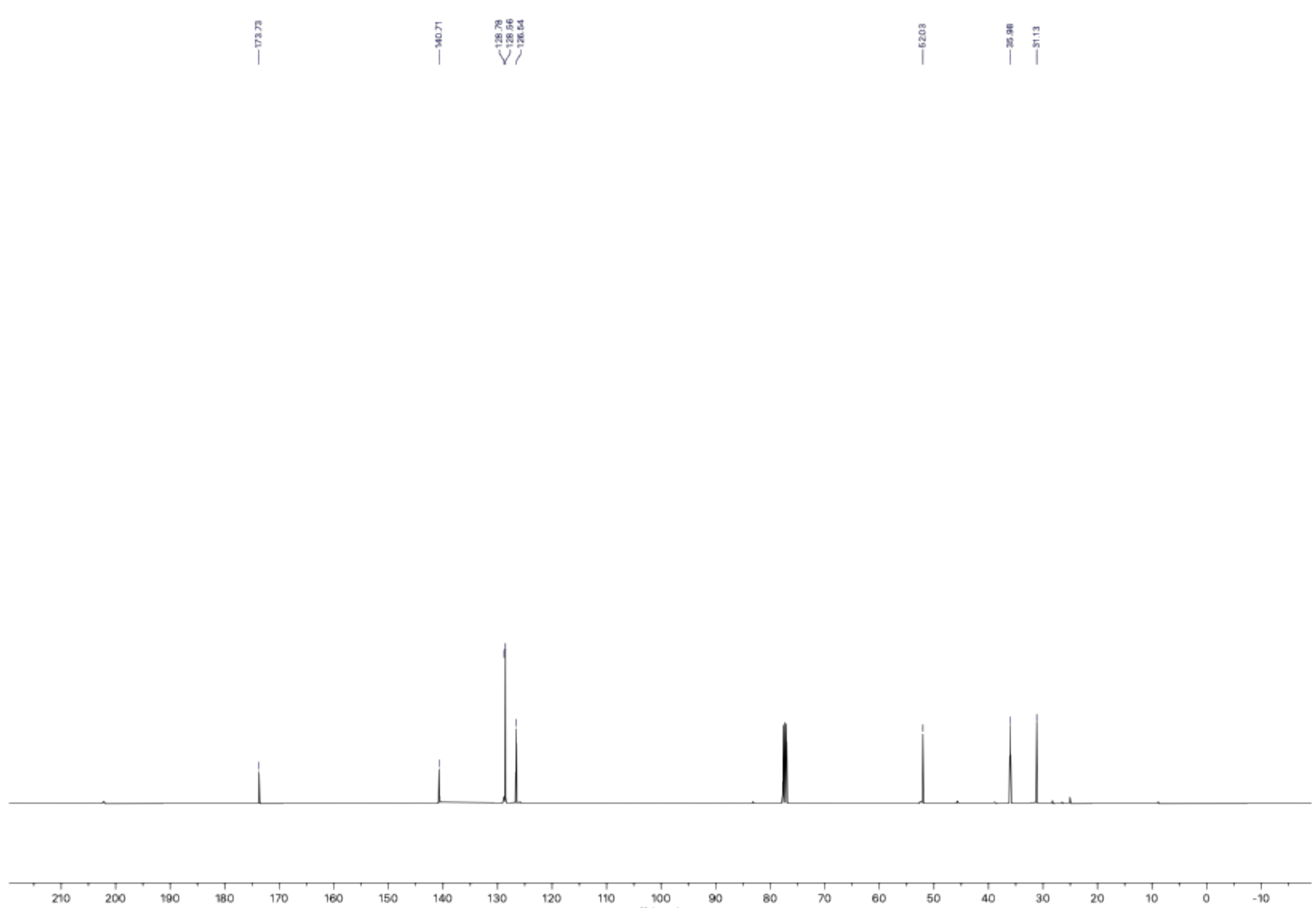

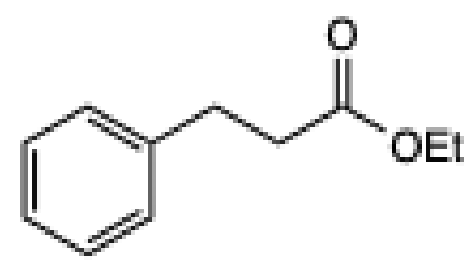

6l (including 15% starting material)

^{1}H NMR spectrum (400 MHz, $CDCl_3$):

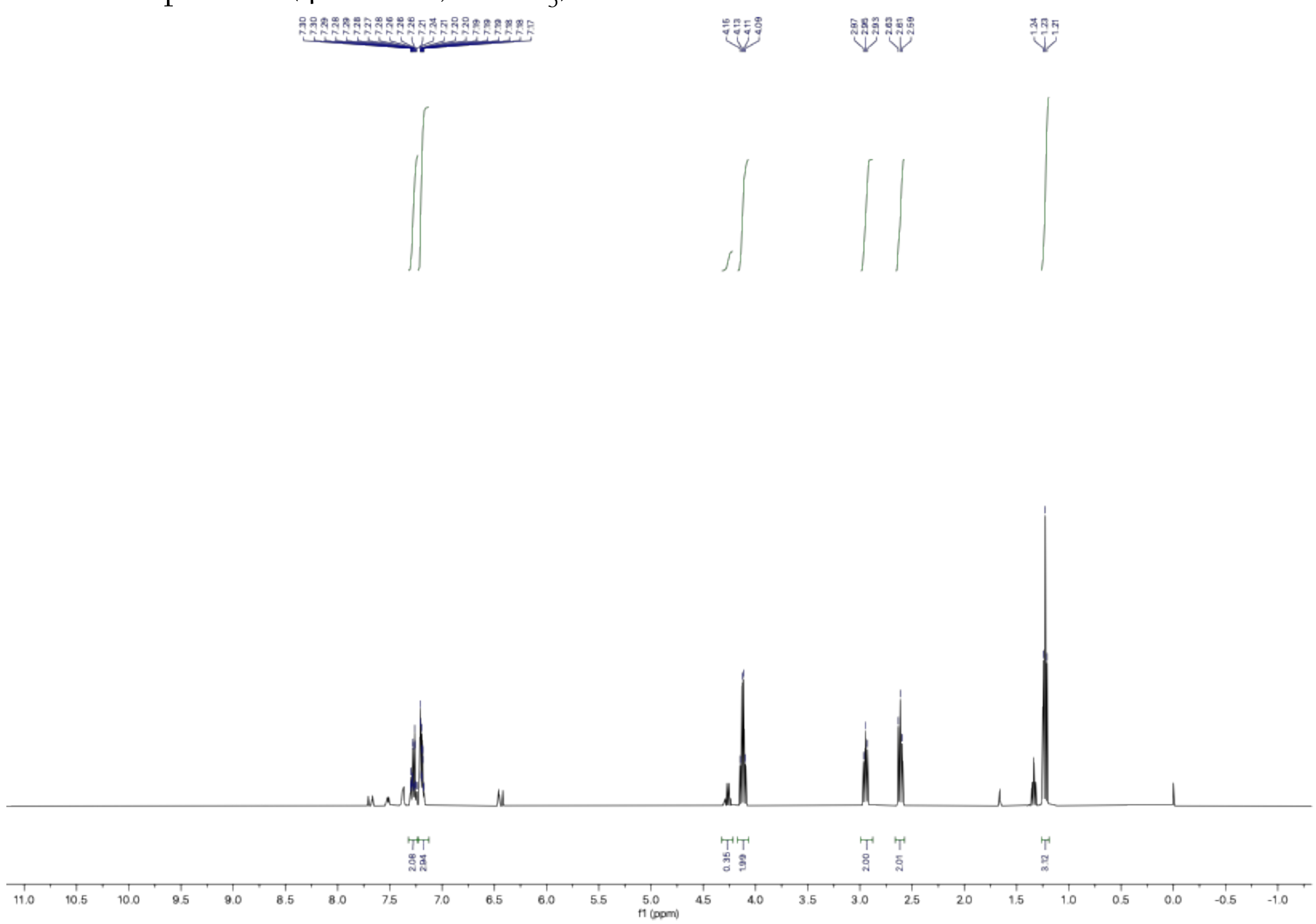

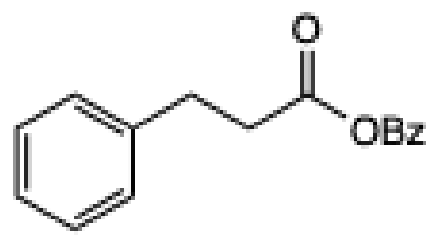

6m

^{1}H NMR spectrum (600 MHz, $CDCl_3$):

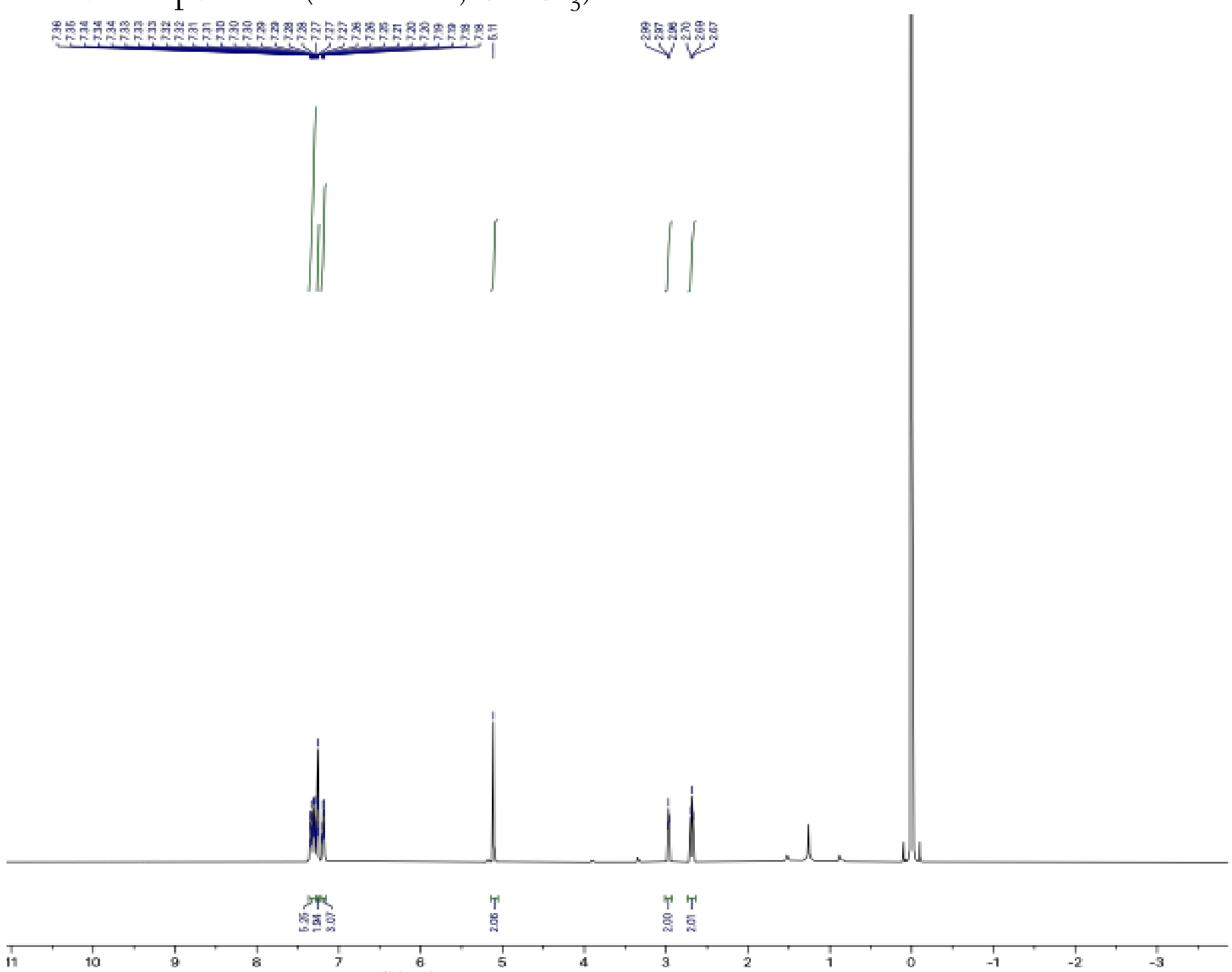

^{13}C NMR spectrum (151 MHz, $CDCl_3$):

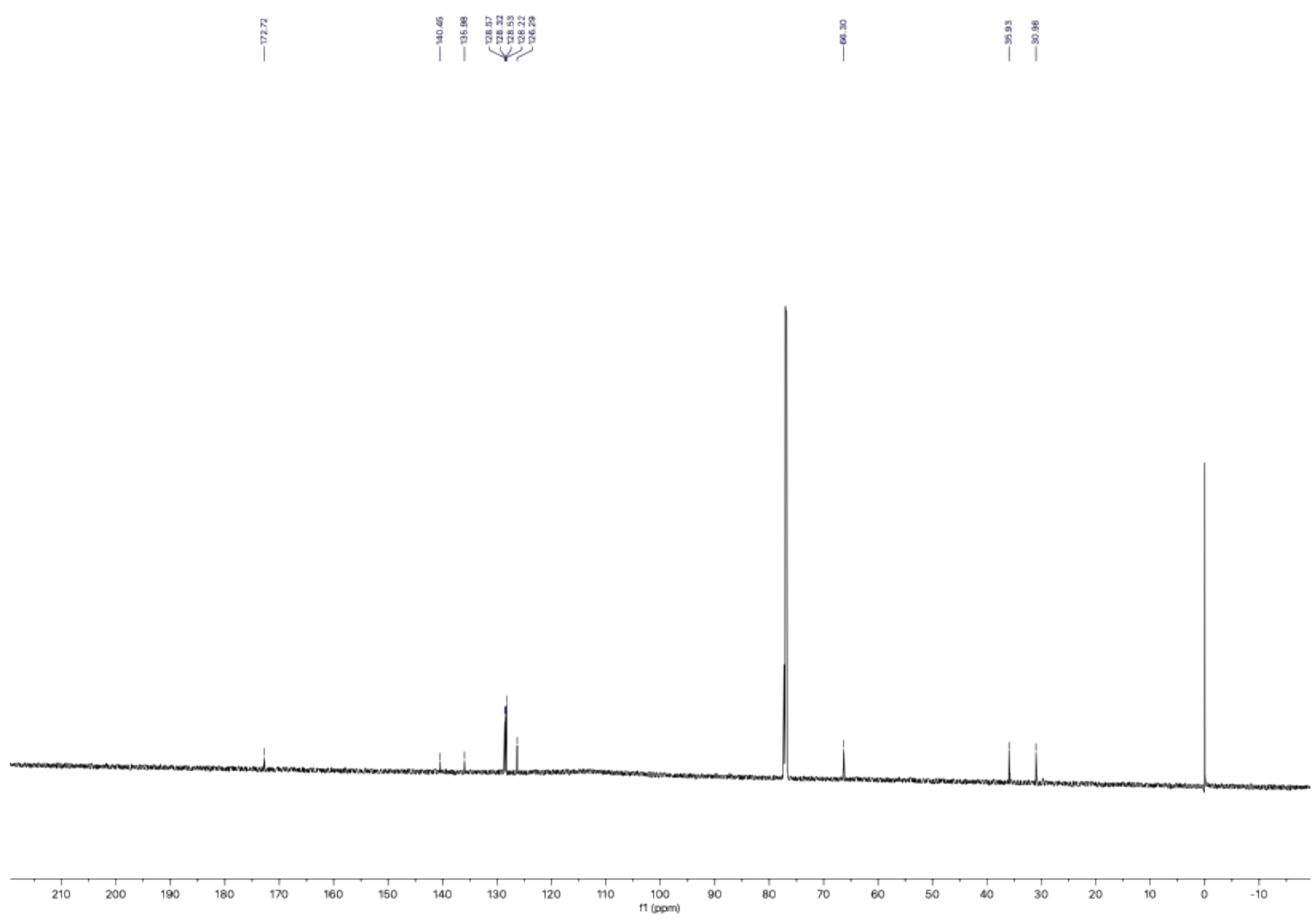

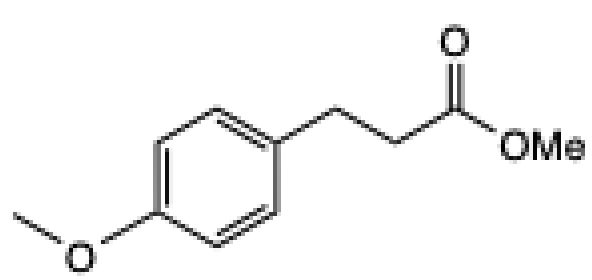

6n

^{1}H NMR spectrum (400 MHz, $CDCl_3$):

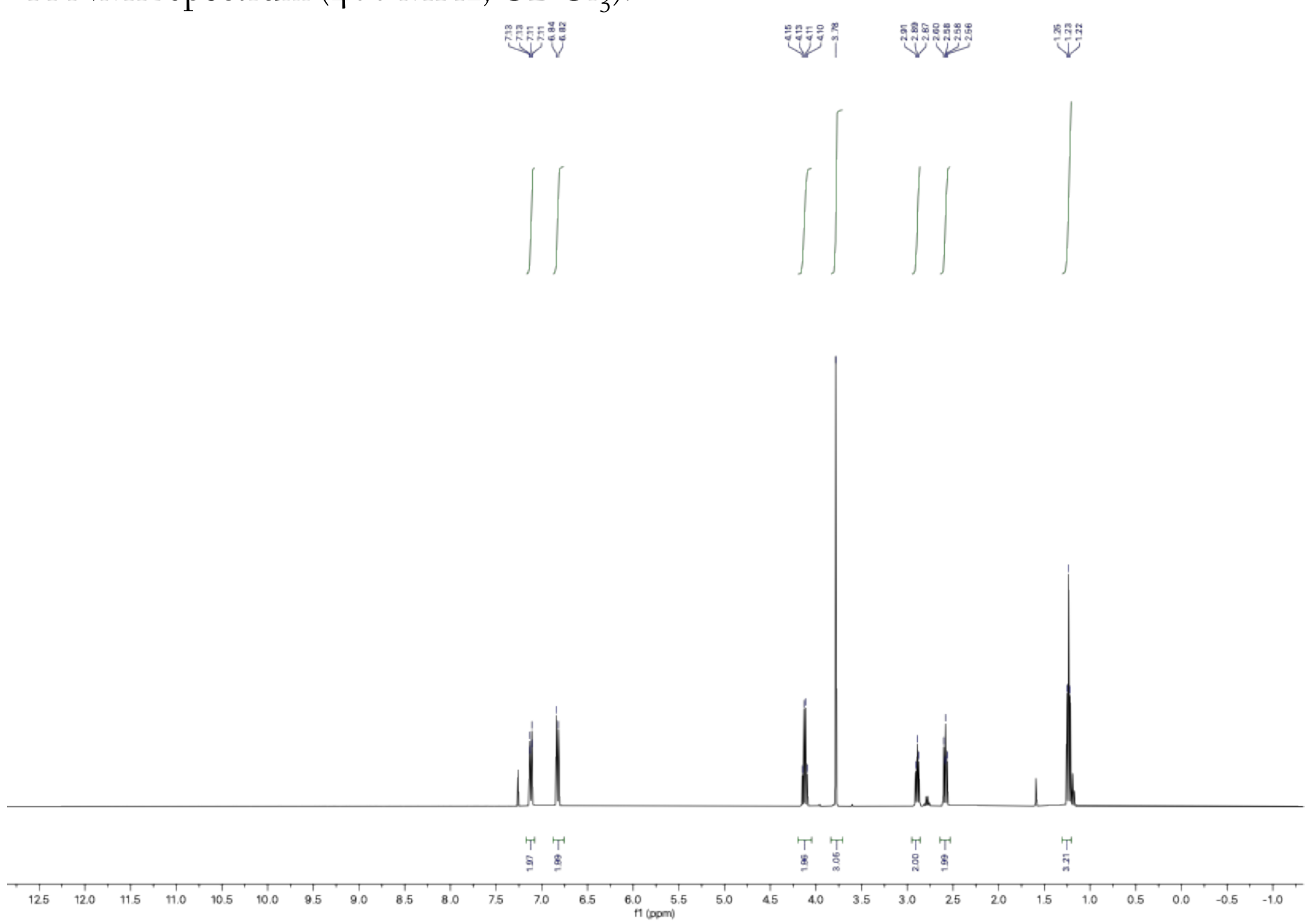

^{13}C NMR spectrum (101 MHz, $CDCl_3$):

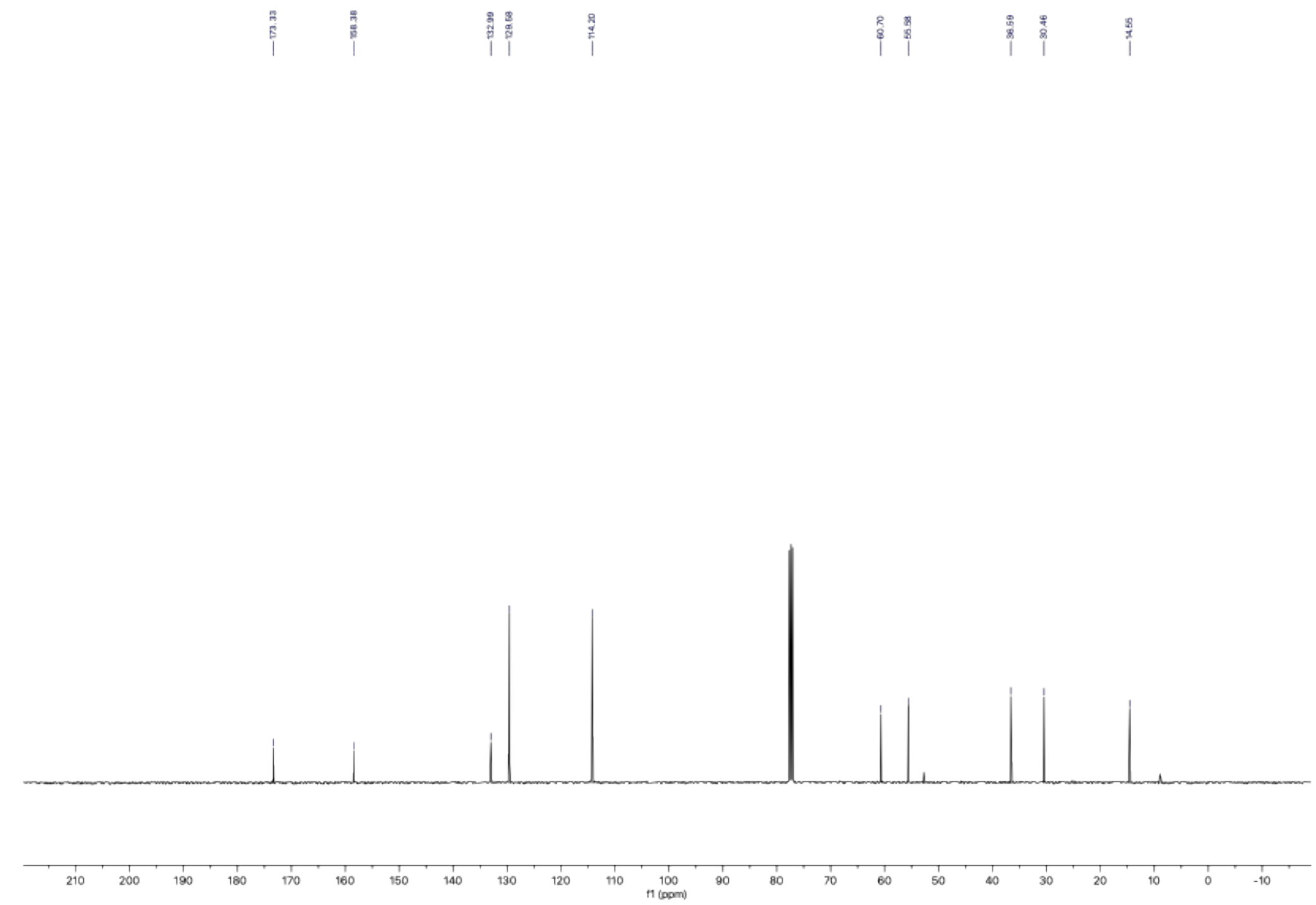

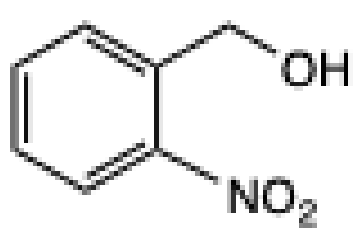

9a

^{1}H NMR spectrum (500 MHz, $CDCl_3$):

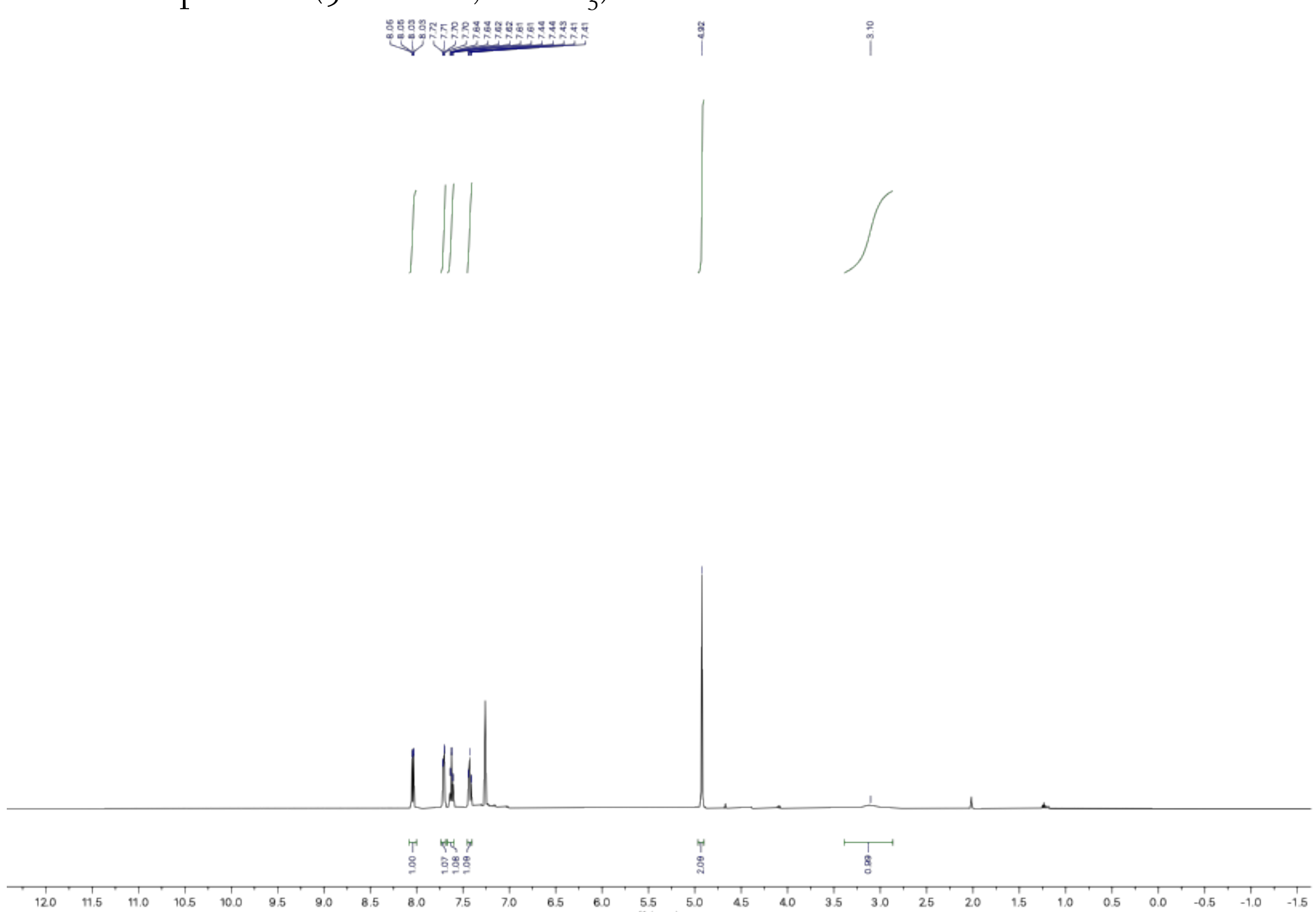

^{13}C NMR spectrum (126 MHz, $CDCl_3$):

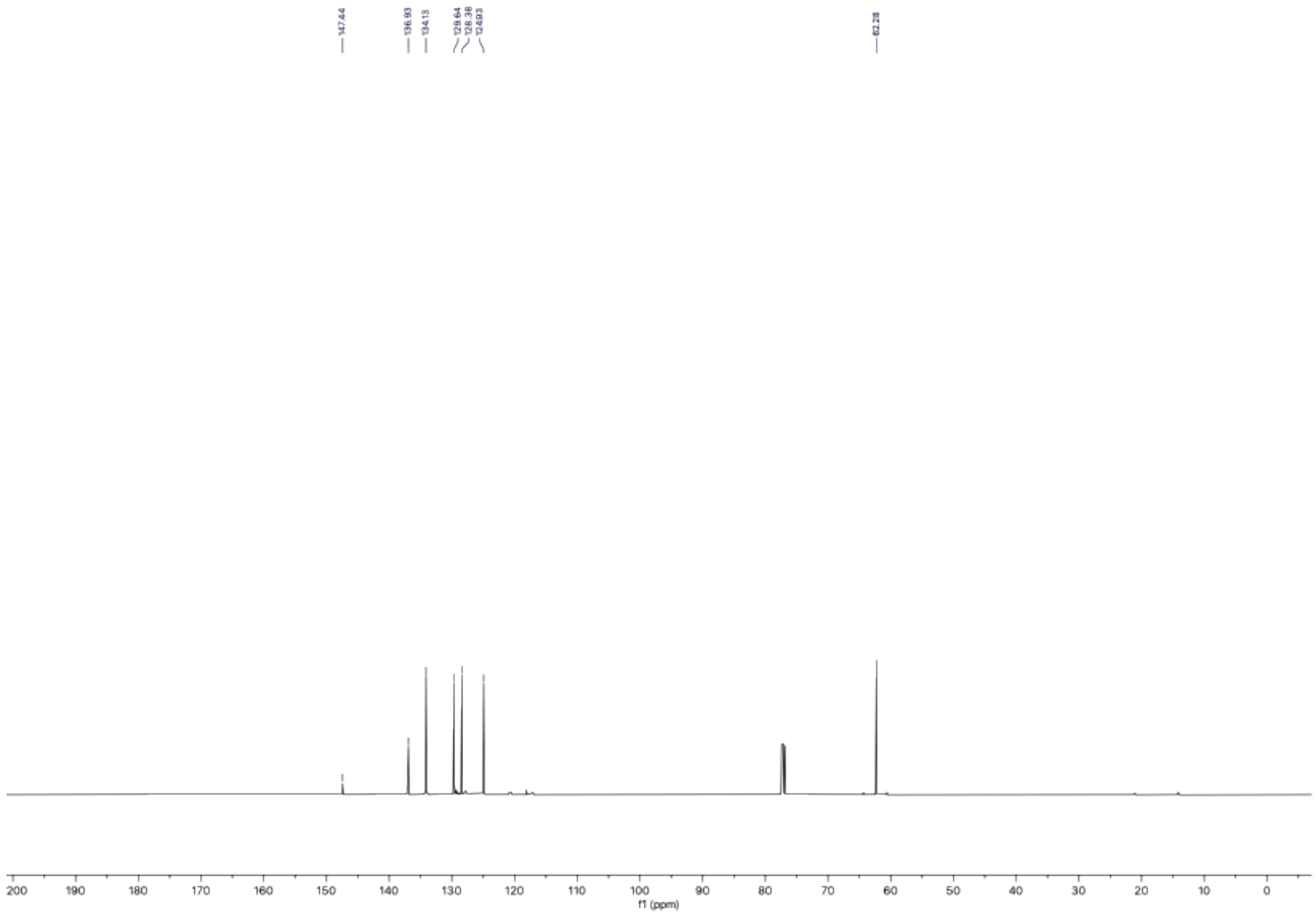

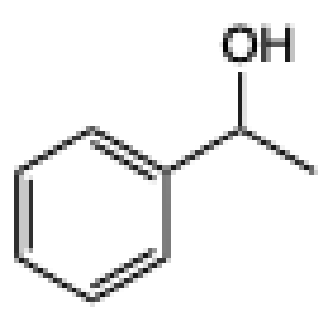

9b

^{1}H NMR spectrum (400 MHz, $CDCl_3$):

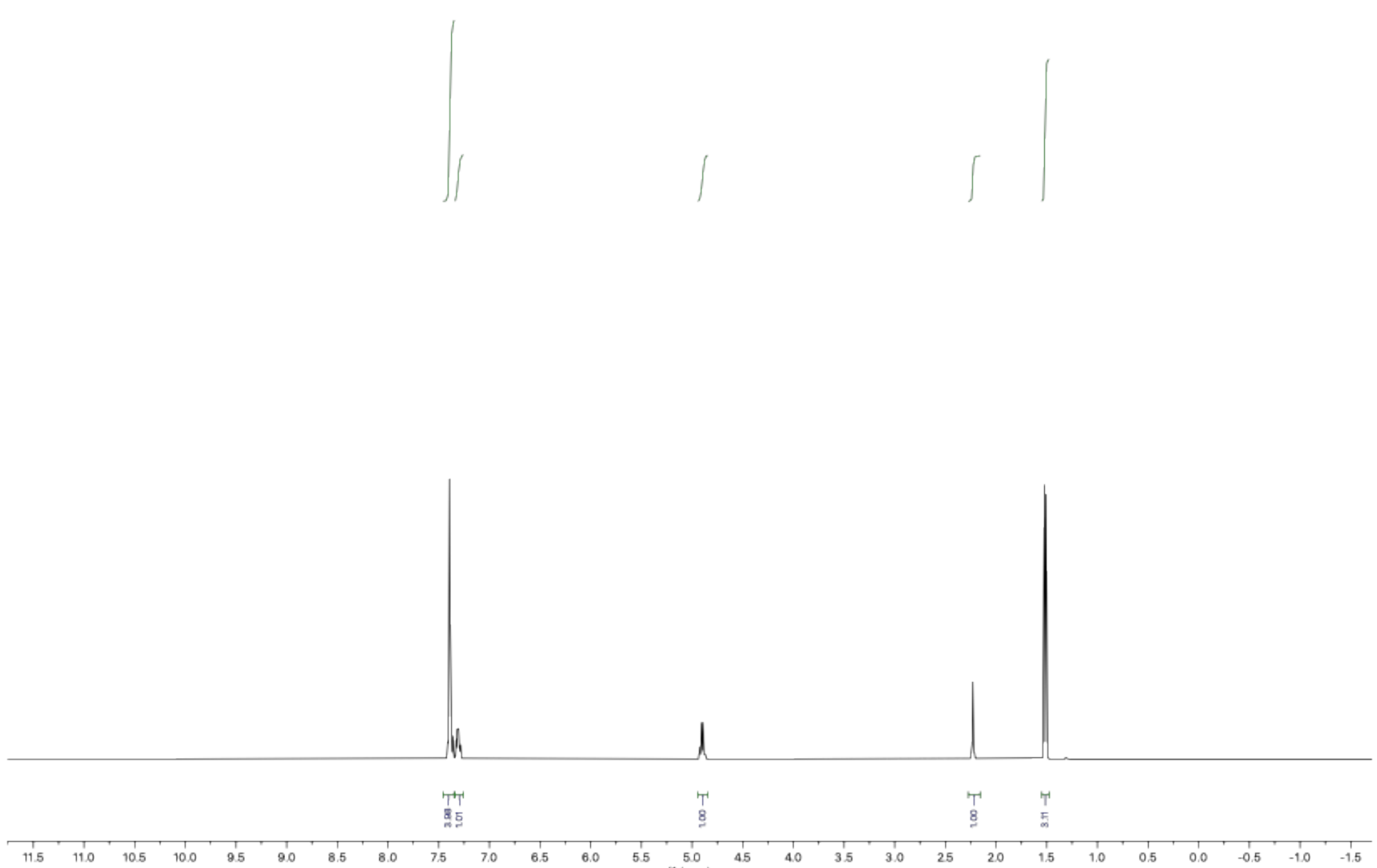

^{13}C NMR spectrum (101 MHz, $CDCl_3$):

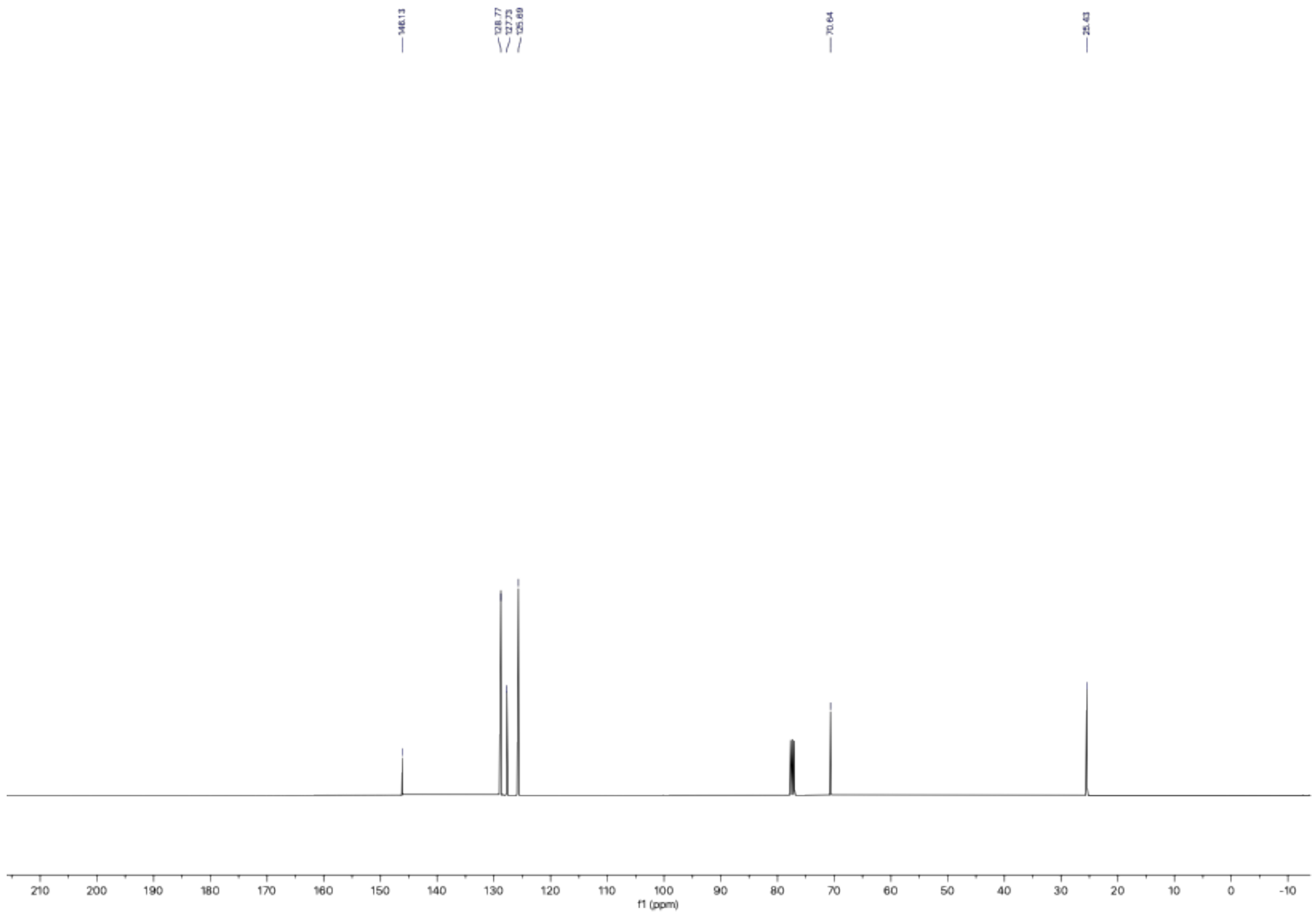

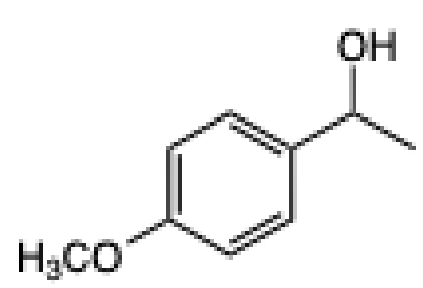

9c

^{1}H NMR spectrum (600 MHz, $CDCl_3$):

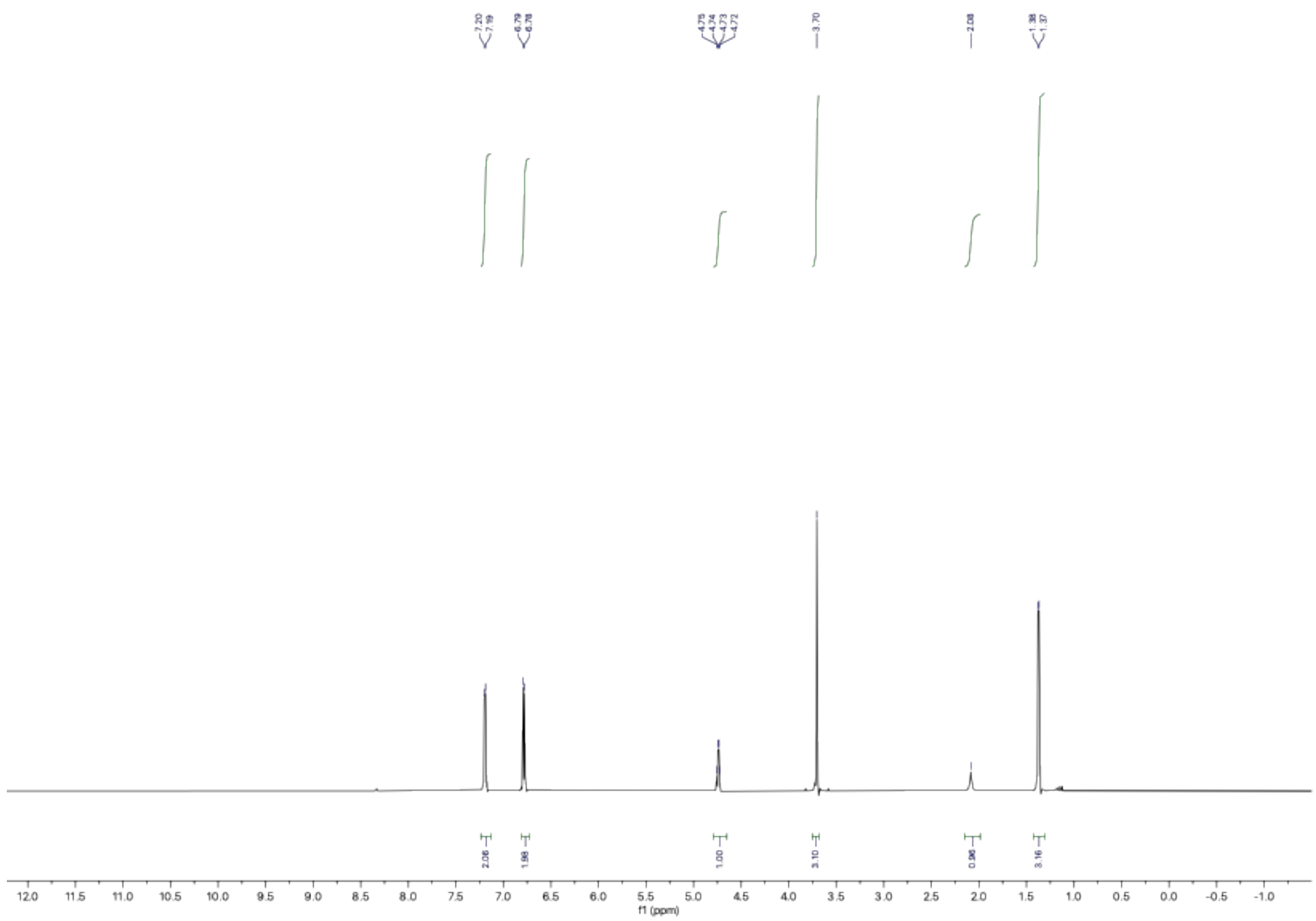

^{13}C NMR spectrum (151 MHz, $CDCl_3$):

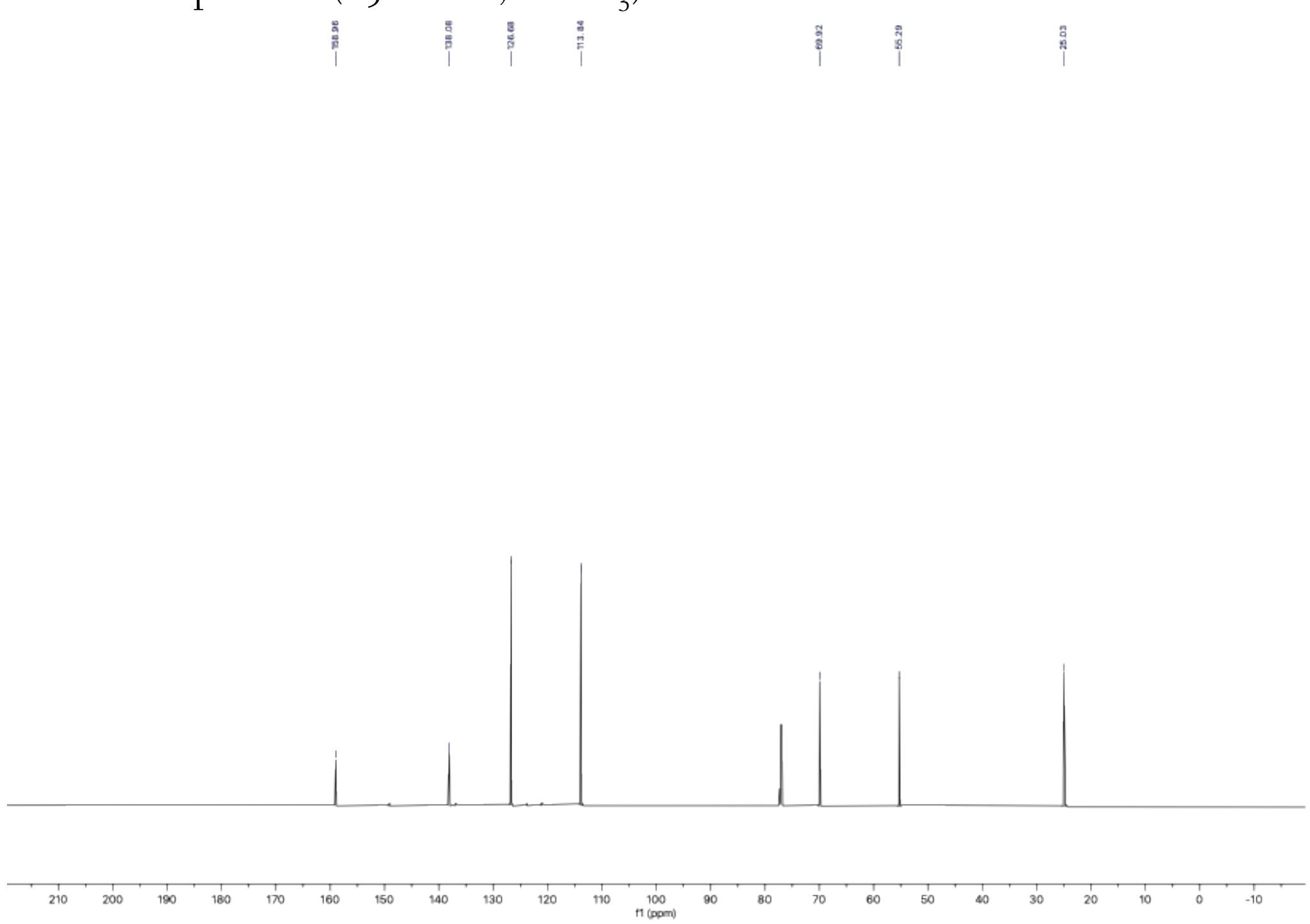

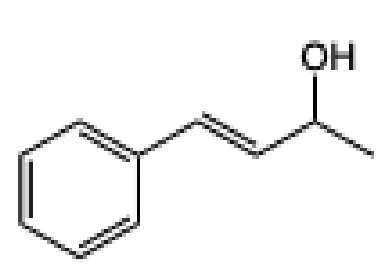

9d

^{1}H NMR spectrum (500 MHz, $CDCl_3$):

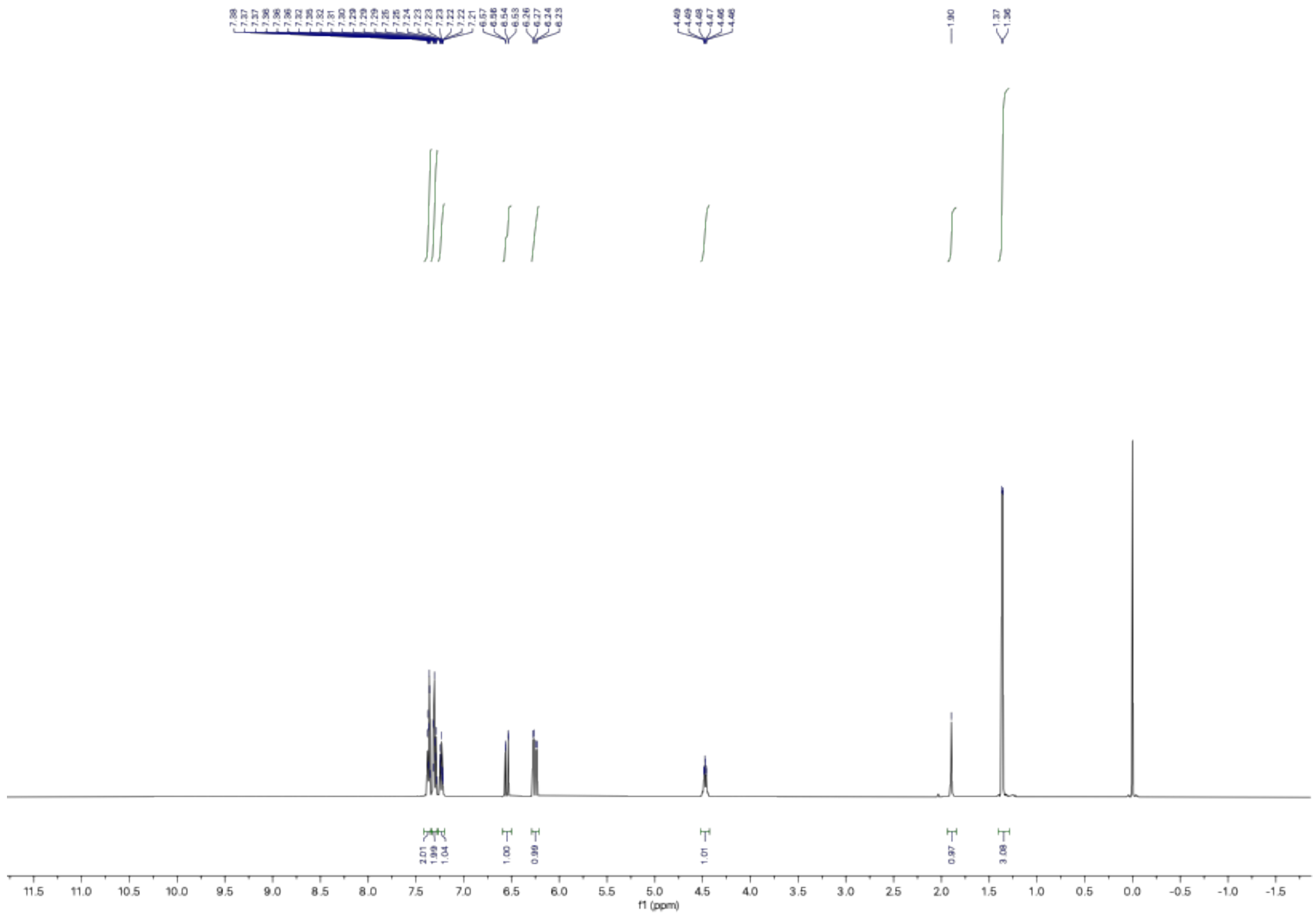

^{13}C NMR spectrum (151 MHz, $CDCl_3$):

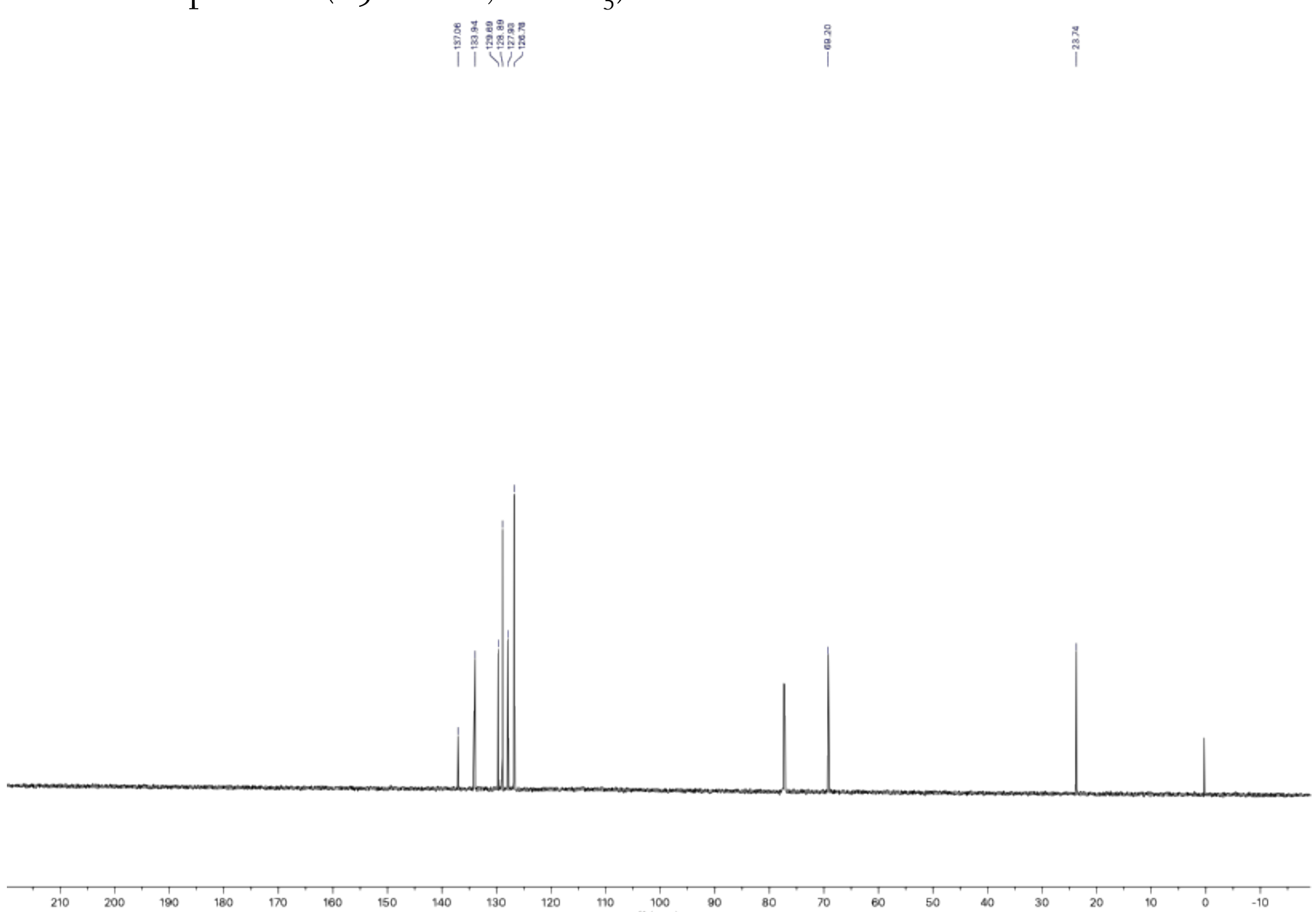

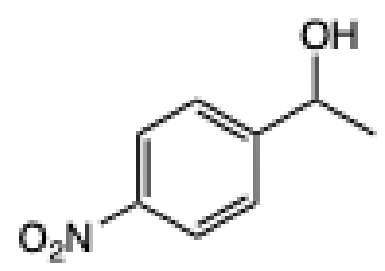

9e

^{1}H NMR spectrum (600 MHz, $CDCl_3$):

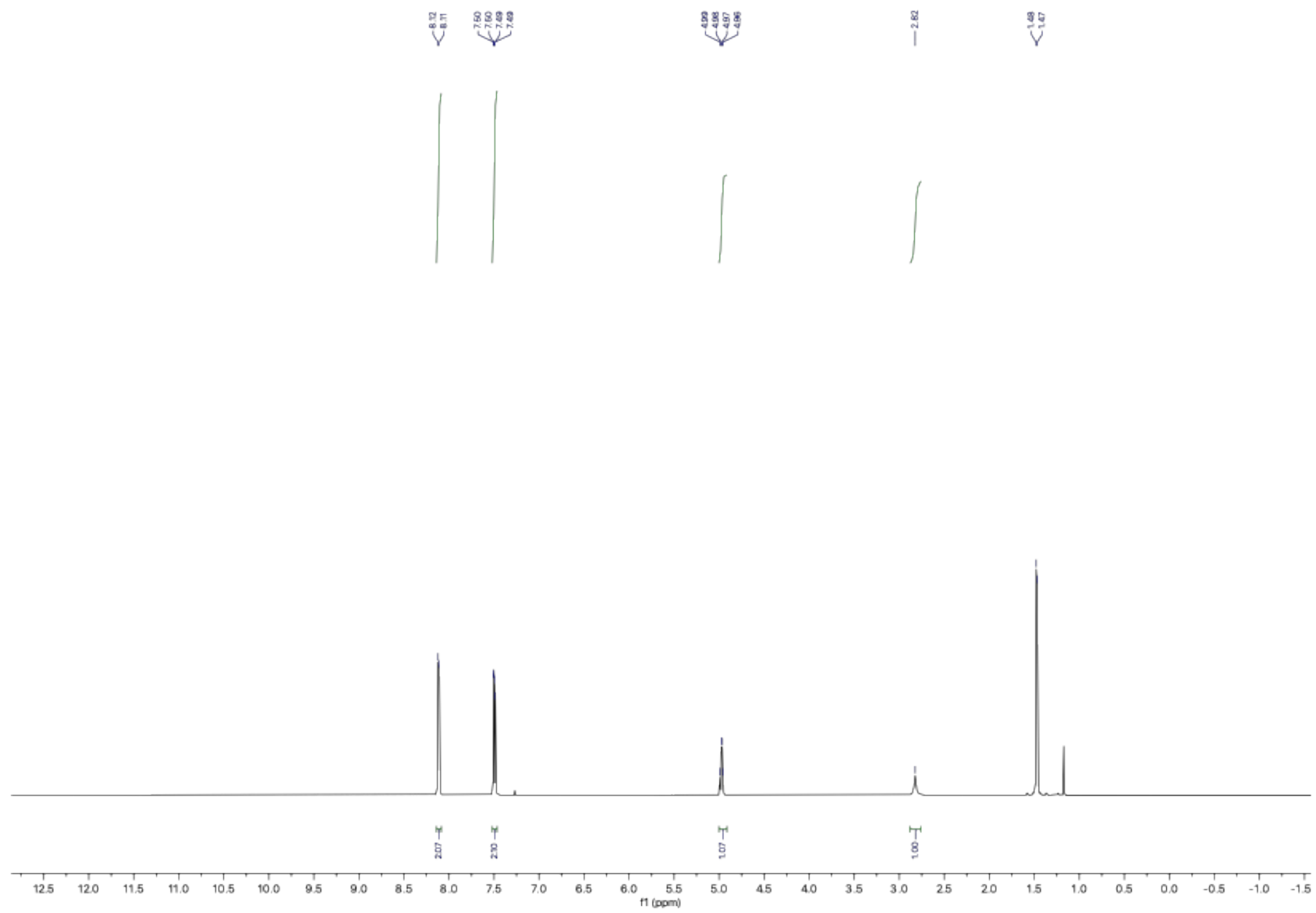

^{13}C NMR spectrum (126 MHz, $CDCl_3$):

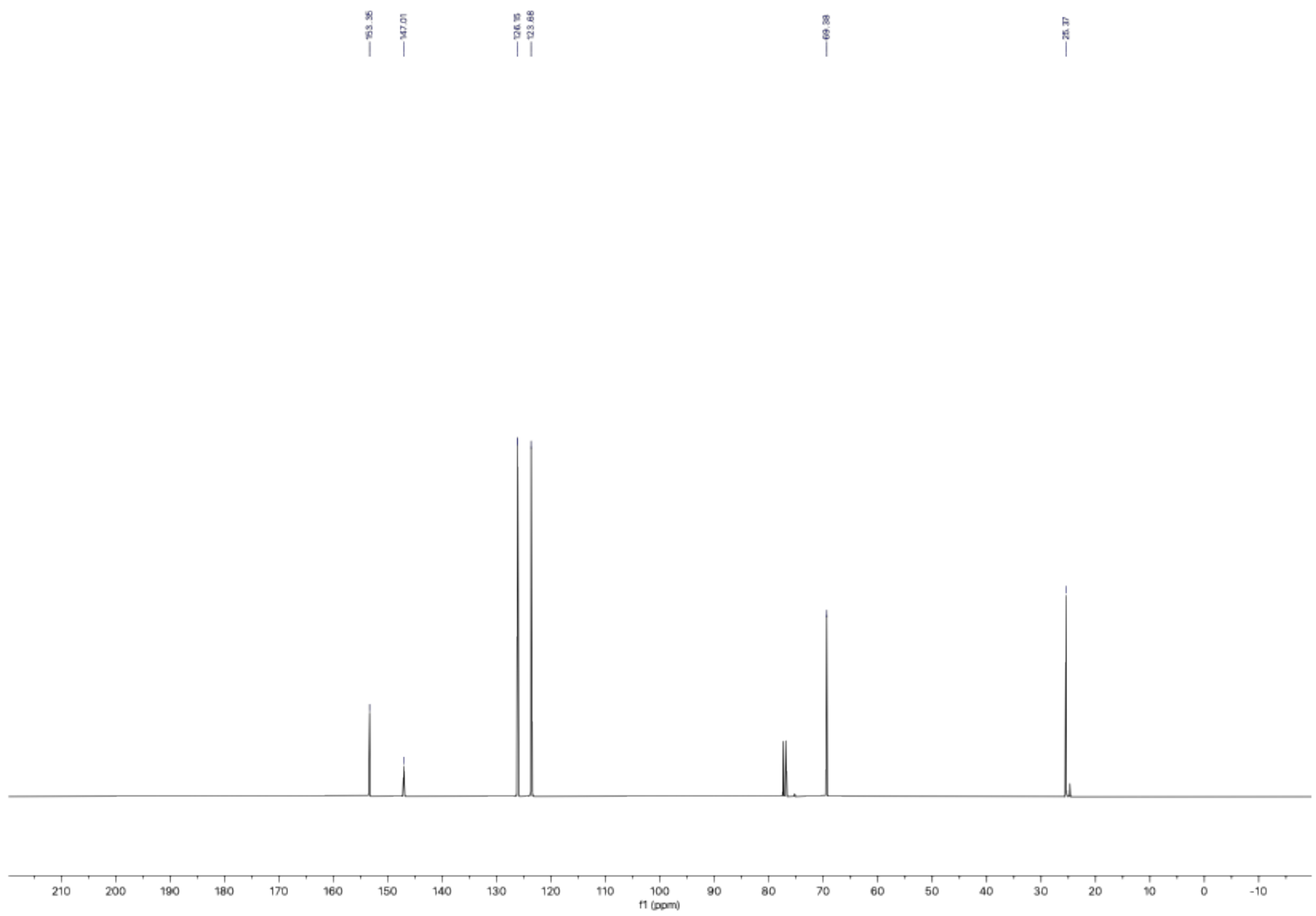

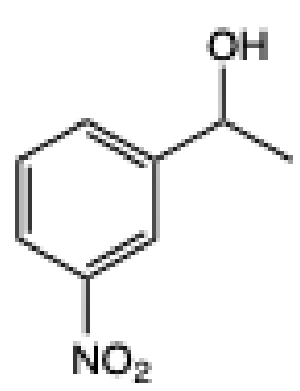

9f

^{1}H NMR spectrum (600 MHz, $CDCl_3$):

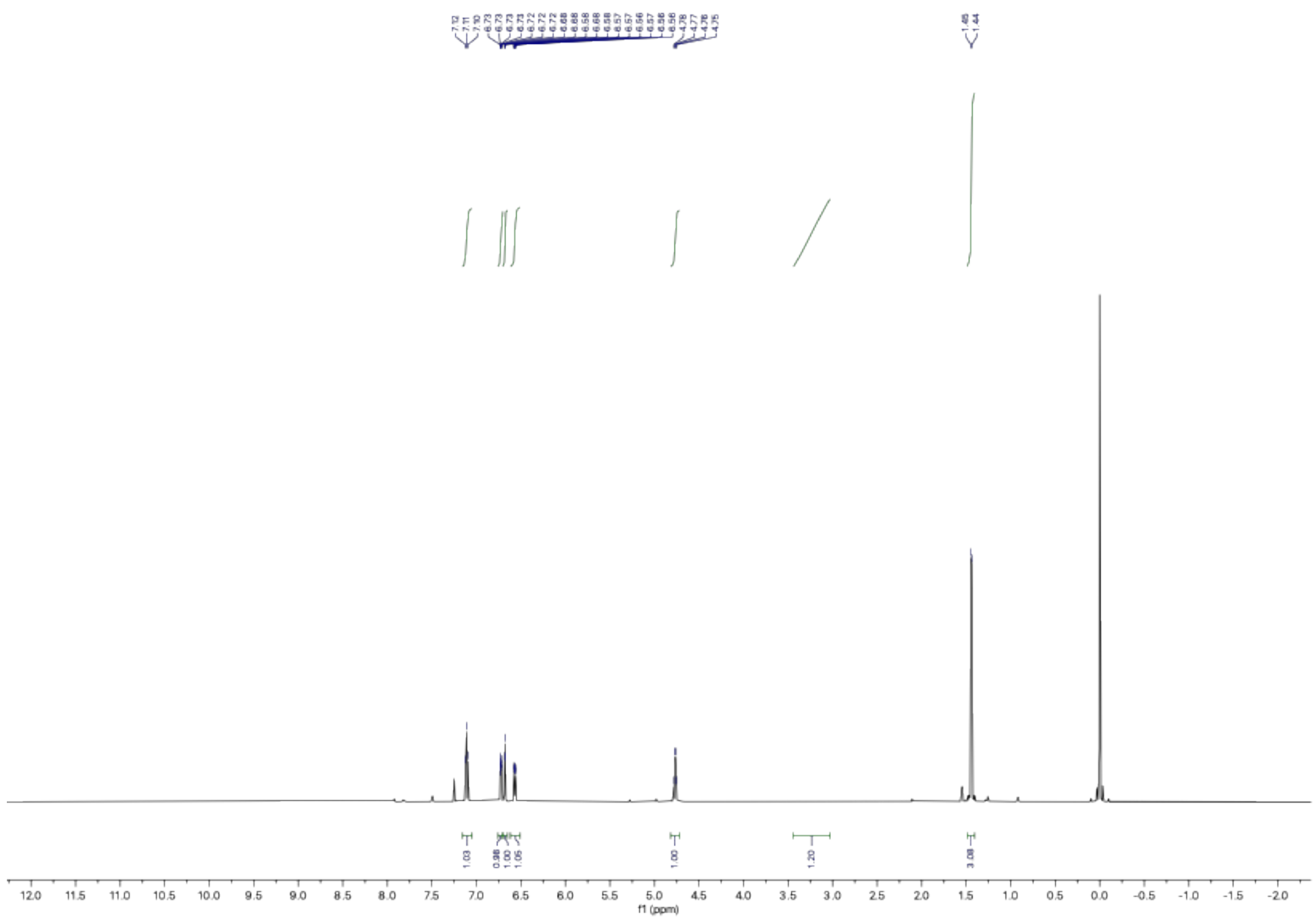

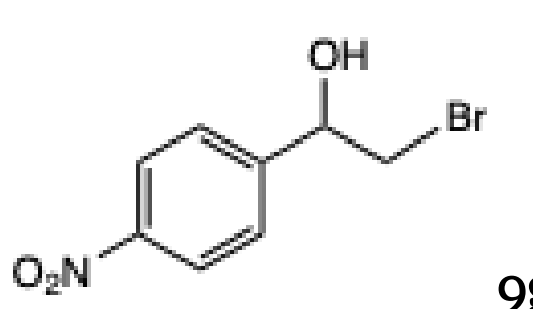

9g

^1H NMR spectrum (500 MHz, $CDCl_3$):

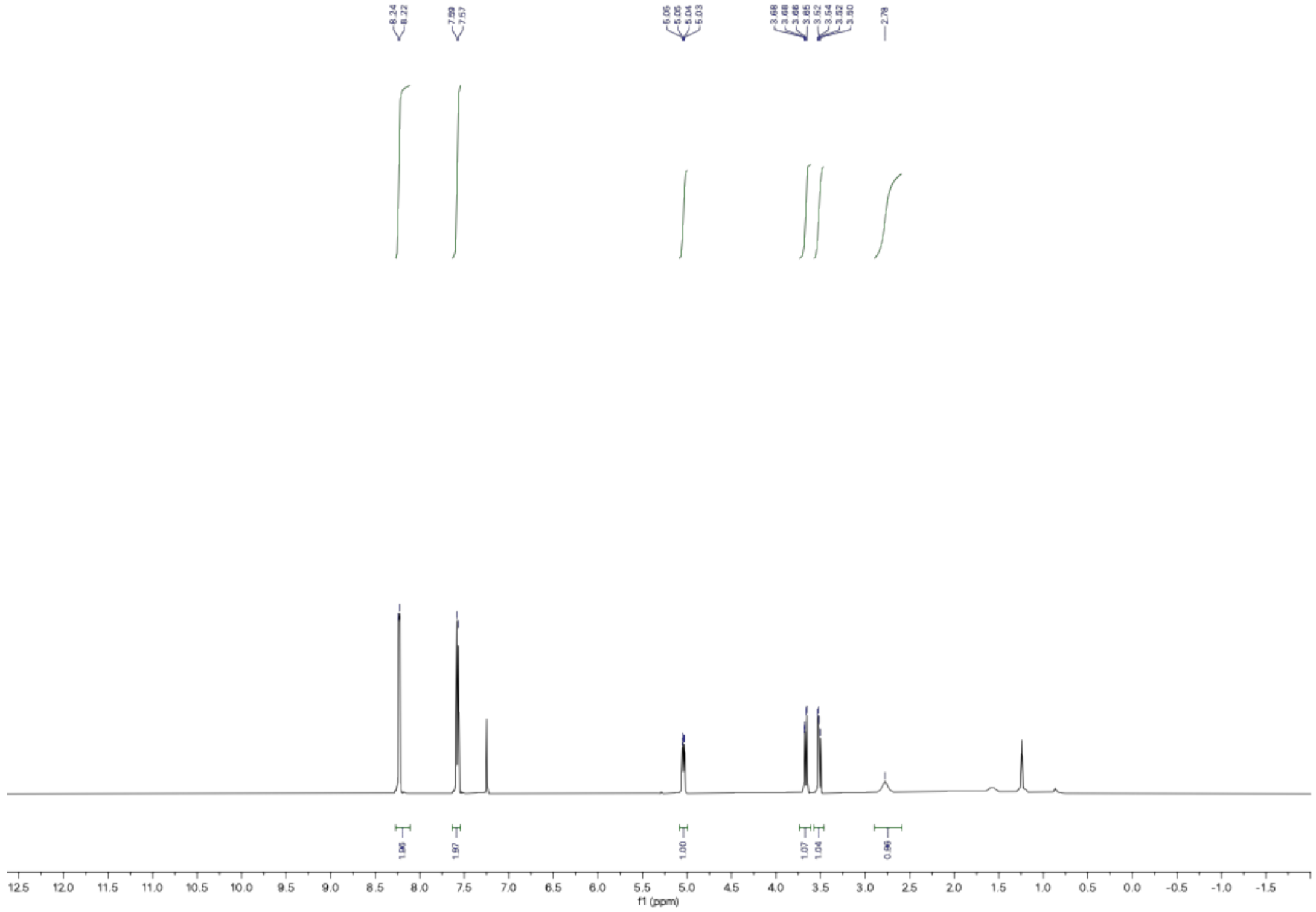

^{13}C NMR spectrum (126 MHz, $CDCl_3$):

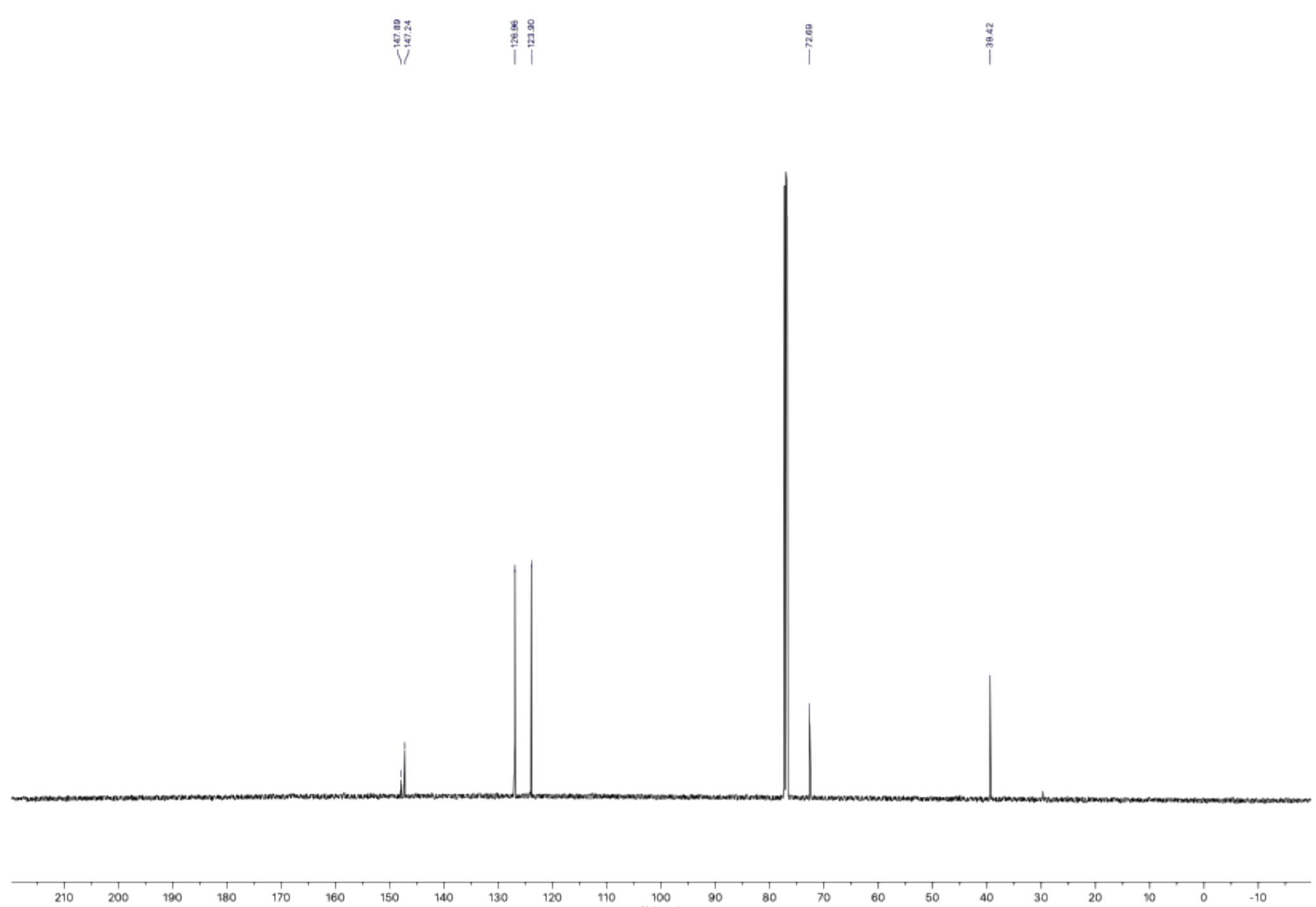

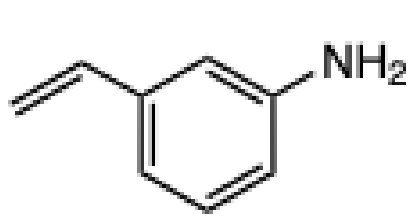

10

^{1}H NMR spectrum (600 MHz, $CDCl_3$):

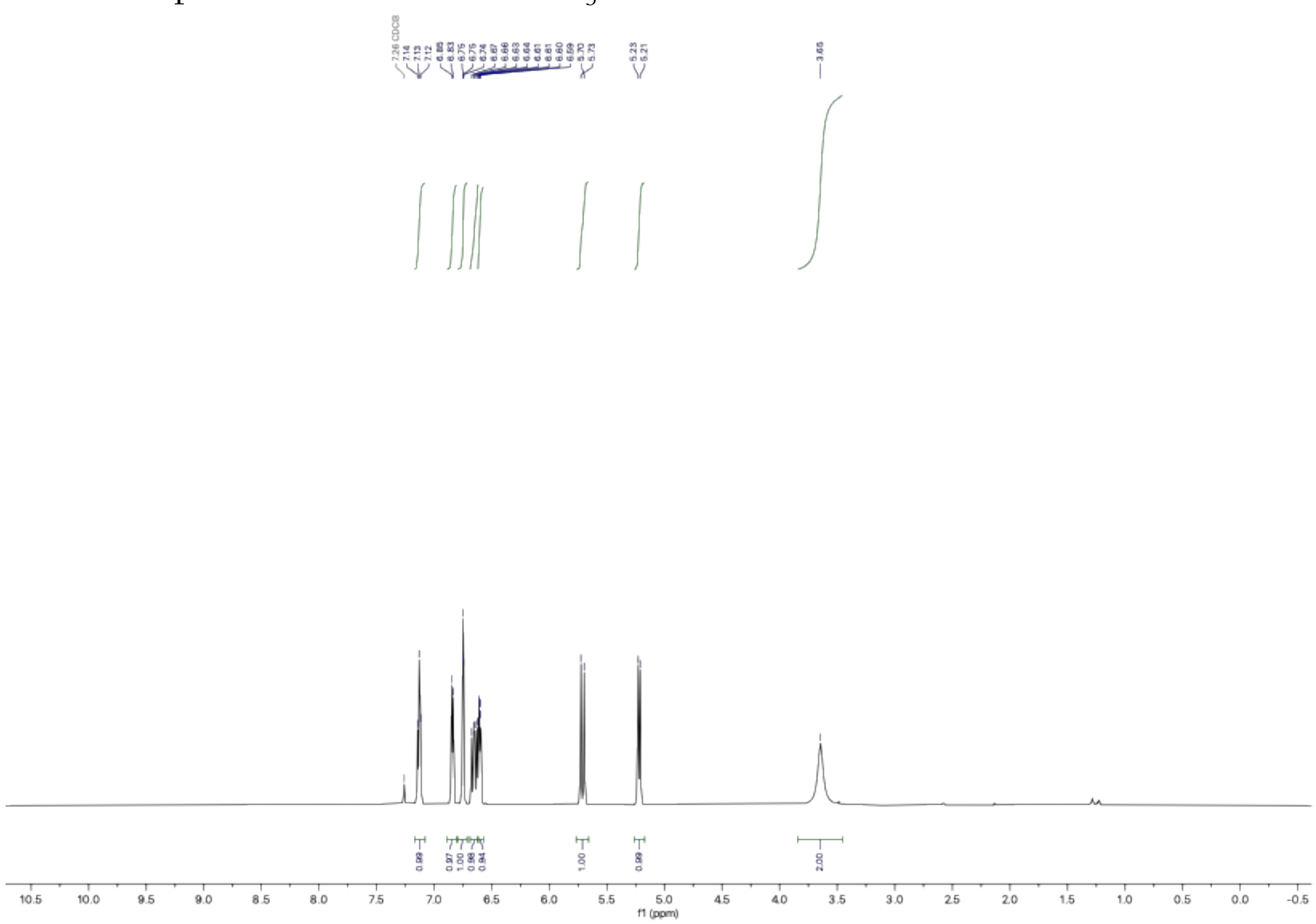

^{13}C NMR spectrum (126 MHz, $CDCl_3$):

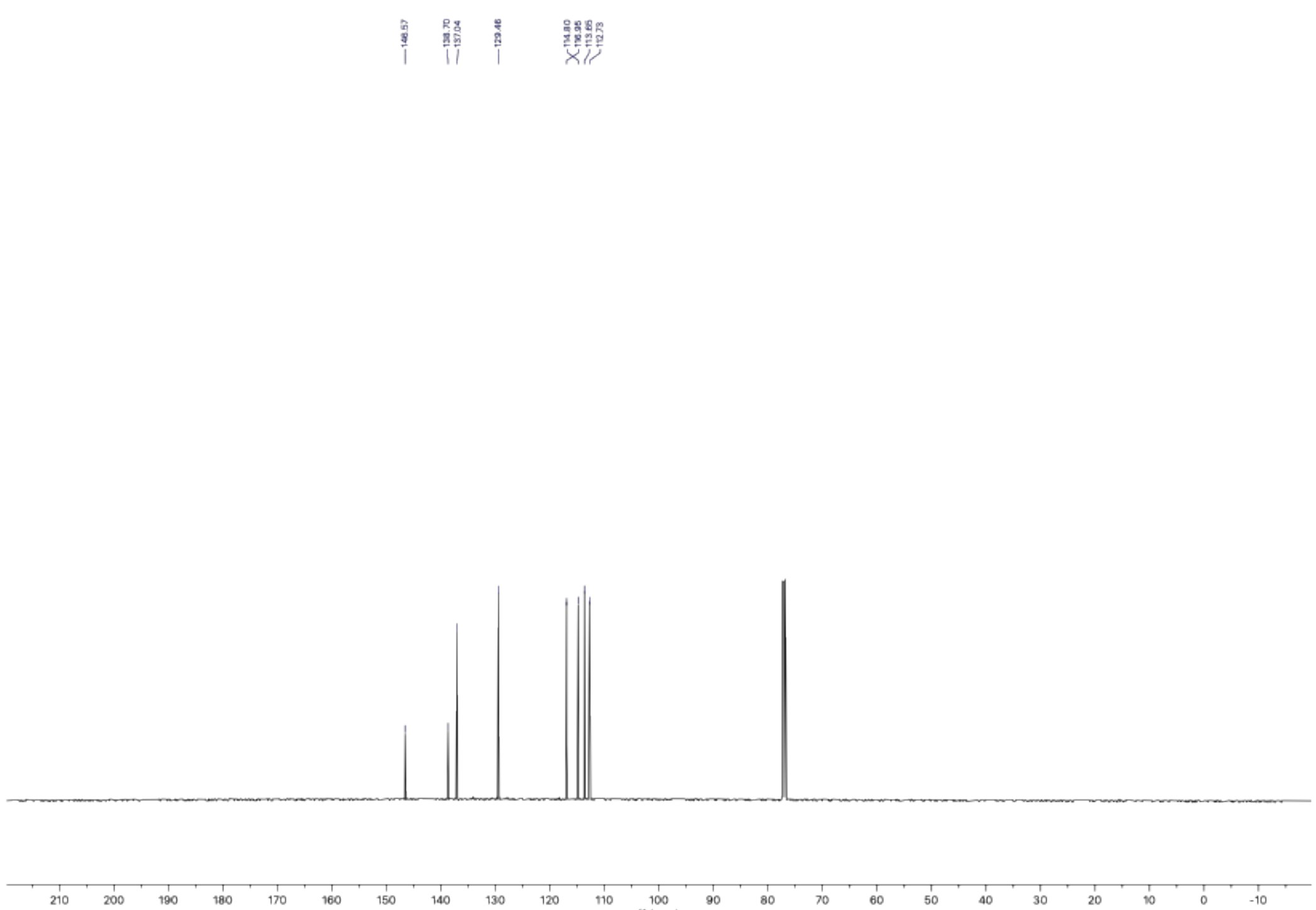

References

1. G. Zhang, J. Cheng, K. Davis, M. G. Bonifacio and C. Zajaczkowski, Practical and selective hydroboration of aldehydes and ketones in air catalysed by an iron (II) coordination polymer, *Green Chem.*, 2019, 21, 1114–1121.

2. G. Zhang, H. Zeng, S. Li, J. Johnson, Z. Mo, M. C. Neary and S. Zheng, 1-D manganese (II)-terpyridine coordination polymers as precatalysts for hydrofunctionalisation of carbonyl compounds, *Dalton Trans.*, 2020, 49, 2610–2615.

3. W. Liu, W. Li, A. Spannenberg, K. Junge and M. Beller, Iron-catalysed regioselective hydrogenation of terminal epoxides to alcohols under mild conditions, *Nat. Catal.*, 2019, 2, 523–528.

4. T. Vielhaber, K. Faust, T. Bögl, W. Schöfberger and C. Topf, A triphos-modified tungsten piano-stool complex for the homogeneous (conjugate) hydrogenation of ketones and esters, *J. Catal.*, 2022, 416, 352–363.

5. M. Fujii, N. Ueno, K. Mitsudo, E. Sato and S. Suga, Transformation of α, β-Unsaturated Aldehydes with a Small Amount of Electricity: Cyanosilylation, Isomerization, and Nucleophilic Addition, *Org. Lett.*, 2025, 27, 6953–6958.

6. S. B. Khatal, M. S. Padmor, M. Mariet and S. Pratihar, Room temperature transfer hydrogenation of aldehydes using methanol catalyzed by the iridium (iii) pyridylidene–indole complex, *Chem. Commun.*, 2025, 61, 8063–8066.

7. J. Gunasekaran, P. Muthurathinam, K. Venkatachalam, N. Venkatesan, R. Varadarasu, L. G. Alves, A. M. Martins and S. B. Kuppannan, Transfer hydrogenation of ketones to secondary alcohols catalyzed by new Ruthenium (II) Heterocyclic hydrazone complexes, *J. Organomet. Chem.*, 2025, 123905.

8. L. H. Andrade, R. Polak, A. L. Porto, I. H. Schoenlein-Crusius and J. V. Comasseto, Application of bioreduction by microorganisms in the enantioselective synthesis of alpha-substituted-1-phenylethanols, *Lett. Org. Chem.*, 2006, 3, 613–618.

9. M. Li, S. Chen, Q. Jiang, Q. Chen, X. Wang, Y. Yan, J. Liu, C. Lv, W. Ding and X. Guo, Origin of the activity of Co–N–C catalysts for chemoselective hydrogenation of nitroarenes, *ACS Catal.*, 2021, 11, 3026–3039.
